# Scaled Process Priors for Bayesian Nonparametric Estimation of the Unseen Genetic Variation

**DOI:** 10.1080/01621459.2022.2115918

**Published:** 2022-09-29

**Authors:** Federico Camerlenghi, Stefano Favaro, Lorenzo Masoero, Tamara Broderick

**Affiliations:** a Department of Economics, Management and Statistics, University of Milano-Bicocca, Milan, Italy; b Collegio Carlo Alberto, Torino, Italy; c BIDSA, Bocconi University, Milano, Italy; d Department of Economics and Statistics, University of Torino, Torino, Italy; e Department of Electrical Engineering and Computer Science, CSAIL, Massachusetts Institute of Technology, Cambridge, MA

**Keywords:** Bayesian nonparametrics, Beta process prior, Completely random measure, Genetic variation, Predictive distribution, Scaled process prior, Stable process, Unseen-features problem

## Abstract

There is a growing interest in the estimation of the number of unseen features, mostly driven by biological applications. A recent work brought out a peculiar property of the popular completely random measures (CRMs) as prior models in Bayesian nonparametric (BNP) inference for the unseen-features problem: for fixed prior’s parameters, they all lead to a Poisson posterior distribution for the number of unseen features, which depends on the sampling information only through the sample size. CRMs are thus not a flexible prior model for the unseen-features problem and, while the Poisson posterior distribution may be appealing for analytical tractability and ease of interpretability, its independence from the sampling information makes the BNP approach a questionable oversimplification, with posterior inferences being completely determined by the estimation of unknown prior’s parameters. In this article, we introduce the stable-Beta scaled process (SB-SP) prior, and we show that it allows to enrich the posterior distribution of the number of unseen features arising under CRM priors, while maintaining its analytical tractability and interpretability. That is, the SB-SP prior leads to a negative Binomial posterior distribution, which depends on the sampling information through the sample size and the number of distinct features, with corresponding estimates being simple, linear in the sampling information and computationally efficient. We apply our BNP approach to synthetic data and to real cancer genomic data, showing that: (i) it outperforms the most popular parametric and nonparametric competitors in terms of estimation accuracy; (ii) it provides improved coverage for the estimation with respect to a BNP approach under CRM priors. Supplementary materials for this article are available online.

## 1 Introduction

The problem of estimating the number of unseen features generalizes the popular unseen-species problem (Orlitsky, Suresh, and Wu 2016), and its importance has grown dramatically in recent years, driven by applications in biological sciences (Ionita-Laza, Lange, and Laird 2009; Gravel 2014; Zou et al. 2016; Chakraborty et al. 2019). Consider a generic population in which each individual is endowed with a finite collection of W-valued features, with W possibly being an infinite space, and denote by *p_i_* the probability that an individual has feature $w_{i}\in \text{W}$ for i≥1. The unseen-features problem assumes N≥1 observable random samples $Z_{1:N}=(Z_{1},\ldots ,Z_{N})$ from the population, such that $Z_{n}={\left(A_{n,i}\right)}_{i\geq 1}$ are independent Bernoulli random variables with unknown parameters ${\left(p_{i}\right)}_{i\geq 1}$. Then, the goal is to estimate the number of hitherto unseen features that would be observed if M≥1 additional samples were collected, that is,

$$U=\sum_{i\geq 1}1\left(\sum_{n=1}^{N}A_{n,i}=0\right)1\left(\sum_{m=1}^{M}A_{N+m,i}>0\right),$$

with 1 being the indicator function. The unseen-species problem arises under the assumption that each individual is endowed with only one feature, that is, a species. A wide range of approaches have been developed to estimate *U*, including Bayesian methods (Ionita-Laza, Lange, and Laird 2009; Masoero et al. 2021), jackknife (Gravel 2014), linear programming (Zou et al. 2016), and variations of Good-Toulmin estimators (Orlitsky, Suresh, and Wu 2016; Chakraborty et al. 2019).

In biological sciences, we may think of individuals as organisms and of features as groups to which organisms belong to, with each group being defined by any difference in the genome relative to a reference genome, that is, a (genetic) variant. In human biology, the estimation of *U* arises in the context of optimal allocation of resources between quantity and quality in genetic experiments: spending resources to sequence a greater number of genomes (quantity), which reveals more about variation across the population, or spending resources to sequence genomes with increased accuracy (quality), which reveals more about individual organisms’ genomes. Accurate estimates of *U* are critical in the experimental pipeline toward the goal of maximizing the usefulness of experiments under the tradeoff between quantity and quality (Ionita-Laza and Laird 2010; Zou et al. 2016). While in human-biology the cost of sequencing has decreased in recent years (Schwarze et al. 2020), the expense remains nontrivial, and it is still critical in fields where scientists work with relatively budgets, for example, nonhuman and nonmodel organisms (Souza et al. 2017). Other applications arise in precision medicine (Momozawa and Mizukami 2020), microbiome analysis (Sanders et al. 2019), single-cell sequencing (Zhang, Ntranos, and Tse 2020) and wildlife monitoring (Johansson et al. 2020).

### 1.1 Our Contributions

We introduce a Bayesian nonparametric (BNP) approach to the unseen-features problem, which relies on a novel prior distribution for the unknown ${\left(p_{i}\right)}_{i\geq 1}$. Completely random measures (CRMs) (Kingman 1992) provide a broad class of nonparametric priors for feature sampling problems, the most popular being the stable-Beta process prior (James 2017; Broderick, Wilson, and Jordan 2018). In a recent work, Masoero et al. (2021) brought out a peculiar feature of CRM priors in the unseen-features problem: they all lead to a Poisson posterior distribution of *U*, given $Z_{1:N}$ and fixed prior’s parameters, which depends on $Z_{1:N}$ only through the sample size *N*. Despite the broadness of the class of CRM priors, such a common Poisson posterior structure makes CRMs not a flexible prior model for the unseen-features problem. While the Poisson posterior distribution may be appealing in principle, making posterior inferences analytically tractable and easy to interpret, its independence from $Z_{1:N}$ makes the BNP approach a questionable oversimplification, with posterior inferences being completely determined by the estimation of the unknown prior’s parameters. A somehow similar scenario occurs in BNP inference for the unseen-species problem under a Dirichlet process (DP) prior (Ferguson 1973), and led to the use of the Pitman-Yor process (PYP) prior (Pitman and Yor 1997) for enriching the posterior distribution of the number of unseen species, while maintaining analytical tractability and interpretability of the DP prior (Lijoi, Mena, and Prünster 2007).

We show that scaled process (SP) priors, first introduced in James, Orbanz, and Teh (2015), allow to enrich the posterior distribution of *U* arising under CRM priors. Under SP priors, we characterize the posterior distribution of *U* as a mixture of Poisson distributions that may include, through the mixing distribution, the whole sampling information in terms of the number of distinct features and their frequencies. While this is appealing in principle, it may be at stake with analytical tractability and interpretability, which are critical for a concrete use of SP priors. Then, we introduce the stable-Beta SP (SB-SP) prior, which provides a sensible tradeoff between the amount of sampling information introduced in the posterior distribution of *U*, and analytical tractability and interpretability of the posterior inferences. In particular, we characterize the SB-SP prior as the sole SP prior for which the posterior distribution of *U*, given $Z_{1:N}$ and fixed prior’s parameters, depends on $Z_{1:N}$ through the sample size *N* and the number *K_N_* of distinct features; the SB-SP may thus be considered as the natural counterpart of the PYP for the unseen-feature problem. Under the SB-SP prior, the posterior distribution of *U*, as well as of a refinement of *U* that deals with the number of unseen rare features, is a negative Binomial posterior distributions, whose parameters depend on *N*, *K_N_* and the prior’s parameters. Corresponding Bayesian estimates of *U*, with respect to a squared loss function, are simple, linear in *K_N_* and computationally efficient.

We present an empirical validation of the effectiveness of our BNP methodology, both on synthetic and real data. As for real data, we consider cancer genomic data, where the goal is to estimate the number of new (genomic) variants to be discovered in future unobservable samples. In cancer genomics, accurate estimates of the number of new variants is of particular importance, as it might help practitioners understand the site of origin of cancers, as well as the clonal origin of metastasis, and in turn be a useful tool to develop effective clinical strategies (Chakraborty et al. 2019; Huyghe et al. 2019). We make use of data from the cancer genome atlas (TCGA), and focus on the challenging scenario in which the sample size *N* is particularly small, and also small with respect to the extrapolation size *M*. Such a scenario is of interest in genomic applications, where only few samples of rare cancer might be available. We show that our BNP methodology outperforms the most popular parametric and nonparametric competitors, both classical (frequentist) and Bayesian, in terms of estimation accuracy of *U* and a refinement of *U* for rare features. In addition, with respect to the BNP approach under the stable-Beta process prior (Masoero et al. 2021), our approach provides improved coverage for the estimation. This is an empirical evidence of the effectiveness of replacing the Poisson posterior distribution with the negative Binomial posterior distribution, which allows to better exploit the sampling information.

### 1.2 Organization of the Paper

In Section 2 we show how SP priors allow to enrich the posterior distribution of *U* arising under CRM priors. In Section 3 we introduce and investigate the SB-SP prior in the context of the unseen-features problem: (i) we characterize the SB-SP prior in the class of SP priors, providing its predictive distribution; (ii) we apply the SB-SP prior to the unseen-features problem, providing the posterior distribution of *U* and a BNP estimator. Section 4 contains illustrations of our method. In Section 5 we discuss our approach, a multivariate extension of it, and future research directions. Proofs and additional experiments are in the Appendix, supplementary materials.

## 2 Scaled Process Priors for Feature Sampling Problems

For a measurable space of features W, we assume N≥1 observable individuals to be modeled as a random sample $Z_{1:N}$ from the {0, 1}-valued stochastic process $Z(w)=\sum_{i\geq 1}A_{i}\delta_{w_{i}}(w),\;w\in \text{W}$, where ${\left(w_{i}\right)}_{i\geq 1}$ are features in W and ${\left(A_{i}\right)}_{i\geq 1}$ are independent Bernoulli random variables with unknown parameters ${\left(p_{i}\right)}_{i\geq 1}$, *p_i_* being the probability that an individual has feature *w_i_*, for i≥1. That is, *Z* is a Bernoulli process with parameter $\zeta =\sum_{i\geq 1}p_{i}\delta_{w_{i}}$, denoted as BeP(ζ). BNP inference for feature sampling problems relies on the specification of a prior distribution on the discrete measure *ζ*, leading to the BNP-Bernoulli model,

(1) $$\begin{array}{c} Z_{n}\;|\;\zeta \;\overset{\text{iid}}{∼}\;\text{BeP}(\zeta )\;\;n=1,\ldots ,N,\\ \zeta \;∼\;\text{Z}, \end{array}$$

namely *ζ* is a discrete random measure on W whose law Z takes on the interpretation of a prior distribution for the unknown feature’s composition of the population. By de Finetti’s theorem, the random variables *Z_n_*’s in (1) are exchangeable with directing measure Z (Aldous 1983). In this section, we show how SP priors for *ζ* (James, Orbanz, and Teh 2015) allow to enrich the posterior distribution of the number of unseen features arising under CRM priors.

### 2.1 CRM Priors for Bernoulli Processes

CRMs provide a standard tool to define nonparametric prior distributions on the parameter *ζ* of the Bernoulli process *Z*. Consider a homogeneous CRM *μ*_0_ on W, that is, $\mu_{0}=\sum_{i\geq 1}\rho_{i}\delta_{W_{i}}$, where the *ρ_i_*’s are (0, 1)-valued random atoms such that $\sum_{i\geq 1}\rho_{i}<+\infty$, while the *W_i_*’s are iid W-valued random locations independent of the *ρ_i_*’s. The law of *μ*_0_ is characterized, through Lévy-Khintchine formula, by the Lévy intensity measure $\nu_{0}(\text{d}s,\text{d}w)=\lambda_{0}(s)\text{d}sP(\text{d}w)$ on (0,1)×W, where: (i) *λ*_0_ is a measure on (0, 1), which controls the distribution of the *ρ_i_*’s, and such that $\int_{\left(0,1\right)}\text{min}\{s,1\}\lambda_{0}(s)\text{d}s<+\infty$; ii) *P* is a nonatomic measure on W, which controls the distribution of the *W_i_*’s. For short, $\mu_{0}∼\text{CRM}(\nu_{0})$. See Appendix S1 for an account on CRMs (Kingman 1992, chap. 8). Note that, since *P* is nonatomic, the random atoms *W_i_*’s are almost surely distinct, that is to say the different features cannot coincide almost surely. The law of *μ*_0_ provides a natural prior distribution for the parameter *ζ* of the Bernoulli process. The Beta and the stable-Beta processes are popular examples of $\mu_{0}∼\text{CRM}(\nu_{0})$, for suitable specifications of *ν*_0_. A comprehensive posterior analysis of CRM priors is presented in James (2017). In the next proposition, we recall the predictive distribution of CRM priors (James 2017, Proposition 3.2).

Proposition 1. Let $Z_{1:N}$ be a random sample from (1) with $\zeta ∼\text{CRM}(\nu_{0})$. If $Z_{1:N}$ displays $K_{N}=k$ distinct features $\left\{W_{1}^{*},\ldots ,W_{K_{N}}^{*}\right\}$, each feature $W_{i}^{*}$ appearing exactly $M_{N,i}=m_{i}$ times, then the conditional distribution of $Z_{N+1}$, given $Z_{1:N}$, coincides with the distribution of

(2) $$Z_{N+1}\;|\;Z_{1:N}\overset{d}{=}Z_{N+1}^{\prime }+\sum_{i=1}^{K_{N}}A_{N+1,i}\delta_{W_{i}^{*}},$$

where: (i) $Z_{N+1}^{\prime }\;|\;\mu_{0}^{\prime }=\sum_{i\geq 1}A_{N+1,i}^{\prime }\delta_{W_{i}^{\prime }}∼\text{BeP}(\mu_{0}^{\prime })$ and $\mu_{0}^{\prime }∼\text{CRM}(\nu_{0}^{\prime })$, with $\nu_{0}^{\prime }(\text{d}s,\text{d}w)={\left(1-s\right)}^{N}\lambda_{0}(s)\text{d}sP(\text{d}w)$; (ii) the $A_{N+1,i}$’s are independent Bernoulli random variables with parameters *J_i_*’s, such that *J_i_* is distributed according to the density function $f_{J_{i}}(s)\propto {\left(1-s\right)}^{N-m_{i}}s^{m_{i}}\lambda_{0}(s)$ for i≥1.

According to (2), $Z_{N+1}$ displays “new” features $W_{i}^{\prime }$’s, that is, features not appearing in the initial sample $Z_{1:N}$, and “old” features $W_{i}^{*}$’s, that is, features appeared in the initial sample $Z_{1:N}$. The posterior distribution of statistics of “new” features is determined by the law of $Z_{N+1}^{\prime }$, which depends on $Z_{1:N}$ only through the sample size *N*; the posterior distribution of statistics of “old” features is determined by the law of $\sum_{1\leq i\leq K_{N}}A_{N+1,i}\delta_{W_{i}^{*}}$, which depends on $Z_{1:N}$ through the sample size *N*, the number *K_N_* of distinct features and their frequencies $\left(M_{N,1},\ldots ,M_{N,K_{N}}\right)$. As a corollary of Proposition 1, the posterior distribution of the number of “new” features in $\left(Z_{N+1},\ldots ,Z_{N+M}\right)$, given $Z_{1:N}$ and fixed prior’s parameters, is a Poisson distribution that depends on $Z_{1:N}$ only through *N* (Masoero et al. 2021). Such a posterior structure is peculiar to CRM priors, being inherited by the Poisson process formulation of CRMs (Kingman 1992). That is, despite the broadness of the class of CRM priors, all CRM priors lead to the same Poisson posterior structure for the number of unseen features, which thus makes them not a flexible prior model for the unseen-features problem. While the Poisson posterior distribution may be appealing in principle, making the posterior inferences analytically tractable and of easy interpretability, its independence from $Z_{1:N}$ makes the BNP approach under CRM priors a questionable oversimplification, with posterior inferences being completely determined by the estimation of unknown prior’s parameters.

Remark 1. For the sake of mathematical convenience, and in agreement with the work of James (2017), in the sequel we maintain the random measure formulation for both the prior model *μ*_0_ and the Bernoulli processes *Z_n_*. However, we point out that each *Z_n_* is equivalently characterized by means of the Bernoulli variables ${\left(A_{n,i}\right)}_{i\geq 1}$ and the random features ${\left(W_{i}\right)}_{i\geq 1}$. In other terms, there exits a one-to-one correspondence between *Z_n_* and the sequence of points ${\left\{(A_{n,i},W_{i})\right\}}_{i\geq 1}$. Finally, note that, although the values of features’ labels *W_i_* are immaterial, the features *W_i_*’s are assumed to be random. This is in line with the BNP literature on species sampling models, where the species’ labels are assumed to be random (Pitman 1996).

### 2.2 SP Priors for Bernoulli Processes

Consider a homogeneous CRM $\mu =\sum_{i\geq 1}\tau_{i}\delta_{W_{i}}$ on W, where the *τ_i_*’s are nonnegative and such that $\sum_{i\geq 1}\tau_{i}<+\infty$, and the *W_i_*’s are iid and independent of the *τ_i_*’s. We denote by ν(ds,dw)=λ(s)dsP(dw) on ${\text{R}}_{+}\times \text{W}$, with $\int_{{\text{R}}_{+}}\text{min}\{s,1\}\lambda (s)\text{d}s<+\infty$, the Lévy intensity measure of *μ*. Let $\Delta_{1}>\Delta_{2}>\cdots$ be the decreasingly ordered *τ_i_*’s, and consider the discrete random measure

$$\mu_{\Delta_{1}}=\sum_{i\geq 1}\frac{\Delta_{i+1}}{\Delta_{1}}\delta_{W_{i+1}},$$

such that $\Delta_{i+1}/\Delta_{1}\in (0,1)$, for i≥1, and $\sum_{i\geq 1}\Delta_{i+1}/\Delta_{1}<+\infty$. A SP on W is defined from $\mu_{\Delta_{1}}$ as follows. Let $F_{\Delta_{1}}(\text{d}a)=\text{exp}\left\{-\int_{a}^{\infty }\lambda (s)\text{d}s\right\}\lambda (a)\text{d}a$ be the distribution of $\Delta_{1}$ (Ferguson and Klass 1972, p. 1636), and let *G_a_* be the conditional distribution of ${\left(\Delta_{i+1}/\Delta_{1}\right)}_{i\geq 1}$ given $\Delta_{1}=a$. Moreover, let $\Delta_{1,h}$ denote a random variable whose distribution has a density function $f_{\Delta_{1,h}}(a)=h(a)f_{\Delta_{1}}(a)$, where *h* is a nonnegative function and $f_{\Delta_{1}}$ is the density function of $F_{\Delta_{1}}$. If ${\left(\rho_{i}\right)}_{i\geq 1}$ are (0, 1)-valued random variables with distribution $G_{\Delta_{1,h}}$ then

(3) $$\mu_{\Delta_{1,h}}=\sum_{i\geq 1}\rho_{i}\delta_{W_{i+1}}.$$

is a SP. For short, $\mu_{\Delta_{1,h}}∼\text{SP}(\nu ,h)$. The law of $\mu_{\Delta_{1,h}}$ is a prior distribution for the parameter *ζ* of the Bernoulli process. The next proposition characterizes the predictive distribution of SP priors. See also (James, Orbanz, and Teh 2015, Proposition 2.2) for a posterior analysis of SP priors.

Proposition 2. Let $Z_{1:N}$ be a random sample from (1) with ζ∼SP(ν,h). If $Z_{1:N}$ displays $K_{N}=k$ distinct features $\left\{W_{1}^{*},\ldots ,W_{K_{N}}^{*}\right\}$, each feature $W_{i}^{*}$ appearing exactly $M_{N,i}=m_{i}$ times, then the conditional distribution of $\Delta_{1,h}$, given $Z_{1:N}$, has a density function of the form

(4) $$g_{\Delta_{1,h}\;|\;Z_{1:N}}(a)\propto \frac{\prod_{i=1}^{k}\int_{0}^{1}s^{m_{i}}{\left(1-s\right)}^{N-m_{i}}a\lambda (as)\text{d}s}{\text{exp}\left\{\sum_{n=1}^{N}\int_{0}^{1}s{\left(1-s\right)}^{n-1}a\lambda (as)\text{d}s\right\}}f_{\Delta_{1,h}}(a).$$

Moreover, the conditional distribution of $Z_{N+1}$, given $\left(\Delta_{1,h},Z_{1:N}\right)$, coincides with the distribution of

(5) $$Z_{N+1}\;|\;(\Delta_{1,h},Z_{1:N})\overset{d}{=}Z_{N+1}^{\prime }+\sum_{i=1}^{K_{N}}A_{N+1,i}\delta_{W_{i}^{*}},$$

where as (i) $Z_{N+1}^{\prime }\;|\;\mu_{\Delta_{1,h}}^{\prime }=\sum_{i\geq 1}A_{N+1,i}^{\prime }\delta_{W_{i}^{\prime }}∼\text{BeP}(\mu_{\Delta_{1,h}}^{\prime })$ and $\mu_{\Delta_{1,h}}^{\prime }\;|\;\Delta_{1,h}∼\text{CRM}(\nu_{\Delta_{1,h}}^{\prime })$, with $\nu_{\Delta_{1,h}}^{\prime }(\text{d}s,\text{ d}w)={\left(1-s\right)}^{N}\Delta_{1,h}\lambda (s\Delta_{1,h})1_{\left(0,1\right)}(s)\text{d}sP(\text{d}w)$; (ii) the $A_{N+1,i}$’s are independent Bernoulli random variables with parameters *J_i_*’s, respectively, such that $J_{i}\;|\;\Delta_{1,h}$ is distributed according to the density function $f_{J_{i}\;|\;\Delta_{1,h}}(s)\propto {\left(1-s\right)}^{N-m_{i}}s^{m_{i}}\Delta_{1,h}\lambda (\Delta_{1,h}s)1_{\left(0,1\right)}(s)\text{d}s$ for i≥1.

See Appendix S2 for the proof of Proposition 2. The marginalization of (5) with respect to (4) leads to the predictive distribution of SP priors: (i) $Z_{N+1}$ displays “new” features $W_{i}^{\prime }$’s, and the posterior distribution of statistics of “new” features, given $Z_{1:N}$, is determined by the law of $\left(\Delta_{1,h},Z_{N+1}^{\prime }\right)$; (ii) $Z_{N+1}$ displays “old” features $W_{i}^{*}$’s, and the posterior distribution of statistics of “old” features, given $Z_{N+1}$, is determined by the law of $\left(\Delta_{1,h},\sum_{1\leq i\leq K_{N}}A_{N+1,i}\delta_{W_{i}^{*}}\right)$. Because of (4) and (5), the law of $\left(\Delta_{1,h},Z_{N+1}^{\prime }\right)$ may include the whole sampling information, depending on the specification of *ν* and *h*, and hence the posterior distribution of statistics of “new” features, given $Z_{1:N}$, also includes such an information. As a corollary of Proposition 2, the posterior distribution of the number of unseen features, given $Z_{1:N}$ and fixed prior’s parameters, is a mixture of Poisson distributions that may include the whole sampling information; in particular, the amount of sampling information in the posterior distribution is uniquely determined by the mixing distribution, namely by the conditional distribution of $\Delta_{1,h}$, given $Z_{1:N}$. SP priors thus allow to enrich the Poisson posterior structure arising from CRM priors, in terms of both a more flexible distribution and the inclusion of more sampling information than the sole sample size *N*, though they may lead to unwieldy posterior inferences due to the marginalization with respect to (4).

The use of the sampling information in the predictive structure of SPs somehow resembles that of Poisson-Kingman (PK) models (Pitman 2006). PK models form a broad class of nonparametric priors for species sampling problems. The DP prior is a PK model whose predictive distribution is such that: (i) the conditional probability that the (N+1)th draw is a “new” species, given *N* observable samples, depends only on the sample size; (ii) the conditional probability that the (N+1)th draw is an “old” species, given *N* observable samples, depends on the sample size, the number of distinct species and their frequencies. Such a behavior resembles that of CRM priors, that is, Proposition 1. PK models allow to include more sampling information in the probability of discovering a “new species” arising under the DP prior, which typically determines a loss of the analytical tractability of posterior inferences for the number of unseen species (Bacallado et al. 2017). Such a behavior resembles that of SP priors, that is, Proposition 2. The PYP prior is arguably the most popular PK model. It stands out for enriching the probability of discovering a “new” species arising under the DP prior, by including the sampling information on the number of distinct species, while maintaining the analytical tractability and interpretability of the DP prior.

## 3 Stable-Beta Scaled Process (SB-SP) Priors for the Unseen-Features Problem

In Section 2 we showed how SP priors allow to enrich the Poisson posterior structure of the number of unseen features arising under CRM priors, for example the Beta and the stable-Beta process priors. While this is an appealing property, it may lead to a lack of analytical tractability and interpretability of posterior inferences, thus, making SP priors not of practical interest in applications. In this section, we introduce and investigate a peculiar SP prior, which is referred to as the SB-SP prior, and we show that: (i) it leads to a negative Binomial posterior distribution for the number of unseen features, which generalizes the Poisson distribution while maintaining its analytical tractability and interpretability; (ii) it leads to a posterior distribution for the number of unseen features, which depends on the sampling through the sample size and the number of distinct features. The SB-SP prior thus provides a sensible tradeoff between the enrichment of the Poisson posterior structure of the number of unseen features arising under CRM priors and the analytical tractability and interpretability of posterior inferences. In particular, we characterize the SB-SP prior as the sole SP prior for which the posterior distribution of the number of unseen features depends on the observable sample only through the sample size and the number of distinct features. The SB-SP may thus be considered as a natural counterpart of the PYP for the unseen-feature problem.

### 3.1 SB-SP Priors for Bernoulli Processes

Stable scaled processes (S-SP) (James, Orbanz, and Teh 2015) form a subclass of SPs, and hence their definition follows from Section 2. In particular, for any σ∈(0,1), let $\mu_{\sigma }$ be the *σ*-stable CRM on W (Kingman 1975), which is characterized by the Lévy intensity measure $\nu_{\sigma }(\text{d}s,\text{d}w)=\lambda_{\sigma }(s)\text{d}sP(\text{d}w)$ on ${\text{R}}_{+}\times \text{W}$, with $\int_{{\text{R}}_{+}}\text{min}\{s,1\}\lambda_{\sigma }(s)\text{d}s<+\infty$, where $\lambda_{\sigma }(s)=\sigma s^{-1-\sigma }$. We recall that the largest atom $\Delta_{1}$ of $\mu_{\sigma }$ is distributed according to the density function

(6) $$f_{\Delta_{1}}(a)=\sigma a^{-1-\sigma }\text{exp}\left\{-a^{-\sigma }\right\}.$$

That is, $\Delta_{1}=E^{-1/\sigma }$, where *E* denotes a negative exponential random variable with parameter 1. For any nonnegative function *h*, a S-SP on W is defined as the SP with law $\text{SP}(\nu_{\sigma },h)$. S-SP priors generalizes the Beta process prior, which is recovered by setting *h* to be the identity function, and then letting σ→0 (James, Orbanz, and Teh 2015). The predictive distribution of $\zeta ∼\text{SP}(\nu_{\sigma },h)$ is obtained from Proposition 2. In the next theorem, we characterize the S-SP priors as the sole SP priors for which the conditional distribution of $\Delta_{1,h}$, given $Z_{1:N}$, depends on $Z_{1:N}$ only through the sample size *N* and the number *K_N_* of distinct features in $Z_{1:N}$.

Theorem 1. Let $Z_{1:N}$ be a random sample from (1) with ζ∼SP(ν,h), and let $Z_{1:N}$ displays *K_N_* distinct features with corresponding frequencies $\left(M_{N,1},\ldots ,M_{N,K_{N}}\right)$. Moreover, let ν(ds,dw)=λ(s)dsP(dw), and let $f_{\Delta_{1,h}}$ be the density function of $\Delta_{1,h}$. If $f_{\Delta_{1,h}}>0$ on ${\text{R}}_{+}$ and the functions *λ* and $f_{\Delta_{1,h}}$ are continuously differentiable, then the conditional distribution of $\Delta_{1,h}$, given $Z_{1:N}$, depends on $Z_{1:N}$ only through *N* and *K_N_* if and only if $\nu =\nu_{\sigma }$.

See Appendix S3 for the proof of Theorem 1. We recall from Section 2 that the conditional distribution of $\Delta_{1,h}$, given $Z_{1,N}$, uniquely determines the amount of sampling information included in the posterior distribution of statistics of “new” features. Then, according to Theorem 1, S-SP priors are the sole SP priors for which the posterior distribution of the number of unseen features, given $Z_{1:N}$ and fixed prior’s parameters, depends on $Z_{1:N}$ only through *N* and *K_N_*. As a corollary of Theorem 1, the Beta process prior is the sole S-SP prior for which the posterior distribution of statistics of “new” features depends on $Z_{1:N}$ only through *N*. Analogous predictive characterizations are well-known in species sampling problems, and they are typically referred to as “sufficientness” postulates’ (Bacallado et al. 2017). In particular, the DP prior is characterized as the sole species sampling prior for which the conditional probability that the (N+1)th draw is a “new” species, given *N* observable samples, depends only on the sample size (Regazzini 1978). Moreover, the PYP prior is characterized as the sole species sampling prior for which the conditional probability that the (N+1)th draw is a “new” species, given *N* observable samples, depends only on the sample size and the number of distinct species in the sample (Zabell 2005). Theorem 1 provides a “sufficientness” postulates’ in the context of feature sampling problems.

As a noteworthy example of S-SPs, we introduce the SB-SP. The SB-SP is a S-SP obtained by a suitable specification of the nonnegative function *h*. In particular, for any c,β>0 let

(7) $$h_{c,\beta }(a)=\frac{\beta^{c+1}}{\Gamma (c+1)}a^{-c\sigma }\text{exp}\left\{-(\beta -1)a^{-\sigma }\right\},$$

where Γ(·) denotes the Gamma function. Then a SB-SP on W is defined as the SP with law $\text{SP}(\nu_{\sigma },h_{c,\beta })$. For short, we denote the law of a SB-SP by SB−SP(σ,c,β). The SB-SP prior generalizes the Beta process prior, which is recovered by setting *c* = 0 and *β* = 1, and then letting σ→0. According to the construction of SPs, the distribution of $\Delta_{1,h_{c,\beta }}$ has a density function obtained by combining (6) and (7); this is a polynomial-exponential tilting of the density function (6). In particular, $\Delta_{1,h_{c,\beta }}^{-\sigma }$ is distributed as a Gamma distribution with shape (c+1) and rate *β*. Such a straightforward distribution for $\Delta_{1,h_{c,\beta }}$ is at the core of the analytical tractability of posterior inferences under the SB-SP prior; this fact will be clear in the application of the SB-SP prior to the problem of estimating the number of unseen features. The next proposition characterizes the predictive distribution of the SB-SP prior.

Proposition 3. Let $Z_{1:N}$ be a random sample from (1) with ζ∼SB−SP(σ,c,β). If $Z_{1:N}$ displays $K_{N}=k$ distinct features $\left\{W_{1}^{*},\ldots ,W_{K_{N}}^{*}\right\}$, each feature $W_{i}^{*}$ appearing exactly $M_{N,i}=m_{i}$ times, then the conditional distribution of $\Delta_{1,h_{c,\beta }}$, given $Z_{1:N}$, has a density function of the form

(8) $$\begin{array}{c} g_{\Delta_{1,h_{c,\beta }}\;|\;Z_{1:N}}(a)\\ \;=\sigma \frac{{\left(\beta +\gamma_{0}^{\left(N\right)}\right)}^{k+c+1}}{\Gamma (k+c+1)}a^{-k\sigma -(c+1)\sigma -1}{\text{e}}^{-a^{-\sigma }(\beta +\gamma_{0}^{\left(N\right)})}, \end{array}$$

where $\gamma_{0}^{\left(N\right)}=\sigma \sum_{1\leq i\leq N}B(1-\sigma ,i)$, with B(·,·) being the (Euler) Beta function. Moreover, the conditional distribution of $Z_{N+1}$, given $\left(\Delta_{1,h_{c,\beta }},Z_{1:N}\right)$, coincides with the distribution of

(9) $$Z_{N+1}\;|\;(\Delta_{1,h_{c,\beta }},Z_{1:N})\overset{d}{=}Z_{N+1}^{\prime }+\sum_{i=1}^{K_{N}}A_{N+1,i}\delta_{W_{i}^{*}},$$

where:

- $Z_{N+1}^{\prime }\;|\;\mu_{\Delta_{1,h_{c,\beta }}}^{\prime }=\sum_{i\geq 1}A_{N+1,i}^{\prime }\delta_{W_{i}^{\prime }}∼\text{BeP}(\mu_{\Delta_{1,h_{c,\beta }}}^{\prime })$ such that $\mu_{\Delta_{1,h_{c,\beta }}}^{\prime }\;$ $\left|\;\Delta_{1,h_{c,\beta }}∼\text{CRM}(\nu_{\Delta_{1,h_{c,\beta }}}^{\prime }\right)$, with
  $$\begin{array}{c} \nu_{\Delta_{1,h_{c,\beta }}}^{\prime }(\text{d}s,\text{ d}w)\\ \;=\Delta_{1,\Delta_{1,h_{c,\beta }}}^{-\sigma }{\left(1-s\right)}^{N}\sigma s^{-1-\sigma }1_{\left(0,1\right)}(s)\text{d}sP(\text{d}w); \end{array}$$
- the $A_{N+1,i}$’s are independent Bernoulli random variables with parameters *J_i_*’s, respectively, such that each $J_{i}\;|\;\Delta_{1,h_{c,\beta }}$ is distributed according to a density function of the form
  $$\begin{array}{c} f_{J_{i}\;|\;\Delta_{1,h_{c,\beta }}}(s)\\ =\frac{1}{B(m_{i}-\sigma ,N-m_{i}+1)}s^{m_{i}-\sigma }{\left(1-s\right)}^{N-m_{i}+1}1_{\left(0,1\right)}(s). \end{array}$$

See Appendix S3 for the proof of Proposition 3. According to Equation (8), the conditional distribution of $\Delta_{1,h_{c,\beta }}$, given $Z_{1:N}$, depends on $Z_{1:N}$ only through the sample size *N* and the number *K_N_* of distinct features in $Z_{1:N}$. This agrees with Theorem 1, implying that the posterior distribution of the number of unseen features, given $Z_{1:N}$ and fixed prior’s parameters, depends on $Z_{1:N}$ only through *N* and *K_N_*. Because of (8) and (9), the posterior distribution of statistics of “new” features stands out for analytical tractability, thus, being competitive with that arising from CRMs, for example, the Beta and the stable-Beta processes. In particular, from Equation (9), the conditional distribution of $Z_{N+1}^{\prime }$, given $\left(\Delta_{1,h_{c,\beta }},Z_{1:N}\right)$ is a Poisson distribution that depends on $Z_{1:N}$ only through *N*. Then, from (8), its marginalization with respect to the conditional distribution of $\Delta_{1,h_{c,\beta }}$, given $Z_{1:N}$, leads to a negative Binomial posterior distribution. Such an appealing property arises from the peculiar form $h_{c,\beta }$ that, combined with $\nu_{\sigma }$, leads to a conjugacy property for the conditional distribution of $\Delta_{1,h_{c,\beta }}$, given $Z_{1:N}$. That is, the conditional distribution of $\Delta_{1,h_{c,\beta }}^{-\sigma }$, given $Z_{1:N}$, is a Gamma distribution with shape $\left(K_{N}+c+1\right)$ and rate $\beta +\gamma_{0}^{\left(N\right)}$, which is the distribution $\Delta_{1,h_{c,\beta }}^{-\sigma }$ with shape and rate being updated through $Z_{1:N}$. The next proposition establishes the distribution of a random sample $Z_{1:N}$ from a SB-SP prior. See Appendix S3 for details.

Proposition 4. Let $Z_{1:N}$ be a random sample from (1) with ζ∼SB−SP(σ,c,β). The probability that $Z_{1:N}$ displays a particular feature allocation of *k* distinct features with frequencies $\left(m_{1},\ldots ,m_{k}\right)$ is

(10) $$\begin{array}{c} p_{k}^{\left(N\right)}(m_{1},\ldots ,m_{k})=\frac{\frac{\sigma^{k}\beta^{c+1}}{{\left(\beta +\gamma_{0}^{\left(N\right)}\right)}^{k+c+1}}}{\frac{\Gamma (c+1)}{\Gamma (k+c+1)}}\prod_{i=1}^{k}\\ \times \frac{\Gamma (m_{i}-\sigma )\Gamma (N-m_{i}+1)}{\Gamma (N-\sigma +1)}. \end{array}$$

### 3.2 BNP Inference for the Unseen-Features Problem

Now, we apply the SB-SP prior to the unseen-features problem. For any N≥1 let $Z_{1:N}$ be an observable sample modeled as the BNP Bernoulli model (1), with ζ∼SB−SP(σ,c,β). Moreover, under the same model of the *Z_n_*’s, for any M≥1 let $\left(Z_{N+1},\ldots ,Z_{N+M}\right)$ be additional unobservable sample. Then, the unseen-feature problem calls for the estimation of

(11) $$U_{N}^{\left(M\right)}=\sum_{i\geq 1}1\left(\sum_{m=1}^{M}A_{N+m,i}>0\right)1\left(\sum_{n=1}^{N}A_{n,i}=0\right),$$

namely the number of hitherto unseen features that would be observed in $\left(Z_{N+1},\ldots ,Z_{N+M}\right)$. As generalization of the unseen-feature problem (11), for r≥1 we consider the estimation of

(12) $$U_{N}^{\left(M,r\right)}=\sum_{i\geq 1}1\left(\sum_{m=1}^{M}A_{N+m,i}=r\right)1\left(\sum_{n=1}^{N}A_{n,i}=0\right),$$

namely the number of hitherto unseen features that would be observed with prevalence *r* in $\left(Z_{N+1},\ldots ,Z_{N+M}\right)$. Of special interest is *r* = 1, which concerns rare (unique) features. The next theorem characterizes the posterior distributions of $U_{N}^{\left(M\right)}$ and $U_{N}^{\left(M,r\right)}$, given $Z_{1:N}$. We denote by NegativeBinonial(n,p) the negative Binomial distribution with parameter *n* and p∈(0,1).

Theorem 2. Let $Z_{1:N}$ be a random sample from (1) with ζ∼SB−SP(σ,c,β), and let $Z_{1:N}$ displays $K_{N}=k$ distinct features with frequencies $\left(M_{N,1},\ldots ,M_{N,K_{N}})=(m_{1},\ldots ,m_{k}\right)$. Then, the posterior distributions of $U_{N}^{\left(M\right)}$ and of $U_{N}^{\left(M,r\right)}$, given $Z_{1:N}$, coincide with the distributions of

(13) $$U_{N}^{\left(M\right)}\;|\;Z_{1:N}∼\text{NegativeBinonial}\left(K_{N}+c+1,\frac{\gamma_{N}^{\left(M\right)}}{\beta +\gamma_{0}^{\left(N+M\right)}}\right),$$

and

(14) $$\begin{array}{c} U_{N}^{\left(M,r\right)}\;|\;Z_{1:N}∼\text{NegativeBinonial}\\ \times \left(K_{N}+c+1,\frac{\rho_{N}^{\left(M,r\right)}}{\beta +\gamma_{0}^{\left(N\right)}+\rho_{N}^{\left(M,r\right)}}\right), \end{array}$$

for any index of prevalence r≥1, respectively, where $\gamma_{N}^{\left(M\right)}=\sigma \sum_{1\leq i\leq M}B(1-\sigma ,N+i)$ and where $\rho_{N}^{\left(M,r\right)}=\left(\begin{array}{c} M\\ r \end{array}\right)\sigma B(r-\sigma ,N+M-r+1)$, with B(·,·) denoting the (Euler) Beta function.

See Appendix S4 for the proof of Theorem 2. The posterior distributions (13) and (14) depend on $Z_{1:N}$ through the sample size *N* and the number *K_N_* of distinct features. This is in contrast with the corresponding posterior distributions obtained under the Beta and the stable-Beta process priors, which are Poisson distributions that depend on $Z_{1:N}$ only through *N* (Masoero et al. 2021, Proposition 1). BNP estimators of $U_{N}^{\left(M\right)}$ and $U_{N}^{\left(M,r\right)}$, with respect to a squared loss function, are obtained as the posterior expectations of (13) and (14), that is,

(15) $${\hat{U}}_{N}^{\left(M\right)}=(K_{N}+c+1)\frac{\gamma_{N}^{\left(M\right)}}{\beta +\gamma_{0}^{\left(N+M\right)}-\gamma_{N}^{\left(M\right)}}$$

and

(16) $${\hat{U}}_{N}^{\left(M,r\right)}=(K_{N}+c+1)\frac{\rho_{N}^{\left(M,r\right)}}{\beta +\gamma_{0}^{\left(N\right)}},$$

respectively. The estimators (15) and (16) are simple, linear in the sampling information and computationally efficient. In the next theorem we establish the large *M* asymptotic behavior of the posterior distributions (13) and (14), showing that the number of unseen features has a power-law growth in *M*. The same growth in *M* holds under the stable-Beta process prior (Masoero et al. 2021, Proposition 2), though the limiting distribution is degenerate.

Theorem 3. Let $Z_{1:N}$ be a random sample from (1) with ζ∼SB−SP(σ,c,β), and let $Z_{1:N}$ displays $K_{N}=k$ distinct features with frequencies $\left(M_{N,1},\ldots ,M_{N,K_{N}})=(m_{1},\ldots ,m_{k}\right)$. As M→+∞

(17) $$\frac{U_{N}^{\left(M\right)}}{M^{\sigma }}⏧Z_{1:N}\overset{\text{a}.\text{s}.}{\to }W_{N},$$

where *W_N_* is a Gamma random variable with shape $\left(K_{N}+c+1\right)$ and rate $\left(\beta +\gamma_{0}^{\left(N\right)})/\Gamma (1-\sigma \right)$, and

(18) $$\frac{U_{N}^{\left(M,r\right)}}{M^{\sigma }}⏧Z_{1:N}\overset{\text{a}.\text{s}.}{\to }W_{N,r},$$

where $W_{N,r}$ is a Gamma random variable with shape $\left(K_{N}+c+1\right)$ and rate $\Gamma (r+1)(\beta +\gamma_{0}^{\left(N\right)})/\sigma \Gamma (r-\sigma )$.

## 4 Experiments

Over the last decade, genomics has witnessed an extraordinary improvement in the data availability due to the advent of next generation sequencing technologies. Thanks to larger and richer datasets, researchers have started uncovering the role and impact of rare genetic variants in heritability and human disease (Hernandez et al. 2019; Momozawa and Mizukami 2020). The development of methods for estimating the number of new genomic variants to be observed in future studies is an active research area, as it can aid the design of effective clinical procedures in precision medicine (Ionita-Laza, Lange, and Laird 2009; Zou et al. 2016), enhance understanding of cancer biology (Chakraborty et al. 2019), and help to optimize sequencing procedures (Rashkin et al. 2017; Masoero et al. 2021). Here, we consider datasets of individual genomic sequences. Following common practice, we assume that an underlying fixed and idealized genomic sequence (the “reference”) is given. Then, each coordinate of an individual sequence reports the presence (1) or absence (0) of variation at a given locus with respect to the reference. All variants are treated equally, namely, any expression differing from the underlying reference at a given locus counts as a variant. We make use of our methodology to estimate the number of genomic loci at which variation was not observed in the original sample, and is going to be observed in (at least one of) *M* additional datapoints.

We find in our experiments that the estimates of the total number of new variants to be observed produced using the SB-SP-Bernoulli model, hereafter referred to as SSB, tend to be more accurate than other available methods in the literature. This phenomenon is particularly evident when the sample size *N* of the training set is small, and when the extrapolation size *M* is large with respect to *N*. Moreover, the SSB model is particularly effective in estimating the number of new rare variants, for example, variants appearing only once in the additional unobservable samples. Accurate estimation of rare variants is particularly important, as these are believed to be largely responsible for heritability of human disease (Rashkin et al. 2017; Chakraborty et al. 2019). To benchmark the quality of the SSB, we consider a number of competing methodologies for the feature prediction problem available in the literature: (i) Jackknife estimators (J) (Gravel 2014); (ii) a linear programming method (LP) (Zou et al. 2016) and variations of Good-Toulmin estimators (GT) (Chakraborty et al. 2019). We also compare our empirical findings to a BNP estimator obtained under the stable-Beta process prior (3BB), which has been introduced in Masoero et al. (2021). We complete our analysis with a thorough investigation on synthetic data in S6 and S7, as well as on additional real data from the gnomAD database (Karczewski et al. 2020) in S8. https://github.com/lorenzomasoero/ScaledProcesses contains code and data to replicate all our analyses.

### 4.1 Empirics and Evaluation Metrics

For the SSB method to be useful, we need to estimate the underlying, unknown, parameters of the SB-SP prior. To learn these prior’s parameters, we here adopt an empirical Bayes procedure, which consists in maximizing the marginal distribution (10). In particular, we maximize numerically Equation (10) with respect to the parameters β>0, *c* > 0 and σ∈(0,1) of the SB-SP prior, and use the resulting values to produce our estimators. That is, we let

$$(\hat{\beta },\hat{c},\hat{\sigma })=\text{arg}\underset{\left(\beta ,c,\sigma \right)}{\text{max}}\left\{p_{k}^{\left(N\right)}(m_{1},\ldots ,m_{k})\right\},$$

and plug these values in the BNP estimator (13) and (14). The resulting values provide our BNP estimates of the number $U_{N}^{\left(M\right)}$ of new variants and the number $U_{N}^{\left(M,r\right)}$ of new variants with prevalence *r*.

To assess the accuracy of our estimates, we consider the percent deviation of the estimate from the truth to be the achieved accuracy. That is, the accuracy of the estimator ${\hat{U}}_{N}^{\left(M\right)}$ is defined as

(19) $$v_{N}^{\left(M\right)}:=1-\text{min}\left\{\frac{\left|U_{N}^{\left(M\right)}-{\hat{U}}_{N}^{\left(M\right)}\right|}{U_{N}^{\left(M\right)}},1\right\}.$$

In particular, the accuracy $v_{N}^{\left(M\right)}$ equals 1 when the estimate is perfect (no error is incurred), and decreases to 0 as the estimate deviates from the truth. The min⁡ operator in (19) ensures that $v_{N}^{\left(M\right)}$ lies in [0,1]: we let the accuracy to be equal to 0 whenever there is a severe overestimation, and the percentage estimation error exceeds 100%, that is, when ${\hat{U}}_{N}^{\left(M\right)}\geq 2\times U_{N}^{\left(M\right)}$. The SSB, 3BB and LP methods also offer an estimate for the number of new features observed with a given prevalence *r*. We let $v_{N}^{\left(M,r\right)}$ be the accuracy metric, where we replace in (19) $U_{N}^{\left(M\right)}$ with $U_{N}^{\left(M,r\right)}$, the number of new features observed with prevalence *r*, and ${\hat{U}}_{N}^{\left(M\right)}$ with ${\hat{U}}_{N}^{\left(M,r\right)}$.

### 4.2 Estimating the Number of New Variants in Cancer Genomics

Following the empirical study of Chakraborty et al. (2019), we make use of data from the Cancer Genome Atlas (TCGA), the largest publicly available cancer genomics dataset, containing somatic mutations from 10,295 patients and spanning 33 different cancer types. We partition the samples into 33 smaller datasets according to cancer-type annotation of each patient. See Chakraborty et al. (2019) and (Masoero et al. 2021, Appendix F) for details on the data and the experimental setup. For each cancer type, we retain a small fraction of the data for purposes of training, and consider the task of estimating the number of new variants that will be observed in a follow-up sample given a pilot sample. We validate our estimates by comparing the estimate ${\hat{U}}_{N}^{\left(M\right)}$ of the number of distinct variants to the true value, obtained by extrapolating to the remaining data. To assess the variability and error in our estimates, we repeat for every cancer type the experiment on *S* = 1000 subsets of the data, each obtained by randomly subsampling without replacement from the full sample.

**Fig. 1:**
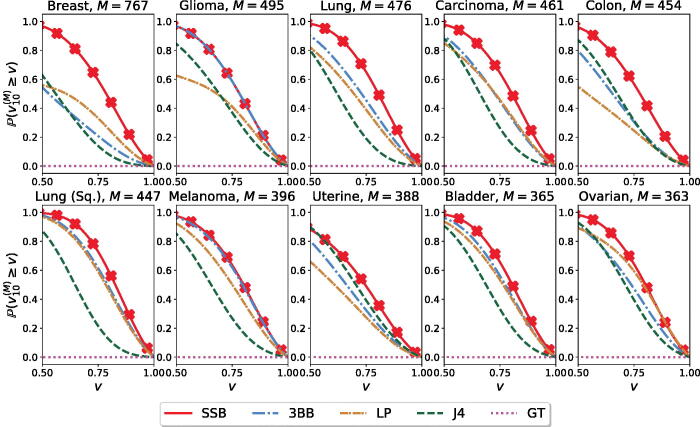
Estimation accuracy $v_{N}^{\left(M\right)}$ for the number of new genomic variants ${\hat{U}}_{10}^{\left(M\right)}$. For each method and each cancer type, we retain *N* = 10 random samples and use them to estimate up to *M* total observations, where *N* + *M* is the size of the original sample.

We find that the SSB and 3BB methods perform particularly well when the training sample size *N* is small compared to the extrapolation sample size *M*. This setting is relevant in the context of cancer genomics, as scientists are interested in understanding the “unexploited potential” of the genetic information, especially for rare cancer subtypes (Chakraborty et al. 2019; Huyghe et al. 2019). To compare and quantify the performance of the available methodologies in this setting, we report in Figure 1 the distribution of the estimation accuracy when retaining only *N* = 10 samples for training and extrapolating to the largest possible sample size *M* for which we can compute the accuracy metric (Equation (19)). We report results for the 10 cancer types with the largest number of samples in the original dataset. For each cancer type and for each method, the distribution of the estimation accuracy is obtained by considering its performance across the *S* = 1000 replicates. Across all cancer types, the estimates obtained from the SSB method achieve higher accuracy.

**Fig. 2:**
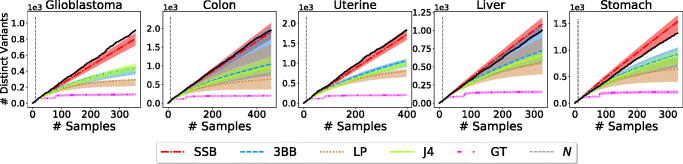
Estimation of the number of new genomic variants ${\hat{U}}_{N}^{\left(i\right)}$, for i=1,…,M. For each method and cancer type, we retain *N* = 10 random samples and use them to estimate up to the largest possible size. We fit each model on the full sample, as well as *N* = 10 additional times by iteratively leaving one datapoint out from the training sample. The solid black line is the true number of features that would have been observed (vertical axis) for any extrapolation size *N* + *M* (horizontal axis), for a fixed ordering of the data. Shaded regions report the prediction range obtained from the estimates from the leave-one-out fits.

We show in Figure 2 the behavior of ${\hat{U}}_{N}^{\left(i\right)}$ for five different cancer types as i=1,…,M. Again, we let *N* = 10, and *M* be the largest possible extrapolation value, as dictated by the dataset size. We report the estimates obtained from a fixed sample of size *N* = 10, as well as the variability around such estimates obtained by refitting each model, iteratively leaving one datapoint out from the sample. In this setting, the SSB method outperforms competing methods in terms of estimation accuracy. Moreover, the variability in the estimates arising from refitting the model on subsets of the data provides a useful measure of uncertainty in such estimation.

### 4.3 Estimating the Number of New Rare Variants in Cancer Genomics

In recent years, the cancer genomics research community has become increasingly interested in studying and understanding the role of extremely rare variants, such as singletons, that is, observed in only one patient. Evidence suggests that rare deleterious variants can have far stronger effect sizes than common variants (Rasnic, Linial, and Linial 2020) and can play an important role in the development of cancer. For example, in breast cancer, it is well accepted that the risk of a variant is inversely proportional with respect to its prevalence: the rarer the variant, the higher the risk (Wendt and Margolin 2019). Therefore, effective identification and discovery of rare variants is an active, is an ongoing research area (Lawrenson et al. 2016; Lee et al. 2019). This phenomenon is not limited to breast cancer, but is progressively being studied across different cancer types. See, for example, the recent works on ovarian (Phelan et al. 2017), skin (Goldstein et al. 2017), prostate (Nguyen-Dumont et al. 2020) and lung (Liu et al. 2021) cancers and references therein. In downstream analysis, these estimates could be useful for planning and designing future experiments, for example, informing scientists on the number of new samples to be collected in order to observe a target number of new variants, or for power analysis considerations in rare variants association tests (Rashkin et al. 2017).

**Fig. 3:**
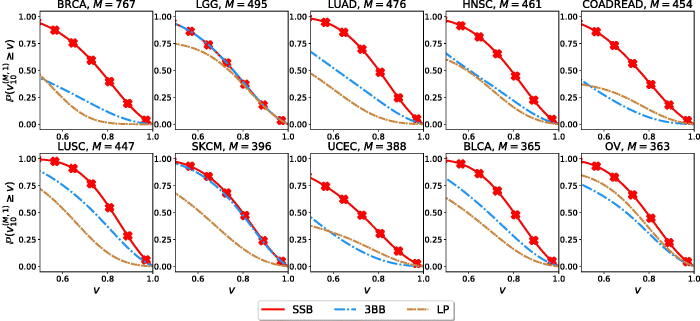
Estimation accuracy $v_{N}^{\left(M,1\right)}$ for new variants appearing with prevalence one in future unobservable samples for different cancer types. For each method and each cancer, we retain *N* = 10 random samples and use them to estimate up to the largest possible size.

**Fig. 4:**
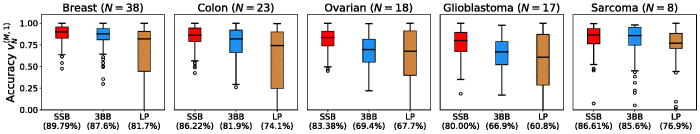
Estimation accuracy $v_{N}^{\left(M,1\right)}$ for new variants appearing with prevalence one in future samples. For each method and different cancer types, we retain a random sample of size N=5% of the available dataset, and use it to estimate up to the largest possible size.

The BNP framework considered here allows us to estimate the number of new rare variants to be discovered (Figure 3). While Zou et al. (2016) did not consider the problem of estimating rare variants, it is straightforward to obtain an estimate for this quantity from their framework. Indeed, for every prevalence x∈[0,1], the LP estimates the histogram *h*(*x*), which counts the number of variants appearing with prevalence *x* in the population, and the number of variants appearing with prevalence *r* follows from the binomial sampling model assumption, namely ${\hat{U}}_{N}^{\left(M,r\right)}=\sum_{x}h(x)\left\{\left(\begin{array}{c} N+M\\ r \end{array}\right)x^{r}{\left(1-x\right)}^{N+M-r}-\left(\begin{array}{c} N\\ r \end{array}\right)x^{r}{\left(1-x\right)}^{N-r}\right\}$. We show in Figure 4 that the SSB method provides better estimates than the 3BB and LP methods.

### 4.4 Coverage and Calibrated Uncertainties

One of the benefits of the BNP approach is that it automatically yields a notion of variability of the estimate of *U* via posterior credible intervals. We here check whether these intervals produce a useful notion of uncertainty, by investigating their calibration. For α∈(0,1), we say that a 100×α% credible interval is calibrated if it contains the true value of interest, arising from hypothetical repeated draws, 100×α% of the times. We here assess the calibration of a 100×α% credible interval for $U_{N}^{\left(M\right)}$ conditionally given $Z_{1:N}$ as follows. Let *S* be a large number (*S* = 1000 in our experiments). For each s=1,…,S, we retain a random subset of the data of size *N*, and estimate the corresponding parameters $\hat{\beta },\hat{c},\hat{\sigma }$ as discussed in Section 4.1. Then, we let ${\hat{W}}_{N,s,\text{low}}^{\left(M\right)}(\alpha ),{\hat{W}}_{N,s,\text{hi}}^{\left(M\right)}(\alpha )$ be the endpoints of a 100×α% credible interval for the distribution of the number of new features, as given by Equation (13), centered around the posterior predictive mean. We compute coverage calibration via

$$w_{N}^{\left(M\right)}(\alpha )=\frac{1}{S}\sum_{s=1}^{S}1\left\{{\hat{W}}_{N,s,\text{low}}^{\left(M\right)}(\alpha )\leq K_{N+M}\leq {\hat{W}}_{N,s,\text{hi}}^{\left(M\right)}(\alpha )\right\}.$$

**Fig. 5:**
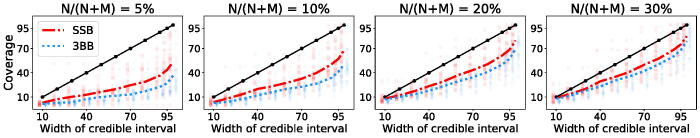
Coverage calibration of BNP estimators for number of new variants in future samples across all cancer types in TCGA. Different subplots refer to different ratios of the training *N* with respect to the extrapolation *M*. For each cancer, we retain a training sample of size N∈{5%,10%,20%,30%} of the total available dataset, and extrapolate up to the largest available *M*. Colored lines report the average coverage $w_{N}^{\left(M\right)}(\alpha )$ across all cancer types (*y*-axis) as a function of *α* (*x*-axis). Faded dots refer to coverage for individual cancer types.

This is the fraction of the *S* experiments in which the true value was contained by a 100×α% credible interval. The closer $w_{N}^{\left(M\right)}(\alpha )$ to *α*, the better calibrated the credible intervals. We compute the same quantity for the 3BB method using the results in Masoero et al. (2021). Although still not perfect, we find that the posterior predictive intervals obtained from the SSB method are better calibrated than the ones under the 3BB method (see Figure 5).

## 5 Discussion

Masoero et al. (2021) first applied CRM priors to the unseen-features problem, showing that: (i) despite the broadness of the class of CRM priors, all CRM priors lead to the same Poisson posterior structure for the number of unseen features, which thus makes them not a flexible prior model for the unseen-features problem; (ii) while the Poisson posterior distribution may be appealing in principle, making the posterior inferences analytically tractable and of easy interpretability, its independence from $Z_{1:N}$ makes the BNP approach a questionable oversimplification, with posterior inferences being completely determined by the estimation of unknown prior’s parameters. In this article, we introduced the SB-SP prior, and showed that: (i) it enriches the posterior distribution of the number of unseen features arising under CRM priors, which results in a negative Binomial distribution whose parameters depend on the sample size and the number of distinct features; (ii) it maintains the same analytical tractability and interpretability as CRM priors, which results in BNP estimators that are simple, linear in the sampling information and computationally efficient. The effectiveness of the SB-SP prior is showcased through an empirical analysis on synthetic and real data. Under the SB-SP prior, we found that estimates of the unseen number of features are accurate, and they outperform the most popular competitors in the challenging scenario where the sample size *N* is particularly small, and also small with respect to the extrapolation size *M*.

Our approach admits an extension to the multiple-feature setting, which takes into account of the many forms of variation, for example, single nucleotide changes, tandem repeats, insertions and deletions, copy number variations (Zou et al. 2016). We briefly describe the multiple-feature setting, and defer to Appendix S5 for details. It is assumed that a feature *w_i_* comes with a characteristic, that is, the form of variation, chosen among *q* > 1 characteristics. For N≥1, the observable sample $Z_{1:N}=(Z_{1},\ldots ,Z_{N})$ is modeled as a ${\left\{0,1\right\}}^{q}$-valued stochastic process $Z=\sum_{i\geq 1}A_{i}\delta_{w_{i}}$, where $A_{i}:=(A_{i,1},\ldots ,A_{i,q})$ is a Multinomial random variable with parameter $p_{i}=(p_{i,1},\ldots ,p_{i,q})$ such that $|p_{i}|=\sum_{1\leq j\leq q}p_{i,j}<1$, and the $A_{i}$’s are iid. That is, for any i≥1 all the $A_{i,j}$’s are equal to 0 with probability $\left(1-|p_{i}|\right)$, that is, *w_i_* does not display variation, or only one $A_{i,j}$’s is equal to 1 with probability $p_{i,j}$, that is, *w_i_* displays variation with characteristic *j*. ***Z*** is a multivariate Bernoulli process with parameter $\zeta =\sum_{i\geq 1}p_{i}\delta_{w_{i}}$. The stable-Beta-Dirichlet process prior for ζ is a multivariate generalization of the stable-Beta process prior (James 2017), and it leads to a Poisson posterior distribution for the number of unseen features, given $Z_{1:N}$, which depends on $Z_{1:N}$ only through *N*. In Appendix S5 we introduce a scaled version of the stable-Beta-Dirichlet process, and show that it leads to a negative Binomial posterior distribution for the number of unseen features, which depends on $Z_{1:N}$ through *N* and the number of distinct features in $Z_{1:N}$.

SP priors have been introduced in James, Orbanz, and Teh (2015) and, to the best of our knowledge, since then no other works have further investigated such a class of priors. To date, the peculiar predictive properties of SP priors appear to be unknown in the BNP literature. Our work on the unseen-features problem is the first to highlight the great potential of SP priors in BNPs, showing that they provide a critical tool for enriching the predictive structure of the popular CRM priors (James 2017; Broderick, Wilson, and Jordan 2018). We believe that SPs may be of interest beyond the unseen-features problem, and more generally beyond the broad class of feature sampling problems. CRM priors, and in particular the Beta and stable-Beta process priors, have been widely used in several contexts, with a broad range of applications in topic modeling, analysis of social networks, binary matrix factorization for dyadic data, analysis of choice behavior arising from psychology and marketing surveys, graphical models, and analysis of similarity judgment matrices. See Griffiths and Ghahramani (2011) and references therein for details. In all these contexts, SP priors may be more effective than CRM priors, as they allow to better exploit the sampling information in posterior inferences.

Among applications of SP priors beyond features sampling problems, it is worth mentioning the use of SP priors as hierarchical (or latent) priors in models of unsupervised learning (Griffiths and Ghahramani 2011, sec. 5), the most popular being Gaussian latent feature modeling. Differently from features sampling problems, where the values of features’ labels *W_i_*s are immaterial, in Gaussian latent feature modeling the values the *W_i_*’s become material. That is, under the Gaussian latent feature model with a SP prior, observations are assumed to modeled as a multivariate Gaussian distribution, whose mean depends on latent features that are modeled with a SP prior, thus, making the values of features’ labels *W_i_*’s of critical importance for the analysis. Bayesian factor analysis (Knowles and Ghahramani 2011) provides another context where SP priors may be usefully applied as hierarchical priors. Within the context of factor analysis, we also mention the work of Ayed and Caron (2021) with applications to network analysis. There, the authors exploit CRM priors to recover the latent community structure in a network between individuals, and the features’ labels describe the level of affiliation of a certain individual to a latent community. In such a context, we believe that SP priors may be used in place of CRM priors, with the advantage of introducing richer predictive structure. In this respect, our work paves the way to promising directions of future research, in terms of both methods and applications.

## Supplementary Material

Supplemental Material

Supplemental Material

## Supplementary Materials

All proofs of our statements, as well as additional experiments can be found in the Appendix. Code to replicate all our analyses is available at https://github.com/lorenzomasoero/ScaledProcesses/.
