# Supplementary material for: Scaled Process Priors for Bayesian Nonparametric Estimation of the Unseen Genetic Variation
Source: J Am Stat Assoc. 2022 Sep 29;119(545):320–31. doi: 10.1080/01621459.2022.2115918 (PMC11073059; doi:10.1080/01621459.2022.2115918)
Supplement: Supplemental Material [file UASA_A_2115918_SM5205.zip › supplementary_files/Supplementary_material.pdf]

Supplementary Material for "Scaled process priors for Bayesian nonparametric estimation of the unseen genetic variation"

Federico Camerlenghi*
Department of Economics, Management and Statistics
University of Milano - Bicocca, Milan, Italy
and
Stefano Favaro
Department of Economics and Statistics
University of Torino and Collegio Carlo Alberto, Torino, Italy
and
Lorenzo Masoero
Department of Electrical Engineering and Computer Science, CSAIL
Massachusetts Institute of Technology
Cambridge, Massachusetts, USA
and
Tamara Broderick
Department of Electrical Engineering and Computer Science, CSAIL
Massachusetts Institute of Technology
Cambridge, Massachusetts, USA

July 16, 2022

*Also affiliated to Collegio Carlo Alberto, Piazza V. Arbarello 8, Torino and BIDSA, Bocconi University, Milano, Italy.

S1 A brief account on completely random measures

In this section we provide a short account on completely random measures (CRMs). For a more exhaustive treatment refer to Daley and Vere-Jones [2008], Kingman [1992]. Let us denote by $\mathbb{W}$ a Polish space equipped with its Borel σ-field $\mathcal{W}$, and we also indicate by $\mathcal{B}_{\mathbb{R}_+}$ the Borel σ-field of the positive real line $\mathbb{R}_+$. Denote by $\mathsf{M}_{\mathbb{W}}$ the space of all bounded and finite measures on $(\mathbb{W}, \mathcal{W})$, in other words $\mu \in \mathsf{M}_{\mathbb{W}}$ iff $\mu(A) < +\infty$ for any bounded set $A \in \mathcal{W}$. The space $\mathsf{M}_{\mathbb{W}}$ is usually assumed to be equipped with a proper Borel σ-algebra, which is induced by the so called *weak-hash convergence* and denoted here as $\mathcal{M}_{\mathbb{W}}$ (see Daley and Vere-Jones [2008] for details).

Definition S1. *A Completely Random Measure (CRM) μ on $(\mathbb{W}, \mathcal{W})$ is a random element defined on a suitable probability space and taking values in $(\mathsf{M}_{\mathbb{W}}, \mathcal{M}_{\mathbb{W}})$ such that the random variables $\mu(A_1), \dots, \mu(A_n)$ are independent for any choice of bounded and disjoint sets $A_1, \dots, A_n \in \mathcal{W}$ and for any $n \geq 1$.*

Kingman [1967] proved that a CRM may be decomposed as the sum of three main components: i) a deterministic drift u, namely a deterministic measure on $(\mathbb{W}, \mathcal{W})$; ii) a part with random jumps $(\tau_i)_{i\geq 1}$ at random locations $(W_i)_{i\geq 1}$, denoted here as $\mu_c = \sum_{i\geq 1} \tau_i \delta_{W_i}$; iii) a component with random jumps $(\eta_i)_{i\geq 1}$ at fixed locations $w_1, w_2, \dots \in \mathbb{W}$. That is to say

$$\mu(\,\cdot\,) = u(\,\cdot\,) + \mu_c(\,\cdot\,) + \sum_{i\geq 1} \eta_i \delta_{w_i}(\,\cdot\,). \tag{S1}$$

See Daley and Vere-Jones [2008] for a proof.

Following standard practice in the nonparametric literature, in this paper we deal with CRMs without deterministic drift and without fixed atoms, namely we assume that $\mu \equiv \mu_c$. In this case $\mu = \mu_c$ is characterized through the Lévy-Khintchine representation of its

Laplace functional:

$$\mathbb{E}\left[e^{-\int_{\mathbb{W}} f(w)\mu_c(\mathrm{d}w)}\right] = \exp\left\{-\int_{\mathbb{R}_+\times\mathbb{W}} (1-e^{-sf(w)})\nu(\mathrm{d}s,\mathrm{d}w)\right\}, \tag{S2}$$

for any measurable function $f : \mathbb{W} \to \mathbb{R}_+$, where ν is a measure on $\mathbb{R}_+ \times \mathbb{W}$ and it is referred to as the Lévy intensity of the CRM μ_c. The measure ν is also required to satisfy the following conditions

$$\nu(\mathbb{R}_+ \times \{w\}) = 0 \quad \forall w \in \mathbb{W}, \quad \text{and} \int_{\mathbb{R}_+\times A} \min\{s,1\}\nu(\mathrm{d}s,\mathrm{d}w) < \infty$$

for any bounded $A \in \mathcal{W}$. The representation (S2) is of paramount importance to prove all our posterior results, and it clarifies the pivotal role of ν in the determination of the distributional properties of μ_c. Kallenberg [2010] provides a very general decomposition for such a measure ν as follows: $\nu(\mathrm{d}s,\mathrm{d}w) = \lambda_w(\mathrm{d}s)\Lambda(\mathrm{d}w)$, where Λ is a σ-finite measure on $(\mathbb{W},\mathcal{W})$ and λ_w is a transition kernel, i.e., $w \to \lambda_w(A)$ is $\mathcal{W}$-measurable for all Borel sets $A \in \mathcal{B}_{\mathbb{R}_+}$ and $A \to \lambda_w(A)$ is a measure on $(\mathbb{R}_+,\mathcal{B}_{\mathbb{R}_+})$. When $\lambda_w(\mathrm{d}s) \equiv \lambda(\mathrm{d}s)$ does not depend on $w \in \mathbb{W}$, we say that the CRM is homogeneous, which is tantamount to saying that the atoms W_i's and the jumps τ_i's are independent random variables. In BNP problems, it is common to suppose that $\Lambda(\mathrm{d}w) = \alpha P(\mathrm{d}w)$, where P is a probability measure on $(\mathbb{W},\mathcal{W})$ and $\alpha > 0$. Two remarkable examples of CRMs are the σ-stable process, which can be recovered by choosing $\lambda(\mathrm{d}s) = \sigma s^{-1-\sigma}\mathrm{d}s$, and the gamma process, which corresponds to the choice $\lambda(\mathrm{d}s) = e^{-s}/s\,\mathrm{d}s$. See also [Lijoi and Prünster, 2010] for additional details and connections with the BNP literature.

In Section S5, we will make use of multivariate CRMs to define a multivariate extension of the Bernoulli process model, called the Bernoulli process model with a condiment. For this reason we now specify what we mean for a multivariate CRM. A vector $\boldsymbol{\mu} = (\mu_1,\ldots,\mu_q)$

of completely random measures is said to be a multivariate CRM if the random variables

$$(\mu_1(A_1), \dots, \mu_q(A_1)), \dots, (\mu_1(A_n), \dots, \mu_q(A_n))$$

are independent for any choice of bounded and disjoint Borel sets $A_1, \dots, A_n \in \mathcal{W}$ and for any $n \geq 1$. A decomposition similar to the one stated in Equation (S1) holds true for multivariate CRMs as well [Kallenberg, 2010]. In the present paper we focus on multivariate CRMs which are functionals of marked Poisson point processes on $\mathbb{R}_+^q \times \mathbb{W}$, i.e.,

$$\boldsymbol{\mu} = \sum_{i \geq 1} \boldsymbol{\tau}_i \delta_{W_i},$$

where $(\boldsymbol{\tau}_i)_{i \geq 1}$ are random jumps in $\mathbb{R}_+^q$ and $(W_i)_{i \geq 1}$ is a sequence of random atoms in $\mathbb{W}$. Such a multivariate CRM has the following Lévy-Khintchine representation which generalizes Equation (S2):

$$\begin{aligned} &\mathbb{E}[e^{-\int_{\mathbb{W}} f_1(w)\mu_1(\mathrm{d}w) - \dots - \int_{\mathbb{W}} f_q(w)\mu_q(\mathrm{d}w)}] \\ &= \exp\left\{ -\int_{\mathbb{W}} \int_{\mathbb{R}_+^q} (1 - e^{-s_1 f_1(w) - \dots - s_q f_q(w)}) \nu_{(q)}(\mathrm{d}s_1, \dots, \mathrm{d}s_q, \mathrm{d}w) \right\} \end{aligned} \tag{S3}$$

for arbitrary measurable functions $f_1, \dots, f_d : \mathbb{W} \to \mathbb{R}_+$. The intensity measure $\nu_{(q)}$ in (S3) is required to simultaneously satisfy

$$\nu_{(q)}(\mathbb{R}_+^q \times \{w\}) = 0 \quad \forall w \in \mathbb{W}$$

and

$$\int_{\mathbb{R}_+ \times A} \min\{||\boldsymbol{s}||, 1\} \nu_{(q)}(\mathrm{d}s_1, \dots, \mathrm{d}s_q, \mathrm{d}w) < \infty,$$

for any bounded $A \in \mathcal{W}$, and having denoted by $||\boldsymbol{s}||$ the Euclidean norm of the vector $\boldsymbol{s} := (s_1, \dots, s_q)$. In the present paper, we will work with a *homogeneous* Lévy intensity measure of the following form $\nu_{(q)}(\mathrm{d}s_1, \dots, \mathrm{d}s_q, \mathrm{d}w) = \lambda_{(q)}(s_1, \dots, s_q)\mathrm{d}s_1 \cdots \mathrm{d}s_q P(\mathrm{d}w)$, where P is a diffuse probability measure on $(\mathbb{W}, \mathcal{W})$ and $\lambda_{(q)} : \mathbb{R}_+^q \to \mathbb{R}_+$ is measurable. See, e.g., [Kallenberg, 2017] for further details.

S2 Posterior analysis for SP priors: proofs and details

In the present section we derive the marginal, posterior and predictive distributions for the Bernoulli process model under a scaled process prior. Specifically we focus on the following statistical model throughout the section:

$$\begin{aligned} Z_n \mid \mu &\stackrel{\text{iid}}{\sim} \text{BeP}(\mu_{\Delta_{1,h}}), \quad \text{for} \quad n = 1, \dots, N \\ \mu_{\Delta_{1,h}} &\sim \text{SP}(\nu, h), \end{aligned} \tag{S4}$$

where $\mu_{\Delta_{1,h}}$ has been defined at the beginning of Section 2.2. In Subsection S2.1 we provide some lemmas regarding SP priors, then Subsection S2.2 is concerned with the Bayesian posterior analysis of the model in (S4).

S2.1 Preparatory lemmas

Some preparatory lemmas are required before the posterior analysis. The first lemma provides the reader with the conditional distribution of $\mu_{\Delta_{1,h}}$ given $\Delta_{1,h}$.

Lemma S1. *Let $\mu_{\Delta_{1,h}} \sim \text{SP}(\nu, h)$, governed by the Lévy intensity measure $\nu(\mathrm{d}s, \mathrm{d}w) = \lambda(s)\mathrm{d}sP(\mathrm{d}w)$ on $\mathbb{R}_+ \times \mathbb{W}$. The conditional distribution of $\mu_{\Delta_{1,h}}$, given $\Delta_{1,h}$, equals the one of a CRM on $(\mathbb{W}, \mathcal{W})$ with Lévy intensity*

$$\Delta_{1,h}\lambda(\Delta_{1,h}s)\mathbb{1}_{(0,1)}(s)\mathrm{d}sP(\mathrm{d}w).$$

Proof. Recall the construction of a SP prior, as detailed in Section 2.2. It starts from an underlying CRM $\mu = \sum_{i\geq 1} \tau_i \delta_{W_i}$ with intensity ν on $\mathbb{R}_+ \times \mathbb{W}$. Moreover, having denoted by $\Delta_1 > \Delta_2 > \dots$ the decreasingly ordered jumps τ_i's of μ, one considers:

$$\mu_{\Delta_1} = \sum_{i\geq 1} \frac{\Delta_{i+1}}{\Delta_1} \delta_{W_{i+1}},$$

and the SP process is defined by a change of measure of the largest jump Δ_1, replaced with the distribution of $\Delta_{1,h}$. As a consequence it is sufficient to prove that $\mu_{\Delta_1} \mid \Delta_1$ is a CRM with Lévy intensity

$$\Delta_1 \lambda(\Delta_1 s) \mathbb{1}_{(0,1)}(s) \mathrm{d}s P(\mathrm{d}w). \tag{S5}$$

In order to prove this remind that $(\Delta_i)_{i\geq 2}|\Delta_1$ are the points of a Poisson process with Lévy intensity $\lambda(s)\mathbb{1}_{(0,\Delta_1)}(s)\mathrm{d}s$, thanks to the representation by Ferguson and Klass [1972]. Therefore, the conditional distribution of μ_{Δ_1}, given Δ_1, may be found by a simple evaluation of the Laplace functional. To this end, consider a measurable function $f : \mathbb{W} \to \mathbb{R}_+$ and compute

$$\begin{aligned}
\mathbb{E}[e^{-\int_{\mathbb{W}} f(w)\mu_{\Delta_1}(\mathrm{d}w)}|\Delta_1] &= \mathbb{E}\left[e^{-\sum_{i\geq 1} f(W_{i+1})\Delta_{i+1}/\Delta_1}|\Delta_1\right] \\
&= \exp\left\{-\int_{\mathbb{W}}\int_0^{+\infty}(1-e^{-f(w)s/\Delta_1})\mathbb{1}_{(0,\Delta_1)}(s)\lambda(s)\mathrm{d}s\; P(\mathrm{d}w)\right\} \\
&= \exp\left\{-\int_{\mathbb{W}}\int_0^{+\infty}(1-e^{-f(w)s})\mathbb{1}_{(0,1)}(s)\lambda(s\Delta_1)\Delta_1\mathrm{d}s\; P(\mathrm{d}w)\right\}
\end{aligned}$$

which is exactly the Laplace functional of a CRM having Lévy intensity (S5). □

We now provide the reader with a sufficient condition to ensure that each Z_n in (S4) is almost surely finite, for any $n \geq 1$.

Lemma S2. *Consider the model in Equation (S4). If*

$$\mathbb{E}\left[\int_0^1 \Delta_{1,h}\lambda(s\Delta_{1,h})\mathrm{d}s\right] < \infty, \tag{S6}$$

then each Z_n displays almost surely finitely many features — i.e. $\sum_{i\geq 1} A_{n,i} < \infty$, almost surely, for every $n \geq 1$.

Proof. For a fixed $n \geq 1$, it is sufficient to show that condition (S6) entails

$$\mathbb{E}\left[\sum_{i=1}^{\infty} A_{n,i}\right] < \infty.$$

The expected value in the previous formula may be computed as follows

$$\mathbb{E}\left[\sum_{i=1}^{\infty} A_{n,i}\right] = \mathbb{E}\left[\mathbb{E}\left[\sum_{i=1}^{\infty} A_{n,i}\middle|\Delta_{1,h}\right]\right] = \mathbb{E}\left[\mathbb{E}[\mu_{\Delta_{1,h}}(\mathbb{W})|\Delta_{1,h}]\right]$$
$$= \mathbb{E}\left[\int_{\mathbb{W}}\int_0^1 s\Delta_{1,h}\lambda(\Delta_{1,h}s)\mathrm{d}sP(\mathrm{d}w)\right] = \mathbb{E}\left[\int_0^1 s\Delta_{1,h}\lambda(\Delta_{1,h}s)\mathrm{d}s\right]$$

where we have applied the Campbell theorem [Kingman, 1992] and Lemma S1 to evaluate the total mass $\mu_{\Delta_{1,h}}(\mathbb{W})$ of $\mu_{\Delta_{1,h}}$. As a consequence, condition (S6) is sufficient for the finiteness of the Bernoulli process Z_n. □

S2.2 Posterior analysis

We start with the marginal distribution of the observations $Z_{1:N}$ induced by the model. Our derivation closely follows the proof in James [2017]. The marginal distribution is the counterpart of the "exchangeable feature probability function" (EFPF) for the Indian Buffet Process (IBP; see, e.g., Broderick et al. [2013]).

Proposition S1 (Joint marginal distribution)**.** *For any $N \geq 1$, let $Z_{1:N}$ be a random sample modeled as the BNP-Bernoulli model (S4), where $\mu_{\Delta_{1,h}} \sim \mathrm{SP}(\nu, h)$. The probability that the observations $Z_{1:N}$ display $K_N = k$ distinct features, labelled by $\{W_1^*, \dots, W_{K_N}^*\}$, with corresponding frequencies $(M_{N,1}, \dots, M_{N,K_N}) = (m_1, \dots, m_k)$, equals*

$$p_k^{(N)}(m_1, \dots, m_k) = \int_0^{+\infty} e^{-\sum_{n=1}^N \phi_n(a)} \prod_{i=1}^k \int_0^1 s^{m_i}(1-s)^{N-m_i} a\lambda(as)\mathrm{d}s\, f_{\Delta_{1,h}}(a)\mathrm{d}a,$$

where $\phi_n(a) = \int_0^1 s(1-s)^{n-1}a\lambda(as)\mathrm{d}s$.

Proof. From the result showed in Lemma S1, we know that conditionally on a known value of $\Delta_{1,h} = a$, the random measure $\mu_{\Delta_{1,h}}$ is completely random. Therefore, we can exploit the result in James [2017, Proposition 3.1] to characterize the marginal distribution of the

feature counts $m_{N,1}, \ldots, m_{N,K_N}$. This is given by

$$
\begin{aligned}
&p_k^{(N)}(m_1, \ldots, m_k \mid \Delta_{1,h} = a) \\
&\qquad = \exp\left\{-\sum_{n=1}^{N} \phi_n(a)\right\} \prod_{i=1}^{k} \left\{\int_0^1 s^{m_i}(1-s)^{N-m_i} a\lambda(as)\mathrm{d}s\right\},
\end{aligned}
\tag{S7}
$$

with $\phi_n(a) = \int_0^1 s(1-s)^{n-1} a\lambda(as)\mathrm{d}s$. Integrating with respect to $f_{\Delta_{1,h}}$ — the mixing distribution of $\Delta_{1,h}$ — yields the desired result. □

Next, we characterize the posterior distribution of the random measure $\mu_{\Delta_{1,h}} \sim \mathrm{SP}(\nu, h)$. The posterior distribution of the law of $\Delta_{1,h}$ is an important ingredient in the study of the predictive properties of the model. We mention that the posterior characterization of Proposition S2 is a consequence of [James et al., 2015, Propositions 2.2] and the results developed by James [2017].

Proposition S2 (Posterior distribution)**.** *For any $N \geq 1$, let $Z_{1:N}$ be a random sample modeled as the BNP-Bernoulli model (S4), where $\mu_{\Delta_{1,h}} \sim \mathrm{SP}(\nu, h)$. Suppose that the observations $Z_{1:N}$ display $K_N = k$ distinct features, labelled by $W_1^*, \ldots, W_{K_N}^*$, with corresponding frequencies $(M_{N,1}, \ldots, M_{N,K_N}) = (m_1, \ldots, m_k)$, then the conditional distribution of $\Delta_{1,h}$, given $Z_{1:N}$, has density function*

$$
g_{\Delta_{1,h}|Z_{1:N}}(a) \propto \exp\left\{-\sum_{n=1}^{N} \phi_n(a)\right\} \prod_{i=1}^{k} \left\{\int_0^1 s^{m_i}(1-s)^{N-m_i} a\lambda(as)\mathrm{d}s\right\} f_{\Delta_{1,h}}(a), \tag{S8}
$$

with $\phi_n(a) = \int_0^1 s(1-s)^{n-1} a\lambda(as)\mathrm{d}s$. Moreover, the posterior distribution of the random measure $\mu_{\Delta_{1,h}}$, conditionally given $Z_{1:N}$ and $\Delta_{1,h}$, equals

$$
\mu_{\Delta_{1,h}} \mid (\Delta_{1,h}, Z_{1:N}) \stackrel{d}{=} \mu'_{\Delta_{1,h}} + \sum_{i=1}^{K_N} J_i \delta_{W_i^*}, \tag{S9}
$$

where

i. $\mu'_{\Delta_{1,h}} | \Delta_{1,h} \sim \mathrm{CRM}(\nu'_{\Delta_{1,h}})$ *with*

$$
\nu'_{\Delta_{1,h}}(\mathrm{d}s, \mathrm{d}w) = (1-s)^N \Delta_{1,h} \lambda(s\Delta_{1,h}) \mathbb{1}_{(0,1)}(s)\mathrm{d}s\, P(\mathrm{d}w); \tag{S10}
$$

ii. $J_{1:K_N}$ *are* K_N *independent random jumps and independent of* $\mu'_{\Delta_{1,h}}$*, with density on* $[0,1]$ *proportional to*

$$f_{J_i|\Delta_{1,h}}(s) \propto (1-s)^{N-m_i} s^{m_i} \Delta_{1,h} \lambda(\Delta_{1,h} s). \tag{S11}$$

Proof. Again, leveraging the result showed in Lemma S1, we know that conditionally on a known value of $\Delta_{1,h}$, the measure $\mu_{\Delta_{1,h}}$ is completely random. Therefore, we can simply apply James [2017, Theorem 3.1] to obtain the posterior distribution of $\mu_{\Delta_{1,h}}|(\Delta_{1,h}, Z_{1:N})$ as described in Equation (S9). Finally, the posterior distribution of the largest jump $\Delta_{1,h}$ conditionally on the observations $Z_{1:N}$ derived in Equation (S8) follows by direct application of Bayes' theorem, recognizing that $f_{\Delta_{1,h}}$ is the prior distribution for $\Delta_{1,h}$, and the distribution in (S7) as the likelihood of the observations $Z_{1:N}|\Delta_{1,h}$. □

Last, we prove the predictive characterization provided in Proposition 2, which has a pivotal role in our analysis, as it is the conceptual starting point in order to study the predictive behavior of the model, and it again follows form [James, 2017].

Proof of Proposition 2. We consider $\zeta \overset{d}{=} \mu_{\Delta_{1,h}}$, thus we are dealing with the model (S4). The posterior distribution of $\Delta_{1,h}$ in (8) follows from (S8), by the argument used in Proposition S2. In order to prove the characterization in Equation (9), we use once again the fact that conditionally on a known value of $\Delta_{1,h}$, $\mu_{\Delta_{1,h}}$ is a completely random measure (see Lemma S1). Thus, we can exploit the results in [James, 2017] to characterize the predictive distribution of Z_{N+1} given the sample $Z_{1:N}$ and the jump $\Delta_{1,h}$. More specifically the form of the predictive distribution in (9) follows by a plain application of James [2017, Proposition 3.2]. □

S3 Posterior analysis for SB-SP priors: proofs and details

Here we provide details and proofs of the results in Section 3.1, i.e. a full Bayesian analysis for the SB-SP prior. More specifically we prove Theorem 1, then we move to characterize the posterior distribution of $\Delta_{1,h_{c,\beta}}$, marginal, predictive and posterior distributions of the SB-SP model.

S3.1 Proof of Theorem 1

The posterior density of $\Delta_{1,h}$, given $Z_{1:N}$, has density proportional to

$$\prod_{n=1}^{N} e^{-\phi_n(a)} \prod_{i=1}^{K_N} \int_0^1 s^{m_{N,i}}(1-s)^{N-m_{N,i}} a\lambda(as)\mathrm{d}s\, f_{\Delta_{1,h}}(a),$$

where we used the notation $\phi_n(a) = \int_0^1 s(1-s)^{n-1} a\lambda(as)\mathrm{d}s$. Hence, there exists a normalizing factor $c(m_{N,1}, \dots, m_{N,k}, N, K_N)$, depending on the sample size N, the distinct number of features K_N and the frequency counts, such that

$$g_{\Delta_{1,h}|Z_{1:N}}(a) = \frac{\prod_{n=1}^{N} e^{-\phi_n(a)} \prod_{i=1}^{K_N} \int_0^1 s^{m_{N,i}}(1-s)^{N-m_{N,i}} a\lambda(as)\mathrm{d}s\, f_{\Delta_{1,h}}(a)}{c(m_{N,1}, \dots, m_{N,K_N}, N, K_N)},$$

or equivalently we can write

$$g^{-1}_{\Delta_{1,h}|Z_{1:N}}(a) \prod_{n=1}^{N} e^{-\phi_n(a)} \prod_{i=1}^{K_N} \int_0^1 s^{m_{N,i}}(1-s)^{N-m_{N,i}} a\lambda(as)\mathrm{d}s\, f_{\Delta_{1,h}}(a) \\ = c(m_{N,1}, \dots, m_{N,K_N}, N, K_N). \tag{S12}$$

If the posterior density $g_{\Delta_{1,h}|Z_{1:N}}(a)$ does not depend on $m_{N,1}, \dots, m_{N,K_N}$, then the function

$$g^{-1}_{\Delta_{1,h}|Z_{1:N}}(a) \prod_{n=1}^{N} e^{-\phi_n(a)}\, g(a) = f_1(a, K_N, N)$$

depends only on K_N, N and a, but not on the frequency counts. Therefore, (S12) boils down to

$$f_1(a, K_N, N) \cdot \prod_{i=1}^{K_N} \int_0^1 s^{m_{N,i}}(1-s)^{N-m_{N,i}} a\lambda(as)\mathrm{d}s = c(m_{N,1}, \ldots, m_{N,K_N}, N, K_N). \quad \text{(S13)}$$

As a consequence, the function on the right hand side of (S13) is independent of a, for any choice of the vector $(m_{N,1}, \ldots, m_{N,K_N}, N, K_N)$. Now we consider $m_{N,1} = \cdots = m_{N,K_N} = m > 0$, and we can say that the function

$$\left[w(a, K_N, N) \int_0^1 s^m (1-s)^{N-m} a\lambda(as)\mathrm{d}s \right]^{K_N} \quad \text{(S14)}$$

does not depend on $a \in \mathbb{R}_+$, where $w(a, K_N, N) = \sqrt[K_N]{f_1(a, K_N, N)}$. We now select $m = N$, thus the function

$$w(a, K_N, N) \int_0^1 s^N a\lambda(as)\mathrm{d}s \quad \text{(S15)}$$

does not depend on $a \in \mathbb{R}_+$. Note that, since $f_{\Delta_{1,h}}$ and λ are functions of class $C^1(\mathbb{R}_+)$, i.e., derivable with continuous derivative, also w is in class $C^1(\mathbb{R}_+)$ with respect to the variable a. Thus, we can take the derivative of (S15), and this is equal to 0:

$$\frac{\mathrm{d}}{\mathrm{d}a} w(a, K_N, N) \int_0^a s^N \lambda(s)\mathrm{d}s a^{-N} - N a^{-N-1} w(a, K_N, N) \int_0^a s^N \lambda(s)\mathrm{d}s + w(a, K_N, N)\lambda(a) = 0$$

which is an ordinary differential equation in w, and it can be easily solved by separation of variables, thus obtaining

$$w(a, K_N, N) = a^N \cdot \frac{R}{\int_0^a s^N \lambda(s)\mathrm{d}s}$$

where $R > 0$ is a suitable constant independent of a. As a consequence, the function in (S14) equals

$$\left[\frac{R}{\int_0^1 s^N \lambda(as)\mathrm{d}s} \cdot \int_0^1 s^m (1-s)^{N-m} \lambda(as)\mathrm{d}s \right]^{K_N}$$

and this is independent of $a \in \mathbb{R}_+$. It is possible to choose $m = N - 1$ in the previous function, and we can state that

$$\int_0^1 s^{N-1}\lambda(as)\mathrm{d}s - \int_0^1 s^N\lambda(as)\mathrm{d}s = C\int_0^1 s^N\lambda(as)\mathrm{d}s$$

where C is constant with respect to a. If one takes the derivative of the previous equation two times with respect to a, then she obtains

$$\lambda(a)(1 - NC) = a\lambda'(a)C,$$

which is an ordinary differential equation in λ that can be solved by separation of variables. In particular we get the following result

$$\lambda(a) = \alpha a^{(1-NC)/C}, \quad \text{for } \alpha > 0. \tag{S16}$$

The exponent of a in (S16) should satisfy

$$\int_0^{+\infty} \min\{1, a\}\lambda(a)\mathrm{d}a < +\infty,$$

from which it is easy to realize that $-2 < (1 - NC)/C < -1$, hence

$$\lambda(a) = \alpha\frac{1}{a^{1+\sigma}}$$

where $\alpha > 0$ and $\sigma \in (0, 1)$. The reverse implication of the theorem is trivially true, hence the proof is completed.

□

S3.2 Detailed derivation of the distribution of $\Delta_{1,h_{c,\beta}}$

We first derive explicitly the distribution of the largest jump given in Equation (6). This follows from direct application of the law of the largest jump,

$$F_{\Delta_1}(\mathrm{d}a) = \exp\left\{-\int_a^\infty \lambda_\sigma(s)\mathrm{d}s\right\}\lambda_\sigma(a)\mathrm{d}a$$

when the Lévy measure is

$$\lambda_\sigma(s)\mathrm{d}s = \sigma s^{-\sigma-1}\mathbb{1}_{\mathbb{R}_+}(s)\mathrm{d}s.$$

Having denoted by f_{Δ_1} the density function of F_{Δ_1}, we get

$$\begin{aligned} f_{\Delta_1}(a) &= \lambda_\sigma(a)e^{-\Lambda(a)}\mathbb{1}_{\mathbb{R}_+}(a) = \sigma a^{-\sigma-1}\exp\left\{-\int_a^\infty \sigma u^{-1-\sigma}\mathrm{d}u\right\}\mathbb{1}_{\mathbb{R}_+}(a) \\ &= \sigma a^{-\sigma-1}e^{-a^{-\sigma}}\mathbb{1}_{\mathbb{R}_+}(a). \end{aligned}$$

From direct inspection, we recognize that this is the density function of $\Delta_1 = T^{-1/\sigma}$, where T is a Gamma with parameters $(1,1)$. The mixing measure is then obtained by tilting the density f_{Δ_1} as follows:

$$f_{\Delta_{1,h_{c,\beta}}}(a) \propto f_{\Delta_1}(a)h_{c,\beta}(a) = \sigma a^{-\sigma(c+1)-1}\exp\left\{-\beta a^{-\sigma}\right\}\mathbb{1}_{\mathbb{R}_+}(a),$$

i.e. letting

$$h_{c,\beta}(a) \propto a^{-\sigma c}\exp\left\{-(\beta-1)a^{-\sigma}\right\}.$$

By integration, we get the normalizing constant:

$$\int_0^\infty a^{-\sigma(c+1)-1}\exp\left\{-\beta a^{-\sigma}\right\}\mathrm{d}a = \frac{\Gamma(c+1)}{\sigma\beta^{c+1}}.$$

from which

$$f_{\Delta_{1,h_{c,\beta}}}(a) = \frac{\sigma\beta^{c+1}}{\Gamma(c+1)}a^{-\sigma(c+1)-1}\exp\left\{-\beta a^{-\sigma}\right\}\mathbb{1}_{\mathbb{R}_+}(a). \tag{S17}$$

S3.3 Posterior distribution of SB-SP priors

Here we characterize the posterior distribution of SB-SP priors: the result is not included in the paper, but we think it is useful to have a full picture on SB-SP priors from a Bayesian viewpoint.

Proposition S3. *For $N \geq 1$ let $Z_{1:N}$ be a random sample modeled as the BNP-Bernoulli model (1), with $\zeta \sim \text{SB-SP}(\sigma, c, \beta)$. If $Z_{1:N}$ displays $K_N = k$ distinct features $\{W_1^*, \ldots, W_{K_N}^*\}$, each feature W_i^* appearing exactly $M_{N,i} = m_i$ times in the samples, then the conditional distribution of $\Delta_{1,h_{c,\beta}}$, given $Z_{1:N}$, has a density function of the form*

$$g_{\Delta_{1,h_{c,\beta}}\,|\,Z_{1:N}}(a) = \sigma \frac{(\beta + \gamma_0^{(N)})^{k+c+1}}{\Gamma(k+c+1)} a^{-k\sigma-(c+1)\sigma-1} \exp\{-a^{-\sigma}(\beta + \gamma_0^{(N)})\}, \tag{S18}$$

where $\gamma_0^{(n)} = \sigma \sum_{1 \leq i \leq n} B(1-\sigma, i)$, with $B(\cdot,\cdot)$ denoting the (standard) Beta function. Moreover, the conditional distribution of ζ, given $(\Delta_{1,h_{c,\beta}}, Z_{1:N})$, coincides with the distribution of

$$\zeta \,|\, (\Delta_{1,h_{c,\beta}}, Z_{1:N}) \overset{d}{=} \mu'_{\Delta_{1,h_{c,\beta}}} + \sum_{i=1}^{K_N} J_i \delta_{W_i^*}, \tag{S19}$$

where:

i) $\mu'_{\Delta_{1,h_{c,\beta}}}$ *is a discrete random measure such that* $\mu'_{\Delta_{1,h_{c,\beta}}} \,|\, \Delta_{1,h_{c,\beta}} \sim \text{CRM}(\nu'_{\Delta_{1,h_{c,\beta}}})$, *with* $\nu'_{\Delta_{1,h_{c,\beta}}}$ *being*

$$\nu'_{\Delta_{1,h_{c,\beta}}}(\mathrm{d}s,\, \mathrm{d}w) = \Delta^{-\sigma}_{1,\Delta_{1,h_{c,\beta}}}(1-s)^N \sigma s^{-1-\sigma} \mathbb{1}_{(0,1)}(s) \mathrm{d}s P(\mathrm{d}w); \tag{S20}$$

ii)

$$J_i | \Delta_{1,h_{c,\beta}} \sim \text{Beta}(m_i - \sigma, N - m_i + 1), \tag{S21}$$

where Beta *denotes the beta distribution.*

Proof. We apply Proposition S2, which describes the general posterior distribution of a SP process. We first compute the posterior distribution (8) of the largest jump conditionally on observations $Z_{1:N}$. To do so we specify (S8) in our case, and we first compute the exponent $\phi_n(a)$. In our case the Lévy density equals $\lambda_\sigma(s) = \sigma s^{-\sigma-1}$ and the mixing density of $\Delta_{1,h_{c,\beta}}$ is provided in Equation (S17), thus the exponent ϕ_n takes the form

$$\phi_n(a) = \sigma \int_0^1 s(1-s)^{n-1} a^{-\sigma} s^{-\sigma-1} \mathrm{d}s = \sigma a^{-\sigma} B(1-\sigma, n). \tag{S22}$$

Recalling the shorthand notation $\gamma_0^{(N)} = \sigma \sum_{1 \le n \le N} B(1-\sigma, n)$, the posterior distribution of $\Delta_{1,h_{c,\beta}}$ is then proportional to

$$a^k \exp\left\{-a^{-\sigma}\gamma_0^{(N)}\right\} \prod_{i=1}^{k} \int_0^1 t^{m_i}(1-t)^{N-m_i}\lambda_\sigma(at)\mathrm{d}t\, f_{\Delta_{1,h_{c,\beta}}}(a)$$
$$\propto a^{-\sigma(k+c+1)-1} \exp\left\{-a^{-\sigma}\left[\beta + \gamma_0^{(N)}\right]\right\},$$

where $f_{\Delta_{1,h_{c,\beta}}}$ has been specified in (S17). As a consequence we get

$$\Delta_{1,h_{c,\beta}}^{-\sigma} \mid Z_{1:N} \sim \mathrm{Gamma}\left(k+c+1, \beta + \gamma_0^{(N)}\right),$$

which corresponds to the posterior density in (S18). The characterization of the posterior distribution in (S19) is an easy consequence of Proposition S2, by a specialization of this result with the choice $\lambda(s) = \lambda_\sigma(s) = \sigma s^{-\sigma-1}$ for the underlying Lévy intensity. □

S3.4 Proof of Proposition 3

The predictive characterization is a simple consequence of the general characterization in Proposition 2 with the SB-SP specifications $\lambda(s) = \lambda_\sigma(s) = \sigma s^{-\sigma-1}$. □

S3.5 Proof of Proposition 4

We apply Proposition S1 to obtain the marginal distribution for the SB-SP prior. Conditionally on $\Delta_{1,h_{c,\beta}} = a$, using the form $\phi_n(a)$ derived in (S22), the marginal distribution is given by

$$p_k^{(N)}(m_1, \ldots, m_k \mid \Delta_{1,h_{c,\beta}} = a) = (\sigma a^{-\sigma})^k \exp\left\{-a^{-\sigma}\gamma_0^{(N)}\right\} \prod_{i=1}^{k} \int_0^1 s^{m_i-\sigma-1}(1-s)^{N-m_i}\mathrm{d}s,$$

that may be written in terms of the Beta function as follows

$$p_k^{(N)}(m_1, \ldots, m_k \mid \Delta_{1,h_{c,\beta}} = a) = (\sigma a^{-\sigma})^k \exp\left\{-a^{-\sigma}\gamma_0^{(N)}\right\} \prod_{i=1}^{k} B(m_i - \sigma, N - m_i + 1).$$

Last, we obtain the marginal distribution in Equation (10) by randomizing with respect to the mixing distribution of the largest jump given in Equation (S17). We need to compute

$$\begin{aligned}
p_k^{(N)}(m_1, \dots, m_k) &= \int_0^\infty p_k^{(N)}(m_1, \dots, m_k \mid \Delta_{1,h_{c,\beta}} = a) f_{\Delta_{1,h_{c,\beta}}}(a)\mathrm{d}a \\
&= \frac{\sigma^{k+1}\beta^{c+1}}{\Gamma(c+1)} \prod_{i=1}^k B(m_i - \sigma, N - m_i + 1) \\
&\times \int_0^\infty a^{-\sigma(k+c+1)-1} \exp\left\{-a^{-\sigma}\left[\beta + \gamma_0^{(N)}\right]\right\} \mathrm{d}a \\
&= \frac{\sigma^k \beta^{c+1}}{(\beta + \gamma_0^{(N)})^{k+c+1}} \frac{\Gamma(k+c+1)}{\Gamma(c+1)} \prod_{i=1}^k B(m_i - \sigma, N - m_i + 1),
\end{aligned}$$

and the thesis now follows. □

S4 Estimation of the unseen features via SB-SP priors: proofs

Here we detail the proofs of Section 3.2, which is devoted to the unseen-features problem under the SB-SP prior.

S4.1 Proof of Theorem 2

We first focus on the proof of (13), i.e. the posterior distribution of $U_N^{(M)}$. In order to do this we exploit the predictive characterization provided in Proposition 3 to evaluate the probability generating function (PGF) of the random variable $U_N^{(M)}$ *a posteriori*, conditionally on the sample $Z_{1:N}$. We denote the PGF as $\mathcal{G}_{U_N^{(M)}}(\,\cdot\,)$. If t belongs to a neighborhood of the origin, then one has

$$\mathcal{G}_{U_N^{(M)}}(t) = \mathbb{E}\left[t^{U_N^{(M)}} \mid Z_{1:N}\right] = \mathbb{E}\left[\mathbb{E}\left[t^{U_N^{(M)}} \mid Z_{1:N}, \Delta_{1,h_{c,\beta}}\right] \mid Z_{1:N}\right] \tag{S23}$$

where we have applied the tower property of the conditional expectation. We now observe that, conditionally on $Z_{1:N}$ and $\Delta_{1,h_{c,\beta}}$, the random variable $U_N^{(M)}$ may be represented as

$$U_N^{(M)} | (Z_{1:N}, \Delta_{1,h_{c,\beta}}) \stackrel{\mathrm{d}}{=} \sum_{i\geq 1} \mathbb{1}\left(\sum_{m=1}^{M} A'_{N+m,i} > 0\right),$$

where we used the representation given in Proposition 3. Here, independently across i, $A'_{N+m,i}$ is a Bernoulli random variable with parameter ρ'_i, conditionally on the random measure $\mu'_{\Delta_{1,h_{c,\beta}}} = \sum_{i\geq 1} \rho'_i \delta_{W'_i}$ with Lévy intensity $\sigma \Delta_{1,h_{c,\beta}}^{-\sigma}(1-s)^N s^{-1-\sigma} \mathbb{1}_{(0,1)}(s) \mathrm{d}s P(\mathrm{d}w)$. We now focus on the evaluation of the expected value in Equation (S23):

$$\begin{aligned}
&\mathbb{E}\left[t^{U_N^{(M)}} \mid Z_1, \ldots, Z_N, \Delta_{1,h_{c,\beta}}\right] \\
&\quad = \mathbb{E}\left[\mathbb{E}\left[\prod_{i\geq 1}\left((t-1)\mathbb{1}\left\{\sum_{m=1}^{M} A'_{N+m,i} > 0\right\} + 1\right) \Big| \mu'_{\Delta_{1,h_{c,\beta}}}\right]\right] \\
&\quad = \mathbb{E}\left[\prod_{i\geq 1}\left[(t-1)\mathbb{P}\left(\sum_{m=1}^{M} A'_{N+m,i} > 0 \mid \mu'_{\Delta_{1,h_{c,\beta}}}\right) + 1\right]\right] \\
&\quad = \mathbb{E}\left[\prod_{i\geq 1}\left[(t-1)\left\{1 - \prod_{m=1}^{M} \mathbb{P}(A'_{N+m,i} = 0 \mid \mu'_{\Delta_{1,h_{c,\beta}}})\right\} + 1\right]\right],
\end{aligned}$$

where we applied the independence of the Bernoulli random variables $A'_{N+m,i}$s, conditionally on $\mu'_{\Delta_{1,h_{c,\beta}}}$. We now recall that $\mu'_{\Delta_{1,h_{c,\beta}}}$ is a CRM with a known Lévy measure and that the $A'_{N+m,i}$s are Bernoulli with parameter ρ'_i to obtain

$$\begin{aligned}
&\mathbb{E}\left[t^{U_N^{(M)}} \mid Z_1, \ldots, Z_N, \Delta_{1,h_{c,\beta}}\right] \\
&\quad = \mathbb{E}\left[\prod_{i\geq 1}((t-1)(1-(1-\rho'_i)^M) + 1)\right] \\
&\quad = \mathbb{E}\left[\exp\left\{\sum_{i\geq 1} \log\left[(t-1)(1-(1-\rho'_i)^M) + 1\right]\right\}\right] \\
&\quad = \exp\left\{-(1-t)\int_0^1 (1-(1-s)^M)(1-s)^N \Delta_{1,h_{c,\beta}}^{-\sigma} \sigma s^{-1-\sigma} \mathrm{d}s\right\}. \\
&\quad = \exp\left\{-(1-t)\Delta_{1,h_{c,\beta}}^{-\sigma} \gamma_N^{(M)}\right\},
\end{aligned}$$

where we used the identity

$$\int_0^1 \left[1-(1-s)^M\right](1-s)^N s^{-1-\sigma}\mathrm{d}s = \sum_{m=1}^{M} B(1-\sigma, N+m).$$

We replace this expression in Equation (S23) to obtain

$$\mathcal{G}_{U_N^{(M)}}(t) = \mathbb{E}[\exp\{-(1-t)\Delta_{1,h_{c,\beta}}^{-\sigma}\gamma_N^{(M)}\} \mid Z_{1:N}]. \tag{S24}$$

The results now follows by integrating with respect to the posterior distribution of $\Delta_{1,h_{c,\beta}}^{-\sigma}$, given in Equation (S18):

$$\begin{aligned}
\mathcal{G}_{U_N^{(M)}}(t) &= \frac{(\beta+\gamma_0^{(N)})^{K_N+c+1}}{\Gamma(K_N+c+1)}\int_0^\infty \exp\left\{-(1-t)\gamma_N^{(M)}x\right\} x^{K_N+c}e^{-(\beta+\gamma_0^{(N)})x}\mathrm{d}x \\
&= \frac{(\beta+\gamma_0^{(N)})^{K_N+c+1}}{\Gamma(K_N+c+1)}\frac{\Gamma(K_N+c+1)}{(\beta+\gamma_0^{(N)}+(1-t)\gamma_N^{(M)})^{K_N+c+1}} \\
&= \left(\frac{\beta+\gamma_0^{(N)}}{\beta+\gamma_0^{(N+M)}-t\gamma_N^{(M)}}\right)^{K_N+c+1} = \left(\frac{1-p_N^{(M)}}{1-tp_N^{(M)}}\right)^{K_N+c+1},
\end{aligned}$$

for any $|t| < 1/p_N^{(M)}$, where $p_N^{(M)} := \gamma_N^{(M)}/(\beta+\gamma_0^{(N+M)}) \leq 1$. This is the probability generating function of a negative binomial distribution where K_N+c+1 is the number of failures, and $p_N^{(M)}$ is the success probability in each experiment.

We now apply similar arguments to derive the posterior distribution of $U_N^{(M,r)}$, provided in (14). Again, we calculate the probability generating function of $U_N^{(M,r)}$ *a posteriori*, denoted here as $\mathcal{G}_{U_N^{(M,r)}}(\cdot)$. If t belongs to a neighborhood of the origin, then one has

$$\mathcal{G}_{U_N^{(M,r)}}(t) = \mathbb{E}\left[t^{U_N^{(M,r)}} \mid Z_{1:N}\right] = \mathbb{E}\left[\mathbb{E}\left[t^{U_N^{(M,r)}} \mid Z_{1:N}, \Delta_{1,h_{c,\beta}}\right] \mid Z_{1:N}\right]. \tag{S25}$$

It is now easy to see that, conditionally on $Z_1,\ldots,Z_N,\Delta_{1,h_{c,\beta}}$, the random variable $U_N^{(M,r)}$ may be written as

$$U_N^{(M,r)}|Z_1,\ldots,Z_N,\Delta_{1,h_{c,\beta}} \overset{\mathrm{d}}{=} \sum_{i\geq 1}\mathbb{1}\left\{\sum_{m=1}^{M} A'_{N+m,i} = r\right\}$$

by applying Proposition 3. With the same notation used in the first part of the proof, we recall that the $A'_{N+m,i}$s are independent Bernoulli variables with parameters ρ'_i, conditionally on the CRM $\mu'_{\Delta_{1,h_{c,\beta}}} = \sum_{i\geq 1} \rho'_i \delta_{W'_i}$ with Lévy intensity $\sigma\Delta_{1,h_{c,\beta}}^{-\sigma}(1-s)^N s^{-1-\sigma}\mathbb{1}_{(0,1)}(s)\mathrm{d}sP(\mathrm{d}w)$. Across i, the random variables

$$S_{M,i} := \sum_{m=1}^{M} A'_{N+m,i}$$

are independent, each one distributed as a binomial with parameters M and success probability ρ'_i. We then evaluate the expected value appearing in (S25) as follows:

$$\begin{aligned}
\mathbb{E}\left[t^{U_N^{(M,r)}} \mid Z_{1:N}, \Delta_{1,h_{c,\beta}}\right] &= \mathbb{E}\left[\mathbb{E}\left[\prod_{i\geq 1}\left((t-1)\mathbb{1}\left\{\sum_{m=1}^{M} A'_{N+m,i} = r\right\} + 1\right) \Big| \mu'_{\Delta_{1,h_{c,\beta}}}\right]\right] \\
&= \mathbb{E}\left[\prod_{i\geq 1}\left[(t-1)\mathbb{P}(S_{M,i} = r \mid \mu'_{\Delta_{1,h_{c,\beta}}}) + 1\right]\right] \\
&= \mathbb{E}\left[\prod_{i\geq 1}\left[(t-1)\binom{M}{r}(\rho'_i)^r(1-\rho'_i)^{M-r} + 1\right]\right].
\end{aligned}$$

Since $\mu'_{\Delta_{1,h_{c,\beta}}}$ is a CRM with a known Lévy measure, we can evaluate the previous expected value:

$$\begin{aligned}
&\mathbb{E}\left[t^{U_N^{(M,r)}} \mid Z_1, \ldots, Z_N, \Delta_{1,h_{c,\beta}}\right] \\
&= \mathbb{E}\left[\exp\left\{\sum_{i\geq 1}\log\left((t-1)\binom{M}{r}(\rho'_i)^r(1-\rho'_i)^{M-r} + 1\right)\right\}\right] \\
&= \exp\left\{-(1-t)\binom{M}{r}\int_0^1 s^{r-\sigma-1}(1-s)^{M+N-r}\mathrm{d}s\sigma\Delta_{1,h_{c,\beta}}^{-\sigma}\right\} \\
&= \exp\left\{-(1-t)\Delta_{1,h_{c,\beta}}^{-\sigma}\sigma\binom{M}{r}B(r-\sigma, M+N-r+1)\right\} \\
&= \exp\left\{-(1-t)\Delta_{1,h_{c,\beta}}^{-\sigma}\rho_N^{(M,r)}\right\},
\end{aligned}$$

where we used the notation introduced in the statement of the theorem, i.e. $\rho_N^{(M,r)} = \sigma\binom{M}{r}B(r-\sigma, M+N-r+1)$. Then the probability generating function in Equation (S25) is obtained by integrating with respect to the posterior distribution of the largest jump

provided in (S18):

$$
\begin{aligned}
\mathcal{G}_{U_N^{(M,r)}}(t) &= \int_0^\infty \exp\left\{-(1-t)x\rho_N^{(M,r)}\right\} \cdot \frac{(\beta+\gamma_0^{(N)})^{K_N+c+1}}{\Gamma(K_N+c+1)} x^{K_N+c} e^{-(\beta+\gamma_0^{(N)})x} \mathrm{d}x \\
&= \frac{\Gamma(K_N+c+1)}{(\beta+\gamma_0^{(N)}+(1-t)\rho_N^{(M,r)})^{K_N+c+1}} \cdot \frac{(\beta+\gamma_0^{(N)})^{K_N+c+1}}{\Gamma(K_N+c+1)} \\
&= \left(\frac{\beta+\gamma_0^{(N)}}{\beta+\gamma_0^{(N)}+\rho_N^{(M,r)}-t\rho_N^{(M,r)}}\right)^{K+c+1} = \left(\frac{1-p_N^{(M,r)}}{1-tp_N^{(M,r)}}\right)^{K_N+c+1}
\end{aligned}
$$

for any $|t| < 1/p_N^{(M,r)}$, where we have set

$$
p_N^{(M,r)} := \frac{\rho_N^{(M,r)}}{\beta+\rho_N^{(M,r)}+\gamma_0^{(N)}}.
$$

Then we conclude that the posterior distribution of $U_N^{(M,r)}$ is a negative binomial distribution where K_N+c+1 is the number of failures, and $p_N^{(M,r)}$ is the success probability in each experiment.

□

S4.2 Proof of Theorem 3

In order to prove this result, we first exploit the Lévy continuity theorem, to obtain a convergence in distribution, and later strengthen this result to show that the convergence holds true also in the almost-sure sense. For the convergence in distribution, thanks to Theorem 2, the characteristic function of $U_N^{(M)}/M^\sigma \mid Z_{1:N}$ is given by

$$
\Phi_{U_N^{(M)}/M^\sigma}(t) = \left(\frac{1-p_N^{(M)}}{1-p_N^{(M)}e^{it/M^\sigma}}\right)^{K_N+c+1}
$$

where $t \in \mathbb{R}$, K_N is the number of distinct features in $Z_{1:N}$ and $p_N^{(M)} = \gamma_N^{(M)}/(\gamma_0^{(N)}+\gamma_N^{(M)}+\beta)$. The quantity above can be rewritten as

$$
\Phi_{U_N^{(M)}/M^\sigma}(t) = \left(\frac{\beta+\gamma_0^{(N)}}{\beta+\gamma_0^{(N)}+\gamma_N^{(M)}-\gamma_N^{(M)}e^{it/M^\sigma}}\right)^{K_N+c+1} .
$$

We can exploit Masoero et al. [2021, Lemma 1] to determine the asymptotic expansion $\gamma_N^{(M)} = M^\sigma \Gamma(1-\sigma)(1+O(M^{-\sigma}))$ as $M \to +\infty$, having used the big-O notation. Thus, using the asymptotic expansion of the exponential function, one has

$$\begin{aligned}\Phi_{U_N^{(M)}/M^\sigma}(t) &= \left(\frac{\beta+\gamma_0^{(N)}}{\beta+\gamma_0^{(N)}+\gamma_N^{(M)}-\gamma_N^{(M)}(1+itM^{-\sigma}+O(M^{-2\sigma}))}\right)^{K_N+c+1} \\ &= \left(\frac{\beta+\gamma_0^{(N)}}{\beta+\gamma_0^{(N)}-\gamma_N^{(M)}itM^{-\sigma}+O(M^{-\sigma})}\right)^{K_N+c+1} \\ &= \left(\frac{\beta+\gamma_0^{(N)}}{\beta+\gamma_0^{(N)}-it\Gamma(1-\sigma)+O(M^{-\sigma})}\right)^{K_N+c+1}\end{aligned}$$

which converges to the characteristic function of a gamma random variable with parameters $(K_N+c+1, (\gamma_0^{(N)}+\beta)/\Gamma(1-\sigma))$ as $M \to +\infty$. This proves that

$$U_N^{(M)}/M^\sigma \mid Z_{1:N} \overset{\mathrm{d}}{\to} W_N, \quad \text{where } W_N \sim \text{Gamma}\left(K_N+c+1, \frac{\beta+\gamma_0^{(N)}}{\Gamma(1-\sigma)}\right).$$

In order to prove convergence in the almost sure sense, we exploit the corresponding results proved for the stable beta-Bernoulli process in Masoero et al. [2021, Theorem 2] for the statistic $U_N^{(M)}$. We first notice that if we condition on the value of the largest jump $\Delta_{1,h_{c,\beta}}$, then the SB-SP-Bernoulli is a completely random measure whose asymptotic behavior is analogous to the stable beta-Bernoulli process. Thus, specializing the almost sure convergence results given in Masoero et al. [2021, Theorem 2], a posteriori, we have

$$\mathbb{P}\left(\lim_{M\to+\infty}\frac{U_N^{(M)}}{M^\sigma} = a^{-\sigma}\Gamma(1-\sigma)\Big|Z_{1:N}, \Delta_{1,h_{c,\beta}} = a\right) = 1. \tag{S26}$$

The probability limit for the model in which the largest jump is random is obtained by observing that

$$\begin{aligned}&\mathbb{P}\left(\lim_{M\to+\infty}\frac{U_N^{(M)}}{M^\sigma} = \Delta_{1,h_{c,\beta}}^{-\sigma}\Gamma(1-\sigma)\Big|Z_{1:N}\right) \\ &\quad = \mathbb{E}\left[\mathbb{P}\left(\lim_{M\to+\infty}\frac{U_N^{(M)}}{M^\sigma} = \Delta_{1,h_{c,\beta}}^{-\sigma}\Gamma(1-\sigma)\Big|Z_{1:N}, \Delta_{1,h_{c,\beta}}\right)\Big|Z_{1:N}\right] \overset{(S26)}{=} 1,\end{aligned}$$

in other words $U_N^{(M)}/M^\sigma$ converges almost surely to the random variable $\Delta_{1,h_{c,\beta}}^{-\sigma}\Gamma(1-\sigma)$, with respect to the conditional probability $\mathbb{P}$ given $Z_{1:N}$. Note also that the posterior distribution of $\Delta_{1,h_{c,\beta}}^{-\sigma}\Gamma(1-\sigma)$ is a Gamma with parameters

$$\left(K_N + c + 1, \frac{\beta + \gamma_0^{(N)}}{\Gamma(1-\sigma)}\right),$$

thus the a.s. convergence in (17) now follows.

We proceed along the same lines as to show the validity of (18). First, we show the convergence in distribution of $U_N^{(M,r)}$ using the characteristic function, and then we show that the result also holds in an almost sure sense. From Theorem 2, the characteristic function of $U_N^{(M,r)}/M^\sigma \mid Z_{1:N}$ is given by

$$\Phi_{U_N^{(M,r)}/M^\sigma}(t) = \left(\frac{1 - p_N^{(M,r)}}{1 - p_N^{(M,r)} e^{it/M^\sigma}}\right)^{K_N+c+1}$$

where $t \in \mathbb{R}$, and $p_N^{(M)} = \rho_N^{(M,r)}/(\gamma_0^{(N)} + \rho_N^{(M,r)} + \beta)$, and $\rho_N^{(M,r)}$ was defined in the statement of Theorem 2. The expression above is equivalent to

$$\Phi_{U_N^{(M,r)}/M^\sigma}(t) = \left(\frac{\beta + \gamma_0^{(N)}}{\beta + \gamma_0^{(N)} + \rho_N^{(M,r)}(1 - e^{it/M^\sigma})}\right)^{K_N+c+1}.$$

Thanks to the well-known asymptotic relation for the ratio of gamma functions, it is easy to see that

$$\rho_N^{(M,r)} = \frac{\sigma}{r!}\Gamma(r-\sigma)(r-\sigma)\frac{\Gamma(M+1)}{\Gamma(M+1-r)}\frac{\Gamma(N+M+2-r)}{\Gamma(N+M+2-\sigma)} = \frac{\sigma}{r!}\Gamma(r-\sigma)M^\sigma(1+O(M^{-1}))$$

as $M \to +\infty$. Hence, the characteristic function under study boils down to

$$\begin{aligned}\Phi_{U_N^{(M,r)}/M^\sigma}(t) &= \left(\frac{\beta + \gamma_0^{(N)}}{\beta + \gamma_0^{(N)} + \sigma\Gamma(r-\sigma)(r!)^{-1}M^{-\sigma}(1+O(M^{-1}))(1 - e^{it/M^\sigma})}\right)^{K_N+c+1} \\ &= \left(\frac{\beta + \gamma_0^{(N)}}{\beta + \gamma_0^{(N)} - \sigma\Gamma(r-\sigma)(r!)^{-1}it + O(M^{-\sigma})}\right)^{K_N+c+1}\end{aligned}$$

which converges, as $M \to +\infty$, to the characteristic function of a gamma random variable with parameters as in the thesis. The almost sure statement of (18) goes along similar lines, indeed one can exploit the convergence theorems proved by Masoero et al. [2021] to state that

$$\mathbb{P}\left(\lim_{M \to +\infty} \frac{U_N^{(M,r)}}{M^\sigma} = \frac{\sigma(1-\sigma)_{(r-1)}}{r!} \Delta_{1,h_{c,\beta}}^{-\sigma} \Gamma(1-\sigma) \Big| Z_{1:N}, \Delta_{1,h_{c,\beta}} \right) = 1.$$

Exactly as before, one can conclude that

$$\mathbb{P}\left(\lim_{M \to +\infty} \frac{U_N^{(M,r)}}{M^\sigma} = \frac{\sigma(1-\sigma)_{(r-1)}}{r!} \Delta_{1,h_{c,\beta}}^{-\sigma} \Gamma(1-\sigma) \Big| Z_{1:N} \right) = 1,$$

where the posterior distribution of the limiting random variable is a gamma with the same parameters as in the statement of the theorem (Equation (18)).

□

S5 Multivariate extension

In the present section we discuss the multivariate version of the Bernoulli process, which we call the Bernoulli process with a condiment or the simple multinomial process, using the terminology of James [2017]. We first revise the model of James [2017] and the associated prior, called stable-Beta-Dirichlet process, then we move to introduce a new scaled prior for the model. In both the cases, we determine closed-form results to face prediction of new features with condiments. These models are extremely important in genomics to account for the presence of variants at certain genomic loci with a specific characteristic (or condiment). See, e.g., Lee et al. [2016].

S5.1 Bernoulli process with a condiment

The IBP process with a condiment has been introduced by James [2017] and we remind the definition here. For $q = 1, 2, \ldots$, we define the vector of probabilities $\boldsymbol{p} = (p_1, \ldots, p_q)$ taking values in the following set

$$S_q = \{\boldsymbol{s} := (s_1, \ldots, s_q) : \; s_j > 0 \text{ as } j = 1, \ldots, q, \; |\boldsymbol{s}| := \sum_{j=1}^{q} s_j < 1\}$$

where for a generic vector $\boldsymbol{s}$, $|\boldsymbol{s}| = \sum_{j=1}^{q} s_j$ denotes the L^1 norm of the vector. For a fixed vector $\boldsymbol{p} \in S_q$, we also define the *simple multinomial* distribution $\mathsf{M}(1, \boldsymbol{p})$. A vector $\boldsymbol{A} = (A_1, \ldots, A_q) \in \{0, 1\}^q$ is said to have the *simple multinomial* distribution with parameter vector $\boldsymbol{p}$ iff it has the following probability mass function

$$\mathbb{P}(\boldsymbol{A} = \boldsymbol{a}) = \mathbb{P}(A_1 = a_1, \ldots, A_q = a_q) = \begin{cases} \prod_{j=1}^{q} p_j^{a_j} \cdot (1 - |\boldsymbol{p}|)^{1-|\boldsymbol{a}|} & \text{if } |\boldsymbol{a}| \leq 1 \\ 0 & \text{if } |\boldsymbol{a}| > 1 \end{cases}$$

and we will write $\boldsymbol{A} \sim \mathsf{M}(1, \boldsymbol{p})$. In other words $\boldsymbol{A}$ concentrates on the vectors of $\{0, 1\}^q$ for which at most one element is equal to 1 and all the other entries are zero.

The Bernoulli process with a condiment assumes that each observation $\boldsymbol{Z}$ is a multivariate $\{0, 1\}^q$-valued stochastic process

$$\boldsymbol{Z}(w) = \sum_{i \geq 1} \boldsymbol{A}_i \delta_{w_i}(w)$$

where $(w_i)_{i \geq 1}$ are features in $\mathbb{W}$ and $(\boldsymbol{A}_i)_{i \geq 1}$ are independent simple multinomial random variables with parameter vector $\boldsymbol{p}_i = (p_{i,1}, \ldots, p_{i,q})$ as $i = 1, 2, \ldots$. Here $|\boldsymbol{p}_i|$ represents the probability that an individual displays feature w_i, while $p_{i,j}$ is the probability that the individual exhibits feature w_i with condiment $j \in \{1, \ldots, q\}$. Thus, $\boldsymbol{Z}$ is termed a *simple mutlinomial process* with parameter $\boldsymbol{\zeta} = \sum_{i \geq 1} \boldsymbol{p}_i \delta_{w_i}$, and it is denoted by $\mathsf{MP}(\boldsymbol{\zeta})$. In order

to carry out BNP inference, we need to specify a distribution for the discrete measure $\boldsymbol{\zeta}$. Thus, we obtain a multivariate version of the model (1):

$$\begin{aligned} \boldsymbol{Z}_n|\boldsymbol{\zeta} &\overset{\text{iid}}{\sim} \mathsf{MP}(\boldsymbol{\zeta}) \quad n=1,\dots,N \\ \boldsymbol{\zeta} &\sim \mathscr{Z} \end{aligned} \tag{S27}$$

where $\mathscr{Z}$ denotes the distribution of the discrete random measure $\boldsymbol{\zeta}$.

S5.2 Priors based on multivariate CRMs

In this section we consider a class of priors $\mathscr{Z}$ in (S27) defined by James [2017] and based on a multivariate extension of CRMs (see Daley and Vere-Jones [2008]). In particular consider a multivariate CRM on $\mathbb{W}$:

$$\boldsymbol{\mu} = \sum_{i\geq 1} \boldsymbol{\rho}_i \delta_{W_i}$$

where $\boldsymbol{\rho}_i = (\rho_{i,1},\dots,\rho_{i,q})$ is a vector of $[0,1]$-valued random jumps with the property $\sum_{i\geq 1} |\boldsymbol{\rho}_i| < +\infty$, the W_i's are i.i.d. $\mathbb{W}$-valued random locations independent of the $\boldsymbol{\rho}_i$'s. Under this nonparametric prior each observation $\boldsymbol{Z}_n$ in (S27) admits the representation $\boldsymbol{Z}_n|\boldsymbol{\mu} = \sum_{i\geq 1} \boldsymbol{A}_{n,i}\delta_{W_i}$, where $\boldsymbol{A}_{n,i} = (A_{n,i,1},\dots,A_{n,i,q})|\boldsymbol{\mu} \overset{\text{ind}}{\sim} \mathsf{M}(1,\boldsymbol{\rho}_i)$. Note that the random measure $\boldsymbol{\mu}$ equals the vector of random measures $(\mu_1,\dots,\mu_q)$, where

$$\mu_j = \sum_{i\geq 1} \rho_{i,j}\delta_{W_i}, \quad j=1,\dots,q.$$

As a simple CRM of Section S1, the multivariate extension of a CRM is characterized by its Lévy-Khintchine representation:

$$\begin{aligned} &\mathbb{E}[e^{-\int_{\mathbb{W}} f_1(w)\mu_1(\mathrm{d}w)-\cdots-\int_{\mathbb{W}} f_q(w)\mu_q(\mathrm{d}w)}] \\ &\quad = \exp\left\{-\int_{\mathbb{W}}\int_{\mathbb{R}_+^q} (1-e^{-s_1 f_1(w)-\cdots-s_q f_q(w)})\lambda_{(q)}(s_1,\dots,s_q)\mathrm{d}s_1\cdots\mathrm{d}s_q P(\mathrm{d}w)\right\} \end{aligned}$$

for arbitrary measurable functions $f_1, \ldots, f_d : \mathbb{W} \to \mathbb{R}_+$, where P is a probability measure on $\mathbb{W}$. The multivariate Lévy intensity $\lambda_{(q)}$ is assumed to satisfy the integral condition

$$\int_{\mathbb{R}_+^q} \min\{1, ||\boldsymbol{s}||\} \lambda_{(q)}(s_1, \ldots, s_q) \mathrm{d}s_1 \cdots \mathrm{d}s_q < +\infty$$

where $||\boldsymbol{s}||$ is the Euclidean norm of the vector $\boldsymbol{s}$. When $\lambda_{(q)}(s_1, \ldots, s_q)$ concentrates on S_q, the law of $\boldsymbol{\mu}$ may be employed as a distribution for the parameter $\boldsymbol{\zeta}$ of the simple multinomial process in (S27). A possible choice indicated by James [2017] is to select a stable-Beta-Dirichlet process, which is a generalization of the Beta-Dirichlet process [Kim et al., 2012] with power law behavior. We say that a multivariate CRM $\boldsymbol{\mu} = (\mu_1, \ldots, \mu_q)$ is a stable-Beta-Dirichlet process with parameters $(\alpha, \kappa + \alpha; \boldsymbol{\gamma}; \vartheta)$, where $\boldsymbol{\gamma} = (\gamma_1, \ldots, \gamma_q)$, if it is characterized by the following Lévy intensity specification

$$\lambda_{(q)}(\boldsymbol{s}) = \frac{\vartheta \Gamma(|\boldsymbol{\gamma}|)}{\prod_{j=1}^q \Gamma(\gamma_j)} |\boldsymbol{s}|^{-\alpha - |\boldsymbol{\gamma}|} (1 - \boldsymbol{s})^{\kappa + \alpha - 1} \prod_{j=1}^q s_j^{\gamma_j - 1} \mathbb{1}_{[0,1]}(|\boldsymbol{s}|), \ \boldsymbol{s} \in S_q \tag{S28}$$

where $0 \leq \alpha < 1, \kappa > -\alpha, \vartheta > 0$ and $\gamma_j > 0$ for any $j = 1, \ldots, q$. We write $\boldsymbol{\mu} \sim \mathrm{mSBD}(\alpha, \kappa + \alpha; \boldsymbol{\gamma}; \vartheta)$ to denote the distribution of the stable-Beta-Dirichlet process. As emphasized by James [2017], it can be easily checked, by means of the Laplace functional, that $\sum_{j=1}^q \mu_j$ is a stable-Beta process of Teh and Gorur [2009], i.e. a simple CRM on $\mathbb{W}$ with Lévy intensity on $[0, 1] \times \mathbb{W}$ equal to $\vartheta s^{-\alpha-1}(1 - s)^{\kappa+\alpha-1} \mathrm{d}s P(\mathrm{d}w)$.

S5.2.1 Estimation of the unseen features with a condiment

In order to face predictive inference with the model (S27) under the prior specification $\boldsymbol{\zeta} \sim \mathrm{mSBD}(\alpha, \kappa + \alpha; \boldsymbol{\gamma}; \vartheta)$, we need to characterize the predictive distribution of $\boldsymbol{Z}_{N+1} | \boldsymbol{Z}_{1:N}$ for the model (S27). To this end it is worth recalling the definition of the finite-dimensional Beta-Dirichlet distribution by Kim et al. [2012]. A random vector $\boldsymbol{P} := (P_1, \ldots, P_q)$ on S_q is said to follow a Beta-Dirichlet distribution with positive parameters α, κ and

$\boldsymbol{\gamma} = (\gamma_1, \ldots, \gamma_q)$ if the probability density function of the random vector $(P_1, \ldots, P_q)$ has density proportional to

$$|\boldsymbol{s}|^{\alpha-|\boldsymbol{\gamma}|} \cdot (1-|\boldsymbol{s}|)^{\kappa-1} \prod_{j=1}^{q} s_j^{\gamma_j - 1} \cdot \mathbb{1}_{S_q}(\boldsymbol{s}) \tag{S29}$$

and we write $(P_1, \ldots, P_q) \sim \mathscr{BD}(\alpha, \kappa; \boldsymbol{\gamma})$. This distribution can be characterized as follows: $|\boldsymbol{P}|$ has a Beta distribution with parameters (α, κ) and the normalized vector $(P_1/|\boldsymbol{P}|, \ldots, P_q/|\boldsymbol{P}|)$ follows a Dirichlet distribution with parameters $(\gamma_1, \ldots, \gamma_q)$.

We first characterize the distribution of $\boldsymbol{Z}_{N+1}|\boldsymbol{Z}_{1:N}$ under the prior specification $\boldsymbol{\zeta} \sim \mathrm{mSBD}(\alpha, \kappa+\alpha; \boldsymbol{\gamma}; \vartheta)$ in (S27). The following result is immediate from the theory developed by James [2017].

Theorem S1. *For any $N \geq 1$, let $\boldsymbol{Z}_{1:N}$ be a random sample modeled as the BNP multinomial process model (S27), with $\boldsymbol{\zeta} \sim \mathrm{mSBD}(\alpha, \kappa+\alpha; \boldsymbol{\gamma}; \vartheta)$. If $\boldsymbol{Z}_{1:N}$ displays $K_N = k$ distinct features, labeled by $W_1^*, \ldots, W_{K_N}^*$, with condiment-specific frequencies $(M_{N,1,j}, \ldots, M_{N,K_N,j}) = (m_{1,j}, \ldots, m_{k,j})$, for any $j = 1, \ldots, q$, then the conditional distribution of $\boldsymbol{Z}_{N+1}$, given $\boldsymbol{Z}_{1:N}$, coincides with the distribution of*

$$\boldsymbol{Z}_{N+1}|\boldsymbol{Z}_{1:N} \stackrel{d}{=} \boldsymbol{Z}'_{N+1} + \sum_{i=1}^{K_N} \boldsymbol{A}_{N+1,i}\delta_{W_i^*} \tag{S30}$$

where:

i) $\boldsymbol{Z}'_{N+1}$ *is such that* $\boldsymbol{Z}'_{N+1} = \sum_{i\geq 1} \boldsymbol{A}'_{N+1,i}\delta_{W'_i} \sim \mathsf{MP}(\boldsymbol{\mu}')$ *and* $\boldsymbol{\mu}' \sim \mathrm{mSBD}(\alpha, \kappa + M + \alpha; \boldsymbol{\gamma}; \vartheta)$*;*

ii) $\boldsymbol{A}_{N+1,1:K_N}$ *is a collection of independent simple multinomial random variables with respective parameters* $\boldsymbol{J}_{1:K_N}$*, such that each* $\boldsymbol{J}_i = (J_1, \ldots, J_q)$ *has a Beta-Dirichlet distribution, i.e.,* $\boldsymbol{J}_i \stackrel{ind}{\sim} \mathscr{BD}(m_i - \alpha, N - m_i + \kappa + \alpha; \boldsymbol{\gamma} + \boldsymbol{m}_i)$*, where we put* $\boldsymbol{m}_i := (m_{i,1}, \ldots, m_{i,q})$ *and* $m_i = \sum_{j=1}^{q} m_{i,j} = |\boldsymbol{m}_i|$ *for any* $i = 1, \ldots, K_N$*.*

Note that in Theorem S1 $M_{N,i,j}$ is the random number of times feature W_i^* has been observed out of $\boldsymbol{Z}_{1:N}$ with condiment $j \in \{1, \dots, q\}$, while $m_i = \sum_{j=1}^{q} m_{i,j}$ is the number of times feature W_i^* has been observed out of the sample.

For any $N \geq 1$, let $\boldsymbol{Z}_{1:N}$ be an observable sample modeled as the multinomial model in (S27), with $\boldsymbol{\zeta} \sim \text{mSBD}(\alpha, \kappa + \alpha; \boldsymbol{\gamma}; \vartheta)$. Moreover, under the same model, for $M \geq 1$ let $\boldsymbol{Z}_{N+1:N+M} = (\boldsymbol{Z}_{N+1}, \dots, \boldsymbol{Z}_{N+M})$ be an additional and unobserved sample. We now define the number of hitherto unobserved feature with condiment $\ell \in \{1, \dots, q\}$ that will be recorded out of $\boldsymbol{Z}_{N+1:N+M}$ as

$$U_{N,\ell}^{(M)} := \sum_{i \geq 1} \mathbb{1}\left(\sum_{m=1}^{M} A_{m,i,\ell} > 0 \right) \cdot \mathbb{1}\left(\sum_{n=1}^{N} A_{n,i,\ell} = 0 \right). \tag{S31}$$

Posterior inference for such a quantity could have potential interest in genomics to account for the presence of a variant with certain biological characteristics (condiment). The next theorem provides the posterior distribution of $U_{N,\ell}^{(M)}$.

Theorem S2. *For any $N \geq 1$, let $\boldsymbol{Z}_{1:N}$ be a random sample modeled as the BNP simple multinomial process model (S27), with $\boldsymbol{\zeta} \sim \text{mSBD}(\alpha, \kappa + \alpha; \boldsymbol{\gamma}; \vartheta)$. Suppose that $\boldsymbol{Z}_{1:N}$ displays $K_N = k$ distinct features, labeled by $W_1^*, \dots, W_{K_N}^*$, with condiment-specific frequencies $(M_{N,1,j}, \dots, M_{N,K_N,j}) = (m_{1,j}, \dots, m_{k,j})$, for any $j = 1, \dots, q$. Then, the posterior distribution of $U_{N,\ell}^{(M)}$, given $\boldsymbol{Z}_{1:N}$, coincides with the distribution of*

$$U_{N,\ell}^{(M)} | \boldsymbol{Z}_{1:N} \sim \text{Poisson}\left(\vartheta \sum_{m=1}^{M} (-1)^{m+1} \binom{M}{m} B(m - \alpha, N + \alpha + \kappa) \frac{(\gamma_\ell)_m}{(|\boldsymbol{\gamma}|)_m} \right) \tag{S32}$$

Proof. The proof is based on the posterior characterization provided in Theorem S1 and the evaluation of the probability generating function of the random variable $U_{N,\ell}^{(M)}$, conditionally on the sample $\boldsymbol{Z}_{1:N}$. The probability generating function is denoted as usual by $\mathcal{G}_{U_{N,\ell}^{(M)}}(\,\cdot\,)$. Thanks to the characterization (S30), conditionally on $\boldsymbol{Z}_{1:N}$, the random variable $U_{N,\ell}^{(M)}$

may be written as

$$U_{N,\ell}^{(M)} | \boldsymbol{Z}_{1:N} \stackrel{d}{=} \sum_{i\geq 1} \mathbb{1}\left(\sum_{m=1}^{M} A'_{m+N,i,\ell} > 0\right).$$

Fix t in a neighborhood of the origin, then one has

$$\mathcal{G}_{U_{N,\ell}^{(M)}}(t) = \mathbb{E}\left[t^{U_{N,\ell}^{(M)}} \mid \boldsymbol{Z}_{1:N}\right]. \tag{S33}$$

Here, independently across i, $A'_{N+m,i,\ell}$ is a Bernoulli random variable with parameter $\rho'_{i,\ell}$, conditionally on the random measure $\boldsymbol{\mu}' = \sum_{i\geq 1} \boldsymbol{\rho}'_i \delta_{W'_i}$ with Lévy intensity $\lambda'_{(q)}(\boldsymbol{s})\mathrm{d}s_1 \cdots \mathrm{d}s_q P(\mathrm{d}w)$ such that

$$\lambda'_{(q)}(\boldsymbol{s}) = \frac{\vartheta\Gamma(|\boldsymbol{\gamma}|)}{\prod_{j=1}^{q}\Gamma(\gamma_j)} |\boldsymbol{s}|^{-\alpha-|\boldsymbol{\gamma}|}(1-\boldsymbol{s})^{N+\kappa+\alpha-1} \prod_{j=1}^{q} s_j^{\gamma_j-1} \mathbb{1}_{[0,1]}(|\boldsymbol{s}|),\ \boldsymbol{s} \in S_q. \tag{S34}$$

Thus, the expected value in (S33) boils down to

$$\begin{aligned}
\mathcal{G}_{U_{N,\ell}^{(M)}}(t) &= \mathbb{E}\left[t^{\sum_{i\geq 1}\mathbb{1}\left(\sum_{m=1}^{M} A'_{m+N,i,\ell}>0\right)}\right] = \mathbb{E}\left[\prod_{i\geq 1}\mathbb{E}\left[t^{\mathbb{1}\left(\sum_{m=1}^{M} A'_{m+N,i,\ell}>0\right)} \mid \boldsymbol{\mu}'\right]\right] \\
&= \mathbb{E}\left[\prod_{i\geq 1}\left(t + (1-t)\prod_{m=1}^{M}\mathbb{P}(A'_{m+N,i,\ell} = 0|\boldsymbol{\mu}')\right)\right] \\
&= \mathbb{E}\left[\prod_{i\geq 1}(t + (1-t)(1-\rho'_{i,\ell})^M)\right].
\end{aligned}$$

where we used the fact that each $A'_{m+N,i,\ell}$ is a Bernoulli random variable with parameter $\rho'_{i,\ell}$, conditionally on the random measure $\boldsymbol{\mu}'$, and in addition these random variables are conditionally independent. We now exploit the Laplace functional of the multivariate CRM $\boldsymbol{\mu}'$ to obtain

$$\begin{aligned}
\mathcal{G}_{U_{N,\ell}^{(M)}}(t) &= \mathbb{E}\left[\exp\left\{\sum_{i\geq 1}\log(t + (1-t)(1-\rho'_{i,\ell})^M)\right\}\right] \\
&= \exp\left\{-(1-t)\int_{S_q}[1-(1-s_\ell)^M]\lambda'_{(q)}(\boldsymbol{s})\mathrm{d}s_1\cdots\mathrm{d}s_q\right\} \\
&= \exp\left\{(1-t)\sum_{m=1}^{M}(-1)^m\binom{M}{m}\int_{S_q} s_\ell^m \lambda'_{(q)}(\boldsymbol{s})\mathrm{d}s_1\cdots\mathrm{d}s_q\right\}
\end{aligned} \tag{S35}$$

where $\lambda'_{(q)}$ has been specified in (S34) and we exploited the following formula

$$[1-(1-s_\ell)^M] = 1 - \sum_{m=0}^{M} (-1)^m \binom{M}{m} s_\ell^m = -\sum_{m=1}^{M} (-1)^m \binom{M}{m} s_\ell^m. \tag{S36}$$

The integrals over S_q in (S35) may be easily evaluated (see, e.g., [Gradshteyn and Ryzhik, 2007, Formula 4.635.2]) to get

$$\int_{S_q} s_\ell^m \lambda'_{(q)}(\boldsymbol{s}) \mathrm{d}s_1 \cdots \mathrm{d}s_q = \vartheta \frac{(\gamma_\ell)_m}{(|\boldsymbol{\gamma}|)_m} \cdot B(m-\alpha, N+\alpha+\kappa).$$

By substituting the previous expression in (S35), we obtain

$$\mathcal{G}_{U_{N,\ell}^{(M)}}(t) = \exp\left\{ (t-1) \sum_{m=1}^{M} (-1)^{m+1} \binom{M}{m} \vartheta \frac{(\gamma_\ell)_m}{(|\boldsymbol{\gamma}|)_m} \cdot B(m-\alpha, N+\alpha+\kappa) \right\}$$

which is exactly the probability generating function of a Poisson random variable with parameter

$$\sum_{m=1}^{M} (-1)^{m+1} \binom{M}{m} \vartheta \frac{(\gamma_\ell)_m}{(|\boldsymbol{\gamma}|)_m} \cdot B(m-\alpha, N+\alpha+\kappa).$$

□

As a consequence of Theorem S2, one can define a BNP estimator of $U_{N,\ell}^{(M)}$ with respect to a squared loss function as follows:

$$\hat{U}_{N,\ell}^{(M)} = \vartheta \sum_{m=1}^{M} (-1)^{m+1} \binom{M}{m} B(m-\alpha, N+\alpha+\kappa) \frac{(\gamma_\ell)_m}{(|\boldsymbol{\gamma}|)_m}. \tag{S37}$$

We point out that for computational convenience one may write

$$\hat{U}_{N,\ell}^{(M)} = \vartheta B(1-\alpha, N+\alpha+\kappa) \mathbb{E}_{(X,Y)} \left[\frac{1-(1-XY)^M}{Y} \right] \tag{S38}$$

where the expected value is taken with respect to the two independent random variables with the following beta distributions

$$X \sim \mathrm{Beta}(\gamma_\ell, |\boldsymbol{\gamma}| - \gamma_\ell), \quad Y \sim \mathrm{Beta}(1-\alpha, N+\alpha+\kappa).$$

The equality (S38) may be easily proved by observing that

$$\mathbb{E}_X[X^m] = \frac{(\gamma_\ell)_m}{(|\boldsymbol{\gamma}|)_m} \quad \text{and} \quad B(m-\alpha, N+\alpha+\kappa) = \mathbb{E}_Y[Y^{m-1}] B(1-\alpha, N+\alpha+\kappa).$$

S5.3 Scaled stable-Beta-Dirichlet prior for multinomial processes

From Theorems S1-S2, it is apparent that, under the stable-Beta-Dirichlet process, the conditional distribution of a statistic involving hitherto unobserved features, depends on the initial sample $\boldsymbol{Z}_{1:N}$ only trough the sample size N and not on other sample statistics. This behavior resembles what happens for the Bernoulli process model described in the main paper when the prior ζ in (1) is a CRM. We then introduce a multivariate analogue of the stable-Beta scaled prior, that will be termed *scaled stable-Beta-Dirichlet process* with the goal to enrich the predictive structure. We introduce a discrete random measure depending on the random jump $\Delta_{1,h_{c,\beta}}$, that has been defined in the main paper as a polynomial-exponential tilting of the density function (6), whose density equals

$$f_{\Delta_{1,h_{c,\beta}}}(a) = \frac{\sigma\beta^{c+1}}{\Gamma(c+1)} a^{-\sigma(c+1)-1} \exp\left\{-\beta a^{-\sigma}\right\} \mathbb{1}_{\mathbb{R}_+}(a) \tag{S39}$$

as shown in (S17). The scaled stable-Beta-Dirichlet random measure is an almost surely discrete random measure that can be represented as

$$\boldsymbol{\mu}_{\Delta_{1,h_{c,\beta}}} = \sum_{i\geq 1} \boldsymbol{\rho}_i \delta_{W_i}, \quad \boldsymbol{\rho}_i = (\rho_{i,1}, \ldots, \rho_{i,q})$$

and consisting of q components

$$\mu_{\Delta_{1,h_{c,\beta}},j} = \sum_{i\geq 1} \rho_{i,j}\delta_{W_i} \quad \text{as } j = 1, \ldots, q.$$

Conditionally on the jump $\Delta_{1,h_{c,\beta}}$, the multivariate random measure $\boldsymbol{\mu}_{\Delta_{1,h_{c,\beta}}}$ is completely random with Lévy intensity $\lambda_{(q),\Delta_{1,h_{c,\beta}}}(\boldsymbol{s})\mathrm{d}s_1\cdots\mathrm{d}s_q P(\mathrm{d}p)$ with the specification

$$\lambda_{(q),\Delta_{1,h_{c,\beta}}}(\boldsymbol{s}) = \frac{\Gamma(|\boldsymbol{\gamma}|)}{\prod_{j=1}^q \Gamma(\gamma_j)} \sigma \Delta_{1,h_{c,\beta}}^{-\sigma} |\boldsymbol{s}|^{-\sigma-|\gamma|} \prod_{j=1}^q s_j^{\gamma_j - 1} \mathbb{1}_{[0,1]}(|\boldsymbol{s}|), \; \boldsymbol{s} \in S_q \tag{S40}$$

where $0 < \sigma < 1$ and $\gamma_j > 0$ for any $j = 1, \ldots, q$. We write $\boldsymbol{\mu}_{\Delta_{1,h}} \sim$ S-mSBD$(\sigma, \boldsymbol{\gamma}; h_{c,\beta})$. A remarkable property of this model is that $\sum_{j=1}^q \mu_{\Delta_{1,h_{c,\beta}},j}$ is distributed as the stable-Beta scaled process prior, i.e., $|\boldsymbol{\mu}_{\Delta_{1,h_{c,\beta}}}| \sim$ SB-SP(σ, c, β). Such a property may be easily

proved by means of the Laplace functionals. Note that one could potentially introduce an additional mass parameter in the model, but this is irrelevant to carry out posterior inference in the stable case.

S5.3.1 Posterior Analysis

We now provide posterior, predictive and marginal characterizations for the multivariate model (S27) under the scaled stable-Beta-Dirichlet process prior specification for $\mathscr{Z}$. The results we present here may be proved by exploiting [James, 2017, Section 5], conditionally on $\Delta_{1,h_{c,\beta}}$ and then by marginalizing over the mixing distribution (S39). We omit the details.

Theorem S3. *For any $N \geq 1$, let $\boldsymbol{Z}_{1:N}$ be a random sample modeled as the BNP simple multinomial process model (S27), with $\boldsymbol{\zeta} \sim$ S-mSBD$(\sigma, \boldsymbol{\gamma}; h_{c,\beta})$. If $\boldsymbol{Z}_{1:N}$ displays $K_N = k$ distinct features, labeled by $W_1^*, \dots, W_{K_N}^*$, with condiment-specific frequencies $(M_{N,1,j}, \dots, M_{N,K_N,j}) = (m_{1,j}, \dots, m_{k,j})$, for any $j = 1, \dots, q$, then the conditional distribution of $\Delta_{1,h_{c,\beta}}$ given $\boldsymbol{Z}_{1:N}$, coincides with the distribution of*

$$\Delta_{1,h_{c,\beta}}^{-\sigma} \sim \text{Gamma}(K_N + c + 1, \beta + \gamma_0^{(N)}) \tag{S41}$$

where $\gamma_0^{(n)} = \sigma \sum_{1 \leq i \leq n} B(1 - \sigma, i)$. Moreover, the conditional distribution of $\boldsymbol{\zeta}$, given $\boldsymbol{Z}_{1:N}, \Delta_{1,h_{c,\beta}}$, coincides with the distribution of

$$\boldsymbol{\zeta} | (\boldsymbol{Z}_{1:N}, \Delta_{1,h_{c,\beta}}) \stackrel{d}{=} \boldsymbol{\mu}'_{\Delta_{1,h_{c,\beta}}} + \sum_{i=1}^{K_N} \boldsymbol{J}_i \delta_{W_i^*} \tag{S42}$$

where:

i) $\boldsymbol{\mu}'_{\Delta_{1,h_{c,\beta}}}$ is a discrete multivariate random measure with Lévy intensity

$$\begin{aligned} \nu'_{\Delta_{1,h_{c,\beta}}}(\mathrm{d}s_1, \dots, \mathrm{d}s_q, \mathrm{d}w) = & \frac{\Gamma(|\boldsymbol{\gamma}|)}{\prod_{j=1}^q \Gamma(\gamma_j)} \\ & \times |\boldsymbol{s}|^{-\sigma - |\boldsymbol{\gamma}|}(1 - \boldsymbol{s})^N \prod_{j=1}^q s_j^{\gamma_j - 1} \mathbb{1}_{[0,1]}(|\boldsymbol{s}|) \sigma \Delta_{1,h_{c,\beta}}^{-\sigma} \mathrm{d}s_1 \cdots \mathrm{d}s_q \, P(\mathrm{d}w); \end{aligned} \tag{S43}$$

ii) $\boldsymbol{J}_{1:K_N}$ *is a vector of independent random jumps such that each* $\boldsymbol{J}_i = (J_1, \ldots, J_q)$ *has a Beta-Dirichlet distribution, i.e.,*

$$\boldsymbol{J}_i | \Delta_{1,h_{c,\beta}} \sim \mathscr{BD}(m_i - \sigma, N - m_i + 1; \boldsymbol{\gamma} + \boldsymbol{m}_i) \tag{S44}$$

where we put $\boldsymbol{m}_i := (m_{i,1}, \ldots, m_{i,q})$ *and* $m_i = \sum_{j=1}^q m_{i,j} = |\boldsymbol{m}_i|$ *for any* $i = 1, \ldots, K_N$.

Theorem S4. *For any* $N \geq 1$*, let* $\boldsymbol{Z}_{1:N}$ *be a random sample modeled as the BNP simple multinomial process model (S27), with* $\boldsymbol{\zeta} \sim \text{S-mSBD}(\sigma, \boldsymbol{\gamma}; h_{c,\beta})$*. If* $\boldsymbol{Z}_{1:N}$ *displays* $K_N = k$ *distinct features, labeled by* $W_1^*, \ldots, W_{K_N}^*$*, with condiment-specific frequencies* $(M_{N,1,j}, \ldots, M_{N,K_N,j}) = (m_{1,j}, \ldots, m_{k,j})$*, for any* $j = 1, \ldots, q$*, then the conditional distribution of* $\Delta_{1,h_{c,\beta}}$ *given* $\boldsymbol{Z}_{1:N}$*, coincides with (S41). Moreover, the conditional distribution of* $\boldsymbol{Z}_{N+1}$*, given* $\boldsymbol{Z}_{1:N}, \Delta_{1,h_{c,\beta}}$*, coincides with the distribution of*

$$\boldsymbol{Z}_{N+1} | (\boldsymbol{Z}_{1:N}, \Delta_{1,h_{c,\beta}}) \stackrel{d}{=} \boldsymbol{Z}'_{N+1} + \sum_{i=1}^{K_N} \boldsymbol{A}_{N+1,i} \delta_{W_i^*} \tag{S45}$$

where:

i) $\boldsymbol{Z}'_{N+1}$ *is such that* $\boldsymbol{Z}'_{N+1} | \Delta_{1,h_{c,\beta}} = \sum_{i \geq 1} \boldsymbol{A}'_{N+1,i} \delta_{W'_i} \sim \mathsf{MP}(\boldsymbol{\mu}'_{\Delta_{1,h_{c,\beta}}})$ *and* $\boldsymbol{\mu}'_{\Delta_{1,h_{c,\beta}}} | \Delta_{1,h_{c,\beta}}$ *is the completely random measure having the Lévy intensity (S43);*

ii) $\boldsymbol{A}_{N+1,1:K_N}$ *is a collection of independent simple multinomial random variables with parameters* $\boldsymbol{J}_{1:K_N}$*, each one distributed according to Equation (S44).*

Theorem S5. *For any* $N \geq 1$*, let* $\boldsymbol{Z}_{1:N}$ *be a random sample modeled as the BNP simple multinomial process model (S27), with* $\boldsymbol{\zeta} \sim \text{S-mSBD}(\sigma, \boldsymbol{\gamma}; h_{c,\beta})$*. The probability that* $\boldsymbol{Z}_{1:N}$ *displays a particular feature allocation of* $K_N = k$ *distinct features with condiment-specific*

frequencies $(M_{N,1,j},\dots,M_{N,K_N,j})=(m_{1,j},\dots,m_{k,j})$*, for any* $j=1,\dots,q$*, equals*

$$\begin{aligned}p_k^{(N)}(\boldsymbol{m}_1,\dots,\boldsymbol{m}_k)=\prod_{i=1}^{k}\left\{B(m_i-\sigma,N-m_i+1)\frac{\prod_{j=1}^{q}(\gamma_j)_{m_{i,j}}}{(|\boldsymbol{\gamma}|)_{m_i}}\right\}\\ \times\frac{\Gamma(k+c+1)}{\Gamma(c+1)}\cdot\frac{\sigma^k\beta^{c+1}}{(\beta+\gamma_0^{(N)})^{k+c+1}}.\end{aligned}\tag{S46}$$

S5.3.2 Estimation of the unseen features with a condiment

For any $N\geq 1$, let $\boldsymbol{Z}_{1:N}$ be an observable sample modeled as the simple multinomial model in (S27), with $\boldsymbol{\zeta}\sim\text{S-mSBD}(\sigma,\boldsymbol{\gamma};h_{c,\beta})$. Moreover, under the same model, for $M\geq 1$ let $\boldsymbol{Z}_{N+1:N+M}=(\boldsymbol{Z}_{N+1},\dots,\boldsymbol{Z}_{N+M})$ be an additional and unobserved sample. Under this model, we now determine the posterior distribution of the sample statistic $U_{N,\ell}^{(M)}$ in (S31), counting the number of hitherto unobserved feature with condiment $\ell\in\{1,\dots,q\}$ that will be recorded out of the additional sample.

Theorem S6. *For any* $N\geq 1$*, let* $\boldsymbol{Z}_{1:N}$ *be a random sample modeled as the BNP simple multinomial process model* (S27)*, with* $\boldsymbol{\zeta}\sim\text{S-mSBD}(\sigma,\boldsymbol{\gamma};h_{c,\beta})$*. Suppose that* $\boldsymbol{Z}_{1:N}$ *displays* $K_N=k$ *distinct features with condiment-specific frequencies* $(M_{N,1,j},\dots,M_{N,K_N,j})=(m_{1,j},\dots,m_{k,j})$*, for any* $j=1,\dots,q$*. Then, the posterior distribution of* $U_{N,\ell}^{(M)}$*, given* $\boldsymbol{Z}_{1:N}$*, coincides with the distribution of*

$$U_{N,\ell}^{(M)}|\boldsymbol{Z}_{1:N}\sim\text{NegativeBinomial}\left(K_N+c+1,\frac{\psi_{N,\ell}^{(M)}}{\psi_{N,\ell}^{(M)}+\gamma_0^{(N)}+\beta}\right)\tag{S47}$$

where we defined

$$\psi_{N,\ell}^{(M)}:=\sigma\sum_{m=1}^{M}\binom{M}{m}(-1)^{m+1}\frac{(\gamma_\ell)_m}{(|\boldsymbol{\gamma}|)_m}B(m-\sigma,N+1).$$

Proof. The proof is based on the posterior characterization in Theorem S3 and on Theorem S4. As in the proof of Theorem S2 we evaluate the probability generating function of the random variable $U_{N,\ell}^{(M)}$, conditionally on the sample $\boldsymbol{Z}_{1:N}$. The probability generating function is denoted as usual by $\mathcal{G}_{U_{N,j}^{(M)}}(\,\cdot\,)$. Thanks to the characterization (S45), conditionally

on $\boldsymbol{Z}_{1:N}, \Delta_{1,h_{c,\beta}}$, the random variable $U_{N,\ell}^{(M)}$ may be written as

$$U_{N,\ell}^{(M)}|(\boldsymbol{Z}_{1:N}, \Delta_{1,h_{c,\beta}}) \stackrel{d}{=} \sum_{i\geq 1} \mathbb{1}\left(\sum_{m=1}^{M} A'_{m+N,i,\ell} > 0\right).$$

Fix t in a neighborhood of the origin, then one has

$$\mathcal{G}_{U_{N,\ell}^{(M)}}(t) = \mathbb{E}\left[t^{U_{N,\ell}^{(M)}} \mid \boldsymbol{Z}_{1:N}\right] = \mathbb{E}\left[\mathbb{E}\left[t^{U_{N,\ell}^{(M)}} \mid \boldsymbol{Z}_{1:N}, \Delta_{1,h_{c,\beta}}\right] \mid \boldsymbol{Z}_{1:N}\right] \tag{S48}$$

by an application of the tower property. We now focus on the evaluation of the inner expected value in (S48):

$$\begin{aligned}\mathbb{E}\left[t^{U_{N,\ell}^{(M)}} \mid \boldsymbol{Z}_{1:N}, \Delta_{1,h_{c,\beta}}\right] &= \mathbb{E}\left[t^{\sum_{m=1}^{M} A'_{m+N,i,\ell}}\right] \\ &= \mathbb{E}\left[\prod_{i\geq 1} \mathbb{E}[1 \cdot \mathbb{P}(\sum_{m=1}^{M} A'_{m+N,i,\ell} = 0) + t \cdot \mathbb{P}(\sum_{m=1}^{M} A'_{m+N,i,\ell} > 0)]\right].\end{aligned}$$

From Theorem S4, the $A'_{m+N,i,\ell}$s are independent random variables as $m = 1, \ldots, M$, and each one $A'_{N+m,i,\ell}$ is a Bernoulli with parameter $\rho'_{i,\ell}$, conditionally on the random measure $\boldsymbol{\mu}'_{\Delta_{1,h_{c,\beta}}} = \sum_{i\geq 1} \boldsymbol{\rho}'_i \delta_{W'_i}$ with Lévy intensity (S43). As a consequence we obtain

$$\mathbb{E}\left[t^{U_{N,\ell}^{(M)}} \mid \boldsymbol{Z}_{1:N}, \Delta_{1,h_{c,\beta}}\right] = \mathbb{E}\left[\prod_{i\geq 1}\left[t + (1-t)(1-\rho'_{i,\ell})^M\right]\right].$$

Proceeding along the same lines as in the proof of Theorem S2 we have that

$$\begin{aligned}\mathbb{E}\left[t^{U_{N,\ell}^{(M)}} \mid \boldsymbol{Z}_{1:N}, \Delta_{1,h_{c,\beta}}\right] &= \mathbb{E}\left[\exp\left\{\sum_{i\geq 1} \log(t + (1-t)(1-\rho_{i,\ell})^M)\right\}\right] \\ &= \exp\left\{-(1-t)\int_{\mathbb{W}}\int_{S_q} [1-(1-s_\ell)^M]\nu'_{\Delta_{1,h_{c,\beta}}}(\mathrm{d}s_1, \ldots, \mathrm{d}s_q, \mathrm{d}w)\right\}.\end{aligned}$$

Now define

$$\lambda'_{(q),\Delta_{1,h_{c,\beta}}}(\boldsymbol{s}) := \frac{\Gamma(|\boldsymbol{\gamma}|)}{\prod_{j=1}^{q}\Gamma(\gamma_j)} |\boldsymbol{s}|^{-\sigma-|\boldsymbol{\gamma}|}(1-\boldsymbol{s})^N \prod_{j=1}^{q} s_j^{\gamma_j - 1} \mathbb{1}_{[0,1]}(|\boldsymbol{s}|)\sigma\Delta_{1,h_{c,\beta}}^{-\sigma}$$

thus, the conditional expected value under study may be written as

$$\begin{aligned}\mathbb{E}\left[t^{U_{N,\ell}^{(M)}} \mid \boldsymbol{Z}_{1:N}, \Delta_{1,h_{c,\beta}}\right] &= \exp\left\{-(1-t)\int_{S_q}[1-(1-s_\ell)^M]\lambda'_{(q),\Delta_{1,h_{c,\beta}}}(\boldsymbol{s})\mathrm{d}s_1, \ldots, \mathrm{d}s_q\right\} \\ &= \exp\left\{-(1-t)\sum_{m=1}^{M}\binom{M}{m}(-1)^{m+1}\int_{S_q} s_\ell^m \lambda'_{(q),\Delta_{1,h_{c,\beta}}}(\boldsymbol{s})\mathrm{d}s_1, \ldots, \mathrm{d}s_q\right\}\end{aligned} \tag{S49}$$

where we applied (S36). The integral over S_q appearing in (S49) may be evaluated resorting to [Gradshteyn and Ryzhik, 2007, Formula 4.635.2], therefore

$$\begin{aligned}
&\int_{S_q} s_\ell^m \lambda'_{(q),\Delta_{1,h_{c,\beta}}}(\boldsymbol{s}) \mathrm{d}s_1, \dots, \mathrm{d}s_q \\
&\qquad = \sigma \Delta_{1,h_{c,\beta}}^{-\sigma} \frac{\Gamma(|\boldsymbol{\gamma}|)}{\prod_{j=1}^q \Gamma(\gamma_j)} \int_{S_q} s_\ell^m (1-|\boldsymbol{s}|)^N |\boldsymbol{s}|^{-\sigma-|\boldsymbol{\gamma}|} \prod_{j=1}^q s_j^{\gamma_j - 1} \mathrm{d}s_1 \cdots \mathrm{d}s_q \\
&\qquad = \sigma \Delta_{1,h_{c,\beta}}^{-\sigma} \frac{(\gamma_\ell)_m}{(|\boldsymbol{\gamma}|)_m} B(m-\sigma, N+1).
\end{aligned}$$

Thus, by substituting the previous expression in (S49) one obtains

$$\mathbb{E}\left[t^{U_{N,\ell}^{(M)}} \mid \boldsymbol{Z}_{1:N}, \Delta_{1,h_{c,\beta}} \right] = \exp\left\{ -(1-t) \Delta_{1,h_{c,\beta}}^{-\sigma} \psi_{N,\ell}^{(M)} \right\} \tag{S50}$$

where we recall that $\psi_{N,\ell}^{(M)}$ has been defined as follows

$$\psi_{N,\ell}^{(M)} = \sigma \sum_{m=1}^{M} \binom{M}{m} (-1)^{m+1} \frac{(\gamma_\ell)_m}{(|\boldsymbol{\gamma}|)_m} B(m-\sigma, N+1).$$

As a consequence, the probability generating function in (S48) equals

$$\mathcal{G}_{U_{N,\ell}^{(M)}}(t) \overset{(S50)}{=} \mathbb{E}\left[\exp\left\{ -(1-t) \Delta_{1,h_{c,\beta}}^{-\sigma} \psi_{N,\ell}^{(M)} \right\} \mid \boldsymbol{Z}_{1:N} \right].$$

The conclusion follows by a marginalization w.r.t. the posterior distribution of $\Delta_{1,h_{c,\beta}}^{-\sigma}$ which is a gamma random variable (see (S41)):

$$\begin{aligned}
\mathcal{G}_{U_{N,\ell}^{(M)}}(t) &= \int_0^\infty e^{-(1-t)\psi_{N,\ell}^{(M)} x} \cdot \frac{(\beta + \gamma_0^{(N)})^{K_N + c + 1}}{\Gamma(K_N + c + 1)} x^{K_N + c} e^{-x(\gamma_0^{(N)} + \beta)} \mathrm{d}x \\
&= \frac{(\beta + \gamma_0^{(N)})^{K_N + c + 1}}{\Gamma(K_N + c + 1)} \int_0^\infty e^{-[(\gamma_0^{(N)} + \beta) + (1-t)\psi_{N,\ell}^{(M)}]x} x^{K_N + c + 1 - 1} \mathrm{d}x \\
&= \frac{(\beta + \gamma_0^{(N)})^{K_N + c + 1}}{[(\gamma_0^{(N)} + \beta) + (1-t)\psi_{N,\ell}^{(M)}]^{K_N + c + 1}} \\
&= \left(\frac{\beta + \gamma_0^{(N)}}{\gamma_0^{(N)} + \beta + \psi_{N,\ell}^{(M)} - t\psi_{N,\ell}^{(M)}} \right)^{K_N + c + 1}
\end{aligned}$$

which is the probability generating function of a negative binomial distribution as in the statement. □

As a consequence of Theorem S6, the BNP estimator of $U_{N,\ell}^{(M)}$ under a squared loss function equals

$$\hat{U}_{N,\ell}^{(M)} = (K_N + c + 1)\frac{\psi_{N,\ell}^{(M)}}{\gamma_0^{(N)} + \beta}. \tag{S51}$$

For computational purposes, we finally note that the parameter $\psi_{N,\ell}^{(M)}$ in the posterior representations may be computed as

$$\psi_{N,\ell}^{(M)} = B(1-\sigma, N+1)\mathbb{E}_{(X,Y)}\left[\frac{1-(1-XY)^M}{Y}\right]$$

where the expected value is made w.r.t. two independent random variables X and Y having beta distributions as follows

$$X \sim \text{Beta}(\gamma_\ell, |\boldsymbol{\gamma}| - \gamma_\ell) \quad \text{and} \quad Y \sim \text{Beta}(1-\sigma, N+1).$$

S6 Synthetic experiments from the model

We now analyze empirically the properties of the SB-SP-Bernoulli model used in Section 3. We will use the acronym SSB for brevity in the captions. I.e., we consider the hierarchical model detailed in (1), with $\mu \sim \text{SB-SP}(\sigma, c, \beta)$. The predictive characterization detailed in Proposition 3, together with Equation (13), provides an algorithm to sample N observations from the model: given $\beta > 0, \sigma \in (0,1), c > 0$,

- at every step $n = 1, \ldots, N$, conditionally on the previous $n-1$ samples $Z_{1:n-1}$ showing K_{n-1} distinct features, each feature $k = 1, \ldots, m_{K_{n-1}}$ with frequency m_k, sample
  - a random number of new features observed:

$$U_{n-1}^{(1)} \mid Z_{1:n-1} \sim \text{NegativeBinonial}\left(K_{n-1} + c + 1, \frac{\gamma_{n-1}^{(1)}}{\beta + \gamma_0^{(n)}}\right);$$

  - for previously observed feature $i = 1, \ldots, K_{n-1}$:

$$A_{n,i} \mid Z_{1:n-1} \sim \text{Bernoulli}\left(m_i - \sigma, N - m_i + 1\right);$$

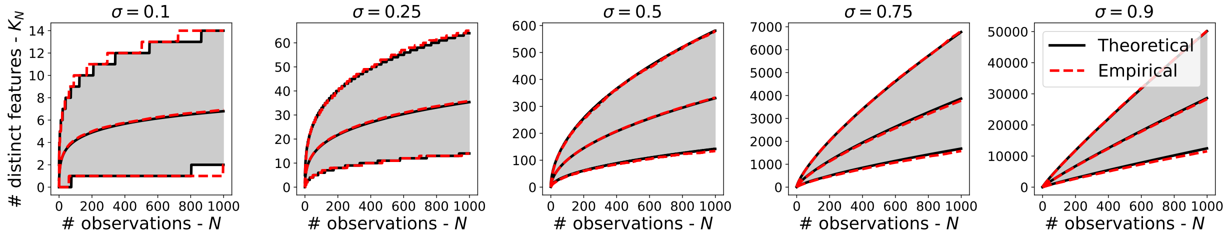

Figure S1: 90% centered credible interval for the number of distinct features K_N (y-axis) as a function of the sample size N (x-axis). We fix $\beta = 1$, $c = 5$, and vary σ across subplots. For the $5\%, 50\%, 95\%$ quantiles, we compare the theoretical value (solid black lines) to empirical result (dashed red lines), obtained by drawing $N_{MC} = 1000$ different datasets with the same parameter specification.

In particular, for the first step, $K_0 = 0$.

S6.1 Predictive behavior of the number of new features from the prior

First, we investigate the predictive behavior of the model as we vary the hyperparameters of the process — β, σ, c. Because our interest is in understanding the coverage properties of the posterior predictive distribution induced by the model, we report, together with the predictive mean, also posterior predictive credible intervals. In this first set of simulations reported in Sections S6.1.1 to S6.1.3, we assume the hyperparameter β, σ, c to be known.

S6.1.1 The role of σ

We start by analyzing the role of σ in Figure S1. As suggested by the asymptotic behavior analyzed in Theorem 3, σ directly controls the asymptotic rate of growth of the number of distinct features: as σ increases, the expected number of variants increases, approaching a linear behavior as $\sigma \to 1$. We notice that this behavior is reminiscent of the tail parameter of the stable beta-Bernoulli process [Teh and Gorur, 2009, Broderick et al., 2012].

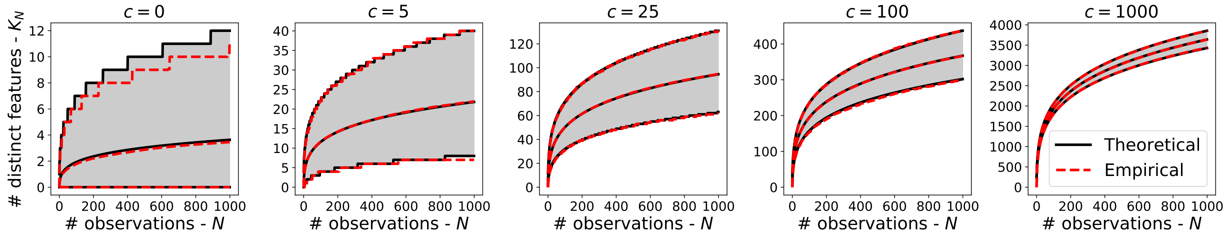

Figure S2: 90% centered credible interval for the number of distinct features K_N (y-axis) as a function of the sample size N (x-axis). We repeat the same experiments as in Figure S1, but now fix $\beta = 1$, $\sigma = 0.2$, and vary c across subplots.

S6.1.2 The role of c

We now move to the analysis of the polynomial tilting parameter, c. As suggested by the predictive distribution given in Equation (13), c acts as a "prior" number of features. That is, in the prior, the expected number of features to be observed from N samples is a Negative Binomial random variable with parameters $c + 1, \gamma_0^{(N)}/(\beta + \gamma_0^{(N)})$, i.e. with expectation given by

$$\mathbb{E}[U_0^N] = (c+1)\left(\frac{\gamma_0^{(N)}}{\beta}\right).$$

Again, larger values of c induce a higher rate of growth in the number of features, as showed in Figure S2.

S6.1.3 The role of β

Last, we analyze the role of the exponential tilting parameter, β. Inspecting again the predictive distribution Equation (13), β affects the number of new variants thorugh the success probability of the negative binomial — for fixed $c, \sigma, N, M, Z_{1:N}$, the expected number of new variants $U_N^{(M)} \mid Z_{1:N}$ depends inversely on the parameter β. We verify this empirically in Figure S3.

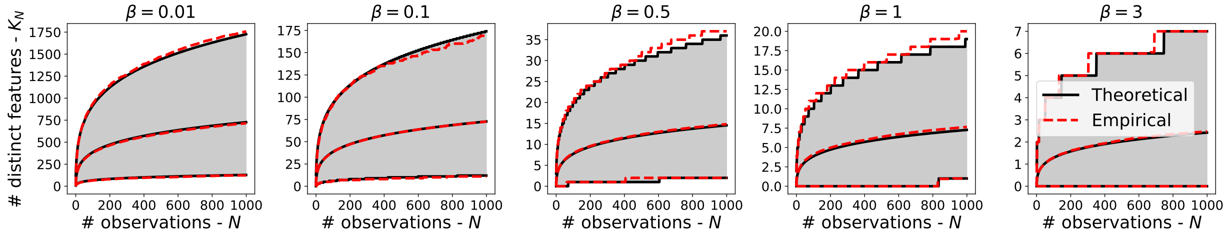

Figure S3: 90% centered credible interval for the number of distinct features K_N (y-axis) as a function of the sample size N (x-axis). We repeat the same exercise as in Figures S1-S2 but now $c = 1$, $\sigma = 0.2$, and vary β across subplots.

S6.2 Predictive behavior of the number of new features from the posterior

Next, we perform a slightly different exercise from the one described above. We still assume the parameters to be known, and we investigate how the posterior predictive behavior varies as we change the number of training samples N with respect to a total sampling "capacity" L. Intuitively, for a fixed value of this "sampling capacity", $N + M = L$, the expected number of observed features from the model should be the independent of the choice of N, M. However, we expect the distribution (e.g., the posterior variance), to concentrate as N increases relative to M. To perform this experiment, we do as follow: we fix β, c, σ and, for each $\ell = 1, \ldots, 2000$, we let $K_\ell = U_0^{(\ell)}$. Next, for $N \in \{50, 100, 500, 1000\}$, we compute $U_N^{(M)} \mid Z_{1:N}$, where we condition on the number of observed variants as given by the curve $\{K_\ell\}_{\ell=1,\ldots,2000}$. As displayed in Figure S4 and Figure S5, the width of the credible intervals shrinks with increasing training sizes N.

S6.3 Estimation of the parameters

Next, we move to the more interesting scenario in which the parameters are unknown and need to be inferred from the data. The natural way to estimate the unknown parameters is

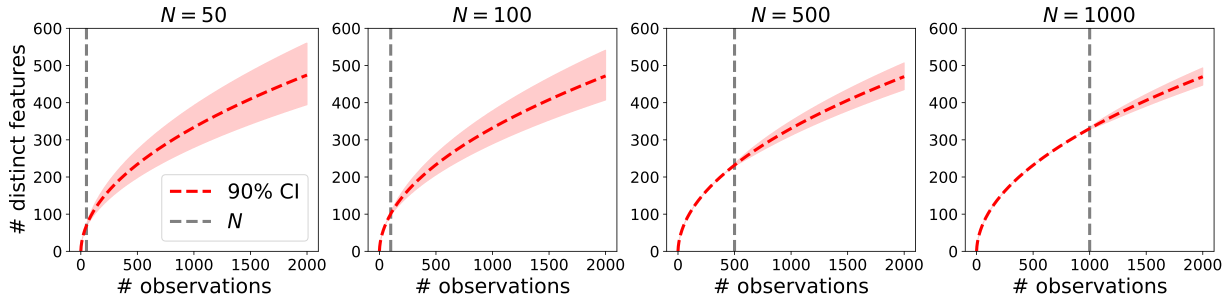

Figure S4: 90% centered credible interval for the expected number of distinct features $\mathbb{E}[U_N^{(M)} \mid Z_{1:N}]$ (y-axis) as a function of the sample size N (x-axis). We fix $\beta = 1$, $c = 5$, $\sigma = 0.5$, and total sequencing capacity $L = 2000$. In different subplots, we show $\mathbb{E}[U_N^{(M)} \mid Z_{1:N}]$ for different values of N. Here, the first N samples display exactly $K_N = \mathbb{E}[U_0^{(N)}]$ distinct features.

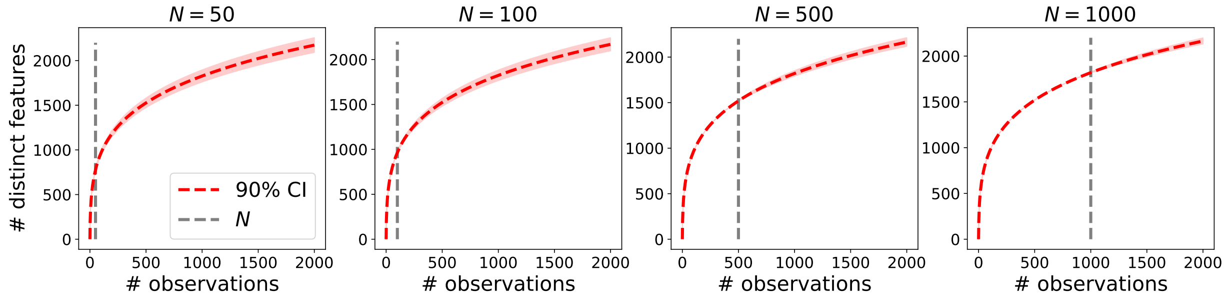

Figure S5: 90% centered credible interval for the expected number of distinct features $\mathbb{E}[U_N^{(M)} \mid Z_{1:N}]$ (y-axis) as a function of the sample size N (x-axis). We repeat the same exercise as in Figure S4 but now fix $\beta = 2$, $c = 1000$, $\sigma = 0.2$.

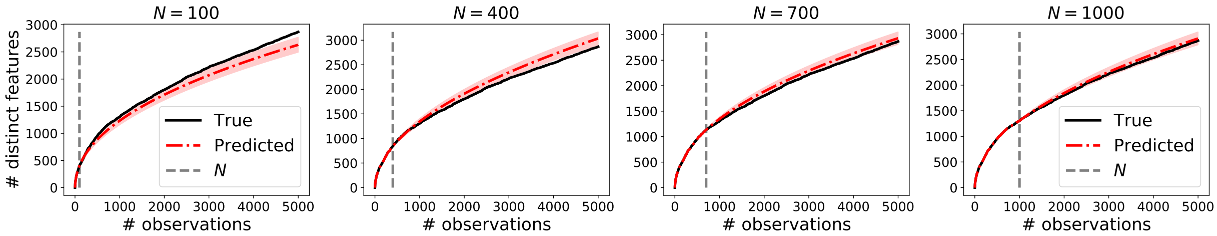

Figure S6: 99% credible interval centered around the posterior predictive mean (dashed red line) of the number of distinct features $U_N^{(M)} \mid Z_{1:N}$ (y-axis) as a function of the sample size N (x-axis). We fix $\beta = 1$, $c = 20$, $\sigma = 0.5$, and learn the parameters for different training size $N \in \{100, 400, 700, 1000\}$ across subplots for a total sequencing capacity $L = 5000$.

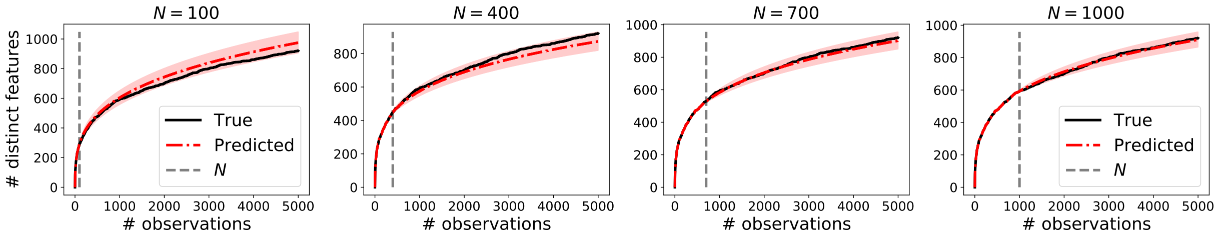

Figure S7: 99% credible interval centered around the posterior predictive mean (dashed red line) of the number of distinct features $U_N^{(M)} \mid Z_{1:N}$ (y-axis) as a function of the sample size N (x-axis). We repeat the same experiment as in Figure S6, but now for $\beta = 1$, $c = 100$, $\sigma = 0.25$.

to maximize a likelihood criterion, such as the marginal distribution of the feature counts $m_1, \ldots, m_K$, given in Equation (10). We found this method to work well both on real data, as displayed in Section 4, and on synthetic data. We here report some results in Figures S6 and S7. In general, and not surprisingly, the precision of our estimates increases with larger sample sizes.

In our synthetic experiments, as expected, the values maximizing the marginal likelihood converge to the underlying true values of the data generating process as the sample size $N \to \infty$. By performing a visual investigation, we find that indeed the negativd marginal likelihood is a convex function in each argument, with a unique, well-defined minimum (see Figures S8 to S10).

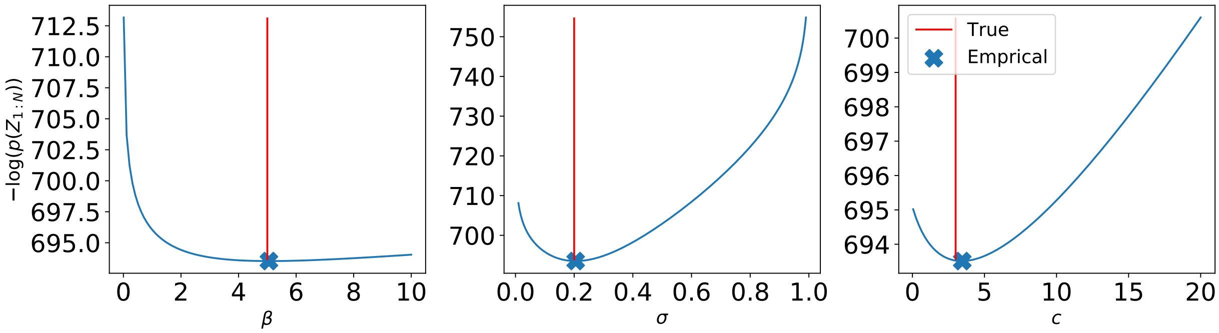

Figure S8: We draw a synthetic dataset of size $N = 10'000$ from a SSB with parameters $\beta = 5, \sigma = 0.1, c = 3$. In the left subplot, we plot the value of the negative marginal likelihood (vertical axis) as we vary the value of β (horizontal axis), keeping $\sigma = 0.1$ and $c = 3$ fixed at the true value. We repeat the same procedure, now varying σ and keeping $\beta = 5$, $c = 3$ fixed at their true value in the central subplot. Last, in the right subplot, we inspect the marginal likelihood as we vary the value of c, keeping $\beta = 5$, $\sigma = 0.1$. We then minimize numerically the negative log-likelihood, and report in each subplot with a blue cross the numerical value of the corresponding hyperparameter (horizontal axis) together with the corresponding marginal likelihood value (vertical axis).

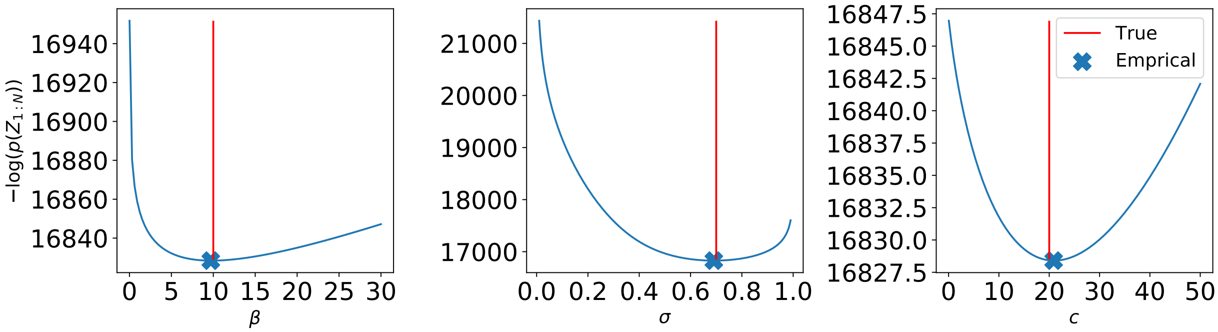

Figure S9: We repeat the same exercise of Figure S8 for $N = 1'000$, $\beta = 10$, $\sigma = 0.7$, $c = 20$.

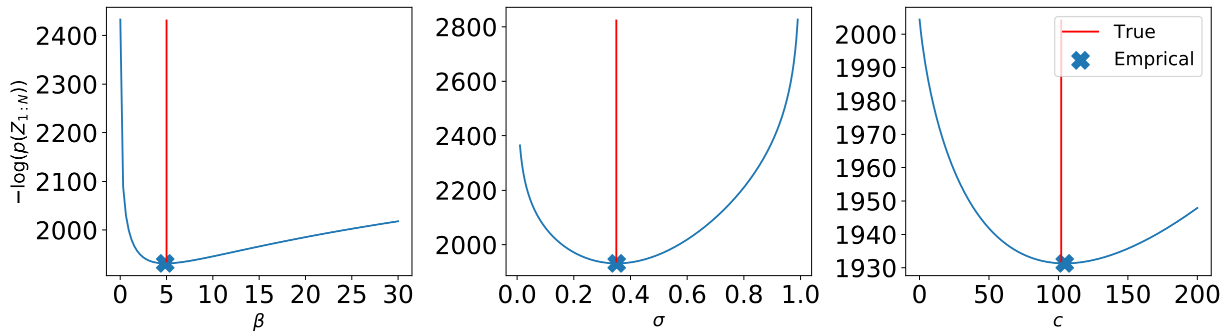

Figure S10: We repeat the same exercise as in Figures S8 and S9 for $N = 100$, $\beta = 5$, $\sigma = 0.35$ and $c = 100$.

When most of the features are very rare (e.g., they appear once or twice in the sample), we found that an alternative empirical Bayes approach, akin to the one adopted in Masoero et al. [2021], worked better, as further discussed in Section S7.

S7 Synthetic experiments from Zipf distributions

To compare the predictive performance of the SB-SP-Bernoulli process proposed in Section 3.1 to existing competing methods, we also consider synthetic data from Zipf-distributed frequencies (see Figure S11). That is to say, we imagine that there exists a countable number of features in the population, and that, for some $\xi > 0$, feature k is observed independently of any other feature with probability $\pi_k = (k+1)^{-\xi}$. An observation X_ℓ then a binary vector, in which, conditionally on the frequencies $\pi = (\pi_1, \pi_2, \ldots)$ the k-th coordinate is a Bernoulli random variable:

$$X_{\ell,k} \mid \pi \overset{i.i.d.}{\sim} \text{Bernoulli}(\pi_k), \quad \pi = \{(k+1)^{-\xi}\}_{k\geq 1}. \tag{S52}$$

We perform our simulations as follows: we fix a total sequencing capacity of $L = 2000$, and draw L i.i.d. samples from the model, following the recipe given in Equation (S52). For simulation purposes, we only consider the first $K = 10^5$ features to have non-zero probabil-

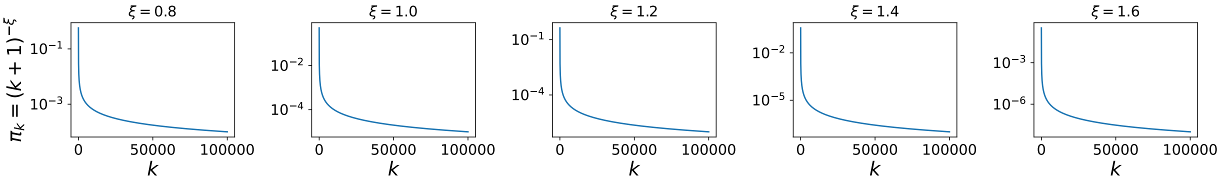

Figure S11: Frequencies distribution for different choices of the parameter ξ.

ity, i.e. $\pi_k = 0$, for all $k > K$. We compare the estimates of our proposed SB-SP-Bernoulli model (Section 3.1), to the stable beta-Bernoulli process [3BP], the linear program of Zou et al. [2016], the first four orders of the Jackknife estimator originally proposed in Burnham and Overton [1978] and recently employed in the genomics context by Gravel [2014], and the Good-Toulmin estimator, recently used in Chakraborty et al. [2019], with the two alternative smoothing choices described in Orlitsky et al. [2016]. Estimates for Bayesian methods are obtained by using the posterior predictive mean for the number of new variants conditionally on the observed sample, with hyper-parameters learned by numerically maximizing the marginal distribution (EFPF) of the features counts, as described in Section 4.11.

As expected, we find the nonparametric Bayesian estimators to do particularly well for larger values of the exponent ξ — that is when most features are exceedingly rare. The SB-SP-Bernoulli and the SB-SP-Bernoulli-parameter beta-Bernoulli processes performed comparably on these datasets, both in terms of estimation accuracy and uncertainty quantification, as displayed in Figure S12.

To better asses the predictive quality of the different methods, we ran extensive simulation experiments; for each value of $\xi \in \{0.8, 1, 1.2, 1.4, 1.6\}$, we generated $S = 100$ datasets of size $L = 2{,}000$, and for each value of $N \in \{10, 50, 100, 200\}$ we trained each method, and extrapolated to predict the number of new variants to be observed up to $M = L - N \in \{1990, 1950, 1900, 1800\}$ remaining samples. We report as measure of accu-

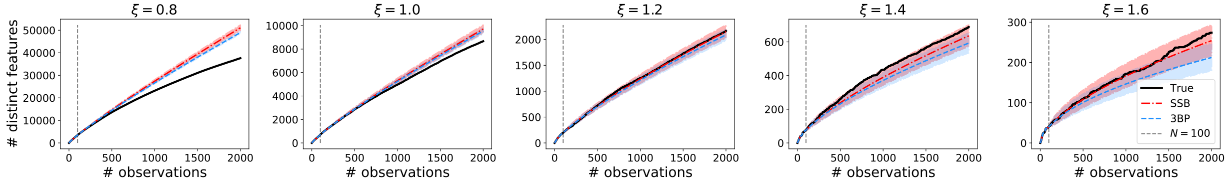

Figure S12: Estimates for the number of new features for the SB-SP-Bernoulli (red) and the stable beta-Bernoulli (blue) processes as the exponent ξ varies across subplots. Shaded regions cover a 95% credible interval around the predictive mean. The solid black line represents the true counts. Here, the training is done using the first $N = 100$ observations, and extrapolating up to the remaining $M = 1900$ observations.

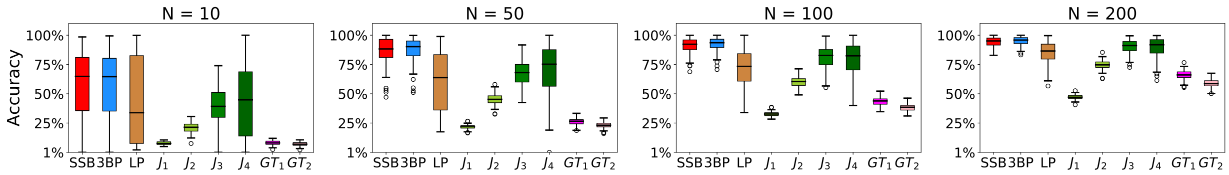

Figure S13: Accuracy of the competing methods (SB-SP-Bernoulli [SSP], stable beta-Bernoulli [3BP], Jackknife [J], linear program [LP], Good-Toulmin [GT]) on simulated data from a Zipf model (Equation (S52)) with parameter $\xi = 1.2$. For $L = 2000$, we report $v_{N,a}^{(M)}$ as N increases, for $M = L - N$. For each N, results across $S = 100$ datasets are reported in the boxplots.

racy the percentage accuracy incurred by each estimation method $v_{N,a}^{(M)}$, defined in Equation (19), at the largest extrapolation level $M = L - N$, across different values of N and all $S = 100$ simulation studies. Results are reported via boxplots in Figures S13 to S15. While all methods improve their performance with larger sample sizes, we find that the BNP estimators (SSP, 3BP) provide relatively more accurate results for smaller samples sizes (e.g., $N = 10, N = 50$ in Figures S13 and S14). The performance of the BNP methods exceed those of competing methods for larger values of the exponent ($\xi \in \{1.2, 1.4, 1.6\}$), while higher order Jackknife and linear programs tend to do better for smaller values of the exponent ($\xi \in \{0.8, 1\}$).

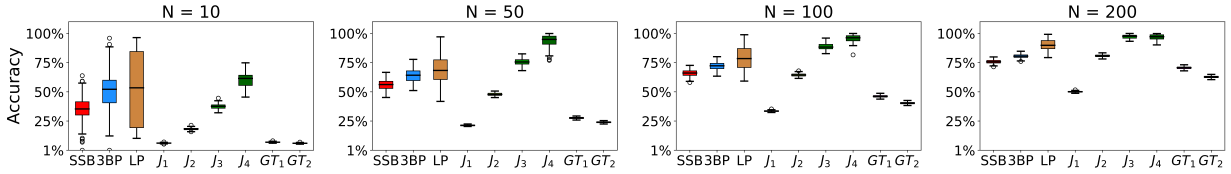

Figure S14: Accuracy of the competing methods (SB-SP-Bernoulli [SSP], stable beta-Bernoulli [3BP], Jackknife [J], linear program [LP], Good-Toulmin [GT]) on simulated data from a Zipf model (Equation (S52)) with parameter $\xi = 0.8$. For $L = 2000$, we report $v_{N,a}^{(M)}$ as N increases, for $M = L - N$. For each N, results across $S = 100$ datasets are reported in the boxplots.

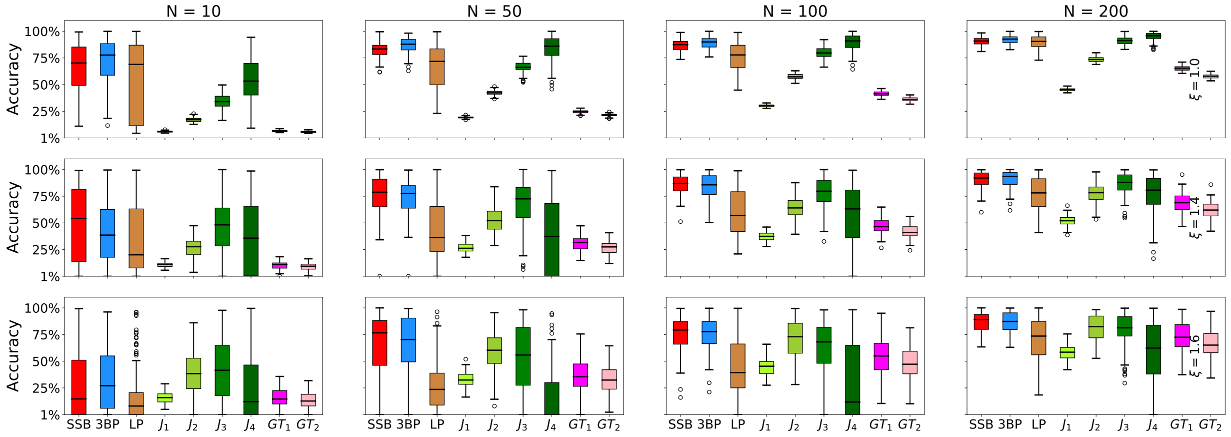

Figure S15: Accuracy of the competing methods (SB-SP-Bernoulli [SSP], stable beta-Bernoulli [3BP], Jackknife [J], linear program [LP], Good-Toulmin [GT]) on simulated data from a Zipf model (Equation (S52)) with parameter $\xi \in \{1, 1.4, 1.6\}$ (top row, center row, bottom row). For $L = 2000$, we report $v_{N,a}^{(M)}$ as N increases, for $M = L - N$. For each N, results across $S = 100$ datasets are reported in the boxplots.

S8 Additional experiments on the gnomAD dataset

S8.1 Experimental setup

In order to run our experiments, we use data from the gnomAD (genome aggregation dataset) discovery project [Karczewski et al., 2020], the largest and most comprehensive publicly available human genome dataset. We follow the same experimental setup adopted in Masoero et al. [2021]. We briefly summarize this setup in this section. The gnomAD dataset contains 125'748 exomes sequences (i.e. protein-coding regions of the genome), from 8 main populations. Sample size varies widely across sub populations, e.g. the "Other" subgroup counts about 3'000 observations, while "South East Asian" contains almost 16'000 individuals (see Karczewski et al. [2020] for additional details).

For privacy reasons, not all individual sequences are accessible. Hence, in order to run our analysis we generate synthetic data which closely resembles the true data as follows. For every subpopulation with N individuals and every position $j = 1, \dots, K$ in the exome, we have access to the total number of individuals N_j showing variation at position j. We compute the empirical frequency of variation at site j, $\hat{\theta}_j := N_j/N$ for all $j = 1, \dots, K$. Our data is then generated by sampling independent Bernoulli random vectors $X_1, \dots, X_N$, with $X_n = [x_{n,1}, \dots, x_{n,K}]$. The entries in the vector are independent Bernoulli random variables, $x_{n,j} \sim \text{Bernoulli}(\hat{\theta}_j)$.

S8.2 Results from the gnomAD data

For each of eight subpopulations in the data, we performed the following experiment. Let $\hat{\theta} = [\hat{\theta}_1, \dots, \hat{\theta}_{K_{\max}}] \subseteq [0, 1]$ denote the "genetic signature" of the population, with $\hat{\theta}_k = N_k/N$, with N_{tot} the total number of individuals in the population and N_k the number

of individuals in the population displaying such variant, $1 \leq N_k \leq N_{tot}$. Then, for each population, we generate $S = 50$ datasets by drawing N_{tot} i.i.d. binary random vectors of length $K_{\max}$ as described above, with biases given by $\hat{\theta}$. We then retain for each dataset $N \in \{50, 100\}$ observations for training, and try to predict the number of new variants that are going to be observed if we were to sample additional $M = N_{tot} - N$ observations.

In a nutshell, also on this data, the findings are similar to the results obtained on the MSK-IMPACT cancer data. In particular, we find that when the sample size N is small, the proposed SB-SP Bernoulli model leads to predictions that are often comparable or more accurate than competing methods.

First, we report the accuracy metric $v_N^{(M)}$ for eight subpopulations in gnomAD, Afroamerican (Amr.), South East Asian (SE. As.), Other East Asian (Ot. E. As.), Finnish (Fin.), South European (S. Eu.), Swedish (Swe.), South Asian (S. As.) and the remaining Other. In Figure S16 we show results (over $S = 50$ Monte-Carlo re-draws of the data from the estimated frequencies $\hat{\theta}$) of retaining $N = 50$ datapoints for training, and extrapolating to the largest available sample size M. In Figure S17 we report results for the same metric, with training performed by retaining $N = 100$ datapoints.

Next, we provider boxplots that report the (aggregated) accuracy of the metric $v_N^{(M)}$ across all the eight populations, and all the $S = 50$ Monte-Carlo draws (so that each boxplot reports the accuracy of a total of $50 \times 8 = 400$ accuracy values), for $N = 50$ (Figure S18) as well as $N = 100$ (Figure S19).

S8.3 Additional boxplots

Since in Figure S18 and Figure S19 we are aggregating result in which N is consistent for all populations, but M differs, we also report boxplots of each subpopulation individually.

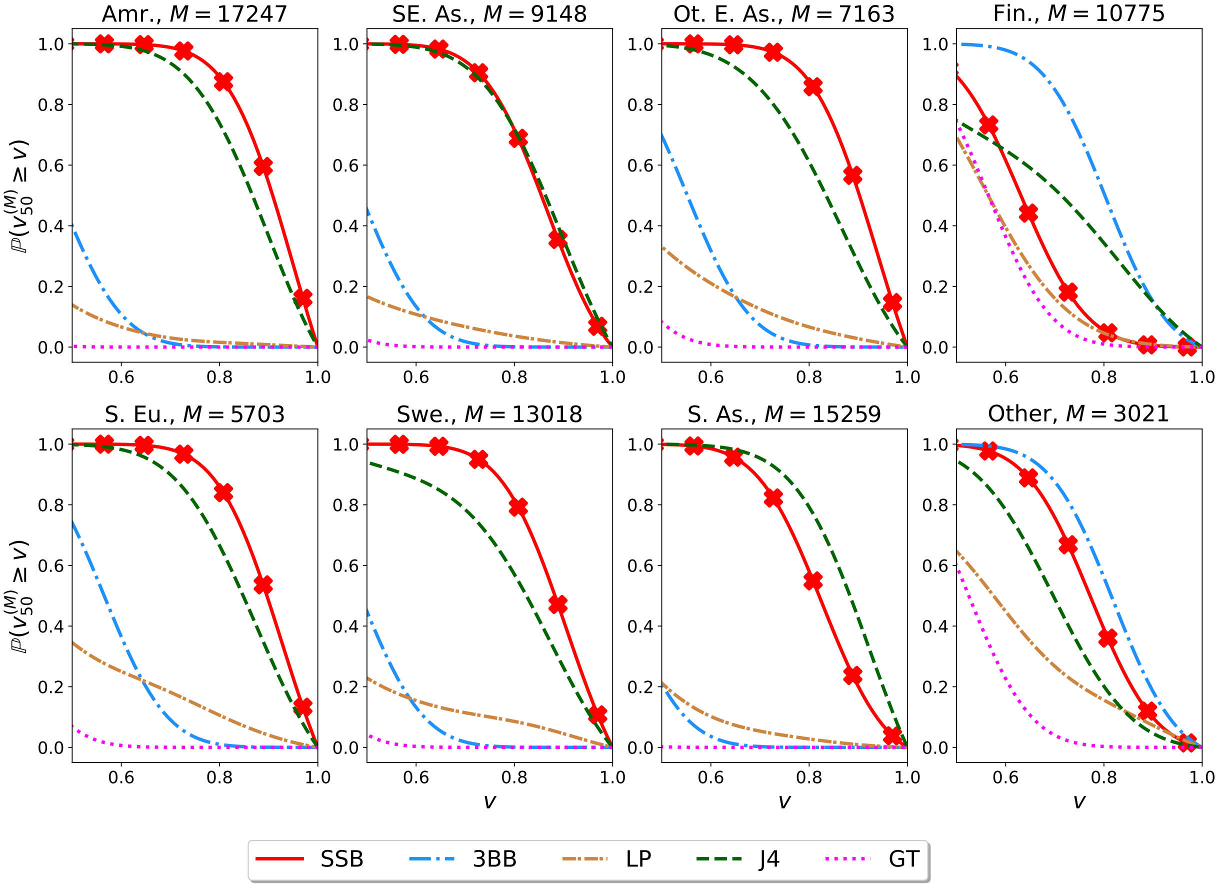

Figure S16: Accuracy metric $v_{50}^{(M)}$ for eight subpopulations in the gnomAD dataset. For each subpopulation we retain $N = 50$ observations for training, and extrapolate to the largest possible value M. Results are over $S = 50$ Monte-Carlo draws of the data, as described in Section S8.1

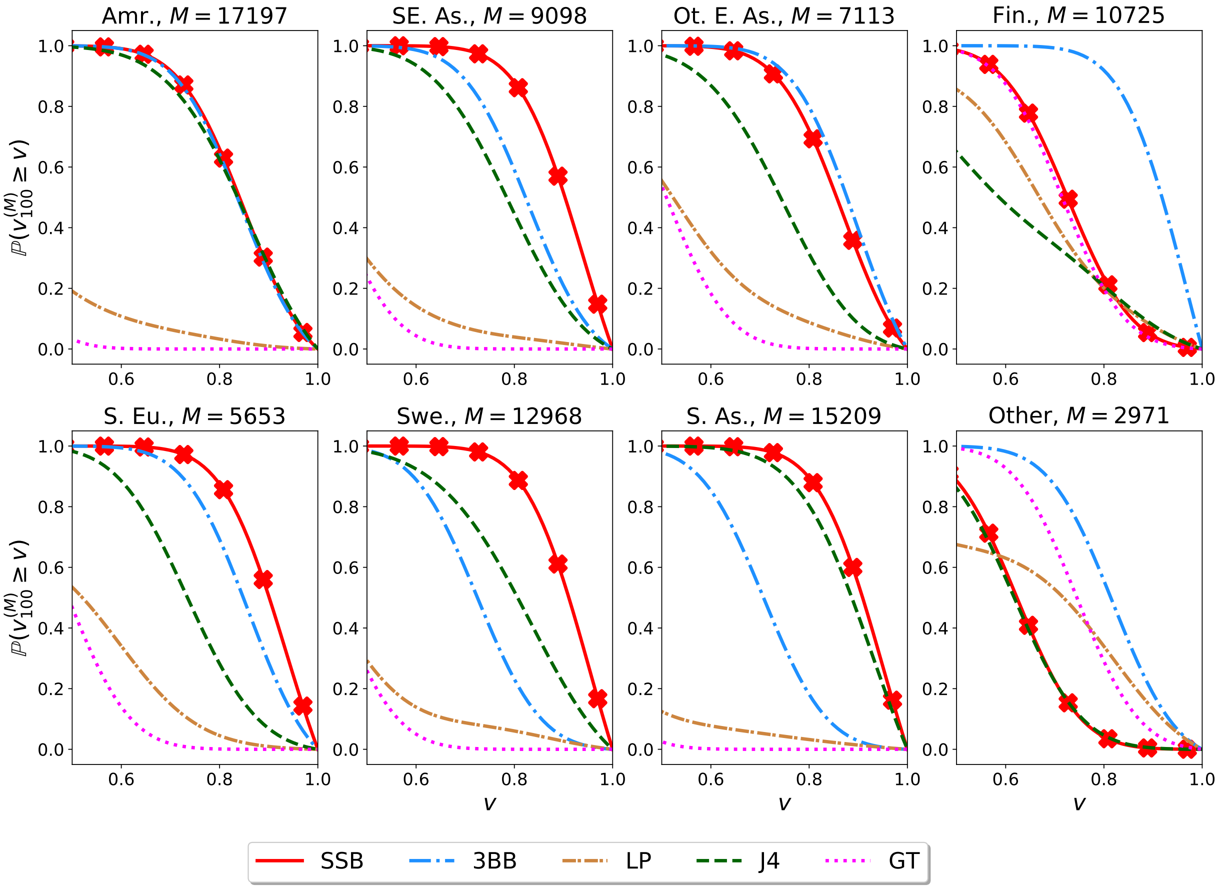

Figure S17: Same setup as in Figure S16, now for $N = 100$.

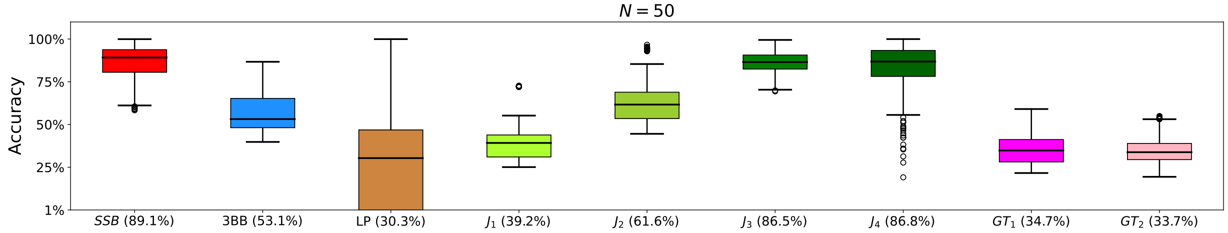

Figure S18: Accuracy of the compared methods, now over all the eight subpopulations and over 50 Monte Carlo draws for each population. $N = 50$, and M is set to be the largest possible extrapolation size for each subpopulation.

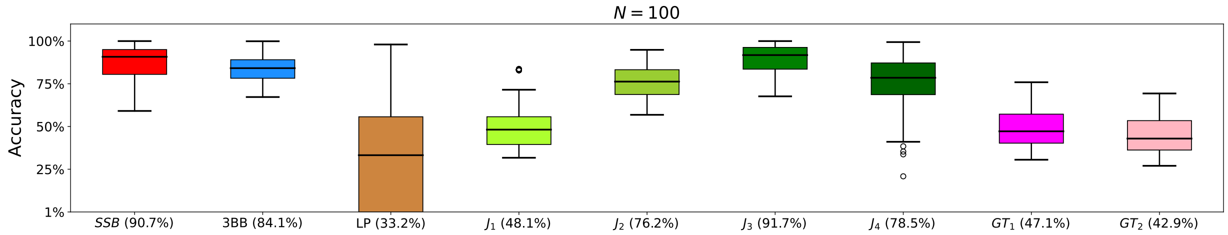

Figure S19: Same setup as in Figure S18, now for $N = 100$.

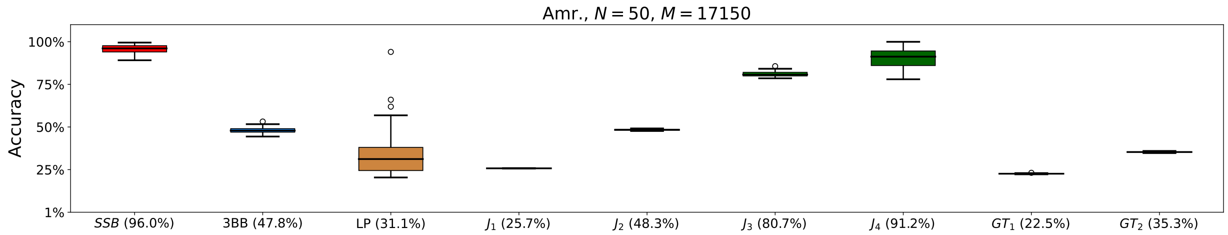

Figure S20: Same setup as in Figure S18, but only for the American subpopulation.

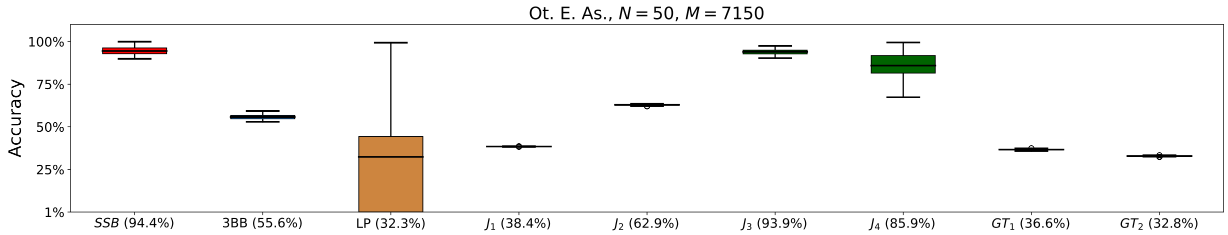

Figure S21: Same setup as in Figure S18, but only for the Other East Asian subpopulation.

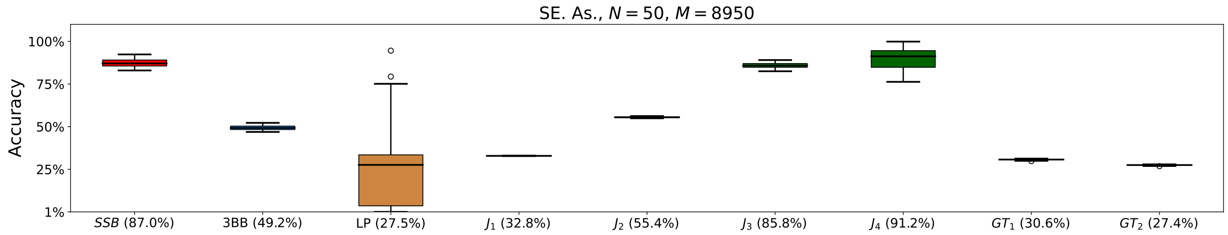

Figure S22: Same setup as in Figure S18, but only for the East Asian subpopulation.

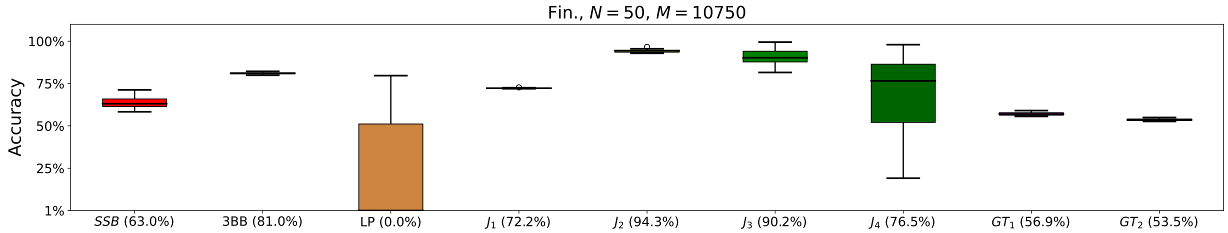

Figure S23: Same setup as in Figure S18, but only for the Finnish subpopulation.

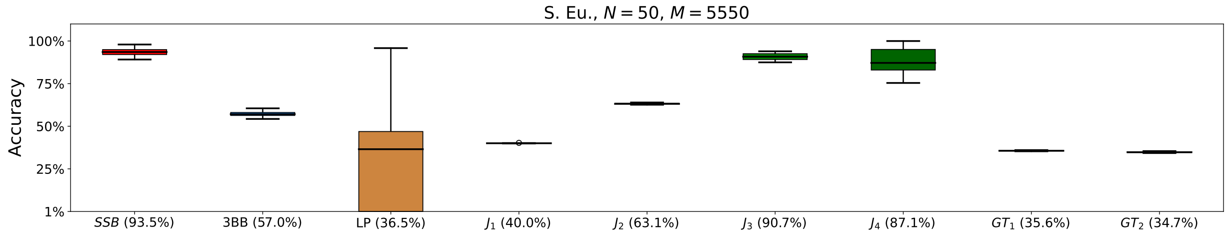

Figure S24: Same setup as in Figure S18, but only for the Southern European subpopulation.

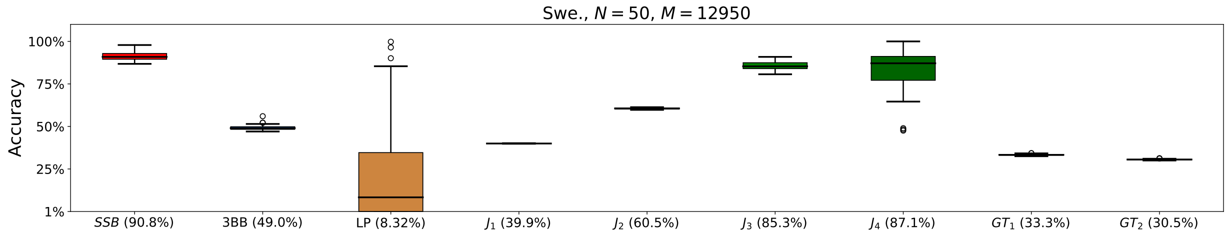

Figure S25: Same setup as in Figure S18, but only for the Swedish subpopulation.

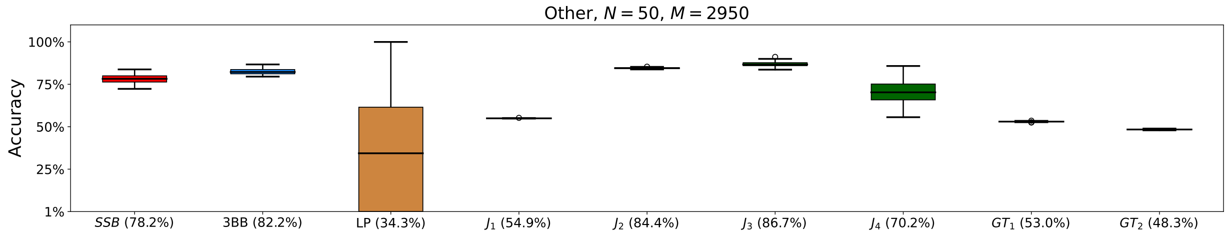

Figure S26: Same setup as in Figure S18, but only for the “Other” subpopulation.

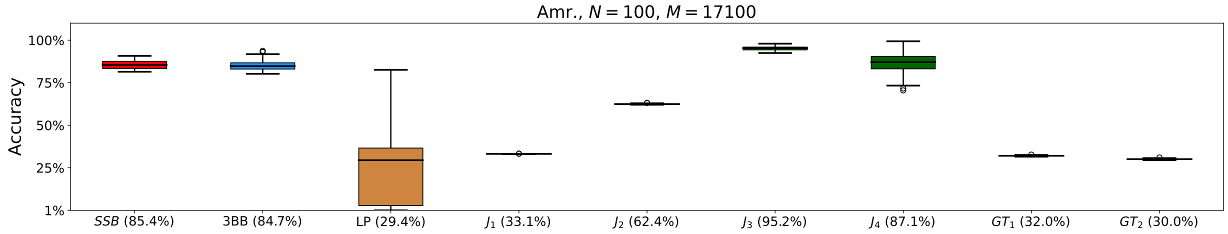

Figure S27: Same setup as in Figure S19, but only for the American subpopulation.

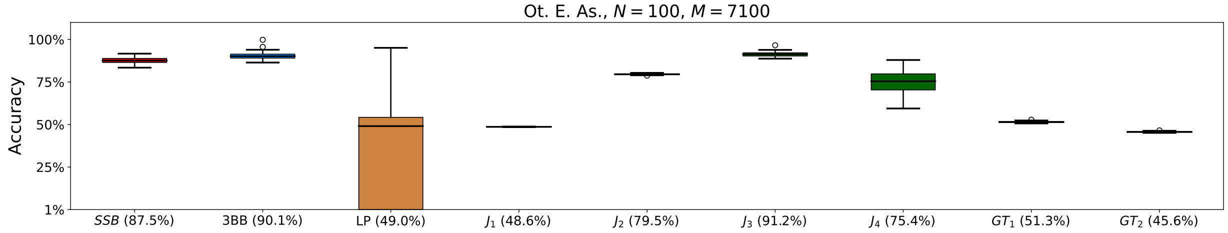

Figure S28: Same setup as in Figure S19, but only for the Other East Asian subpopulation.

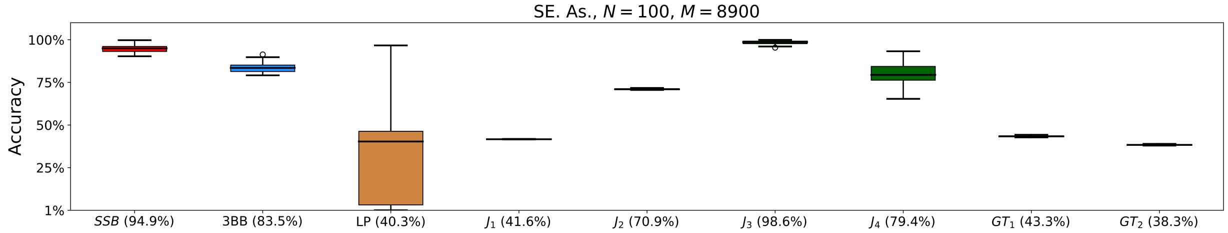

Figure S29: Same setup as in Figure S19, but only for the East Asian subpopulation.

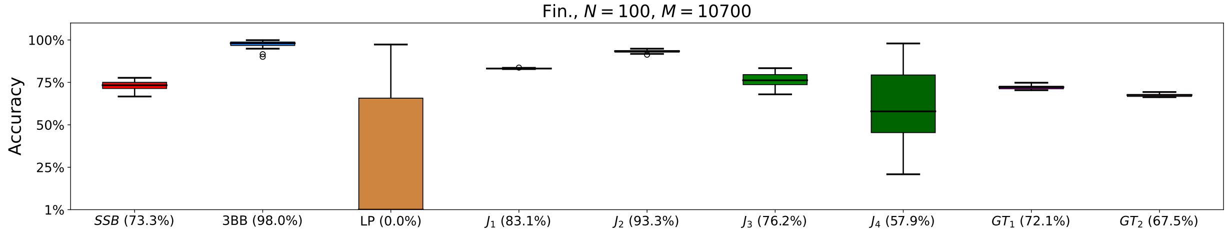

Figure S30: Same setup as in Figure S19, but only for the Finnish subpopulation.

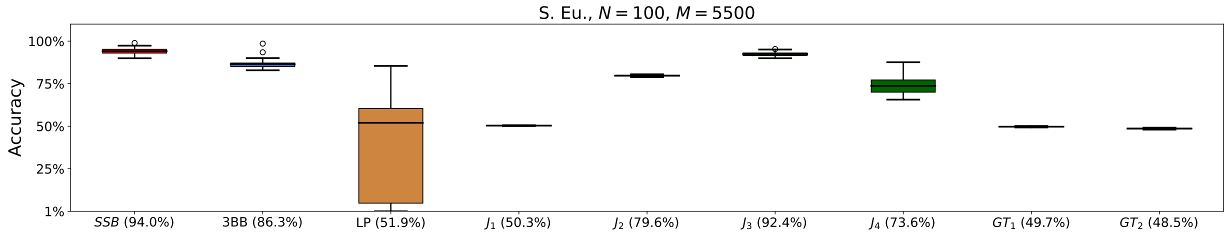

Figure S31: Same setup as in Figure S19, but only for the Southern European subpopulation.

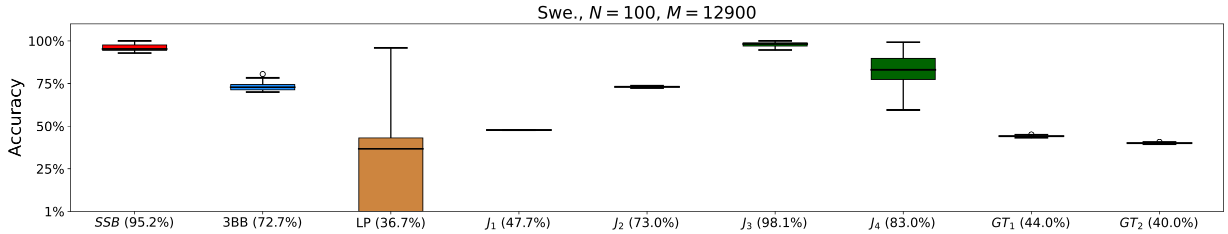

Figure S32: Same setup as in Figure S19, but only for the Swedish subpopulation.

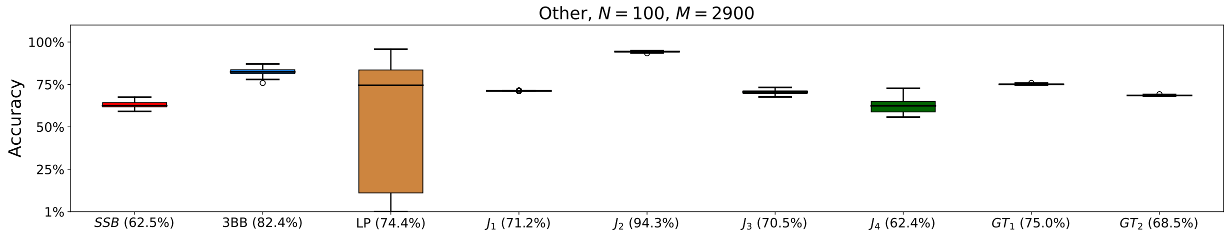

Figure S33: Same setup as in Figure S19, but only for the "Other" subpopulation.

References

T. Broderick, M. I. Jordan, and J. Pitman. Beta processes, stick-breaking and power laws. *Bayesian analysis*, 7:439–476, 2012.

T. Broderick, J. Pitman, and M. I. Jordan. Feature allocations, probability functions, and paintboxes. *Bayesian Analysis*, 8:801–836, 2013.

K. P. Burnham and W. S. Overton. Estimation of the size of a closed population when capture probabilities vary among animals. *Biometrika*, 65:625–633, 1978.

S. Chakraborty, A. Arora, C. B. Begg, and R. Shen. Using somatic variant richness to mine signals from rare variants in the cancer genome. *Nature Communications*, 10:5506, 2019.

D. J. Daley and D. Vere-Jones. *An introduction to the theory of point processes. Vol. II.* Probability and its Applications (New York). Springer, New York, second edition, 2008. General theory and structure.

T. S. Ferguson and M. J. Klass. A representation of independent increment processes without Gaussian components. *Ann. Math. Statist.*, 43:1634–1643, 1972.

I. S. Gradshteyn and I. M. Ryzhik. *Table of integrals, series, and products.* Elsevier/Academic Press, Amsterdam, 2007.

S. Gravel. Predicting discovery rates of genomic features. *Genetics*, 197:601–610, 2014.

L. F. James. Bayesian Poisson calculus for latent feature modeling via generalized Indian buffet process priors. *Ann. Statist.*, 45:2016–2045, 2017.

L. F. James, P. Orbanz, and Y. W. Teh. Scaled subordinators and generalizations of the Indian buffet process. *arXiv preprint arXiv:1510.07309*, 2015.

O. Kallenberg. Commutativity properties of conditional distributions and Palm measures. *Commun. Stoch. Anal.*, 4:21–34, 2010.

O. Kallenberg. *Random measures, theory and applications*. Springer, Cham, 2017.

K. J. Karczewski, L. C. Francioli, G. Tiao, B. B. Cummings, J. Alföldi, Q. Wang, R. L. Collins, K. M. Laricchia, A. Ganna, and D. P. Birnbaum. The mutational constraint spectrum quantified from variation in 141,456 humans. *Nature*, 581(7809):434–443, 2020.

Y. Kim, L. James, and R. Weissbach. Bayesian analysis of multistate event history data: beta-dirichlet process prior. *Biometrika*, 99:127–140, 2012.

J. Kingman. Completely random measures. *Pacific Journal of Mathematics*, 21:59–78, 1967.

J. Kingman. *Poisson Processes*. Oxford Studies in Probability. Clarendon Press, 1992.

J. Lee, P. Müller, S. Sengupta, K. Gulukota, and Y. Ji. Bayesian inference for intratumour heterogeneity in mutations and copy number variation. *J. R. Stat. Soc. Ser. C. Appl. Stat.*, 65:547–563, 2016.

A. Lijoi and I. Prünster. Models beyond the Dirichlet process. In N. L. Hjort, C. Holmes, P. Müller, and S. Walker, editors, *Bayesian Nonparametrics*, pages 80–136. Cambridge University Press, 2010.

L. Masoero, F. Camerlenghi, S. Favaro, and T. Broderick. More for less: predicting and maximizing genomic variant discovery via Bayesian nonparametrics. *Biometrika*, 2021. doi: 10.1093/biomet/asab012.

A. Orlitsky, A. T. Suresh, and Y. Wu. Optimal prediction of the number of unseen species. *Proceedings of the National Academy of Sciences*, 113:13283–13288, 2016.

Y. Teh and D. Gorur. Indian buffet processes with power-law behavior. *Advances in neural information processing systems*, 22:1838–1846, 2009.

J. Zou, G. Valiant, P. Valiant, K. Karczewski, S. O. Chan, K. Samocha, M. Lek, S. Sunyaev, M. Daly, and D. G. MacArthur. Quantifying unobserved protein-coding variants in human populations provides a roadmap for large-scale sequencing projects. *Nature Communications*, 7:13293, 2016.
