# Supplementary figures and images for: Scaled Process Priors for Bayesian Nonparametric Estimation of the Unseen Genetic Variation
Source: J Am Stat Assoc. 2022 Sep 29;119(545):320–31. doi: 10.1080/01621459.2022.2115918 (PMC11073059; doi:10.1080/01621459.2022.2115918)

Ot. E. As.,  $N = 100$ ,  $M = 7100$

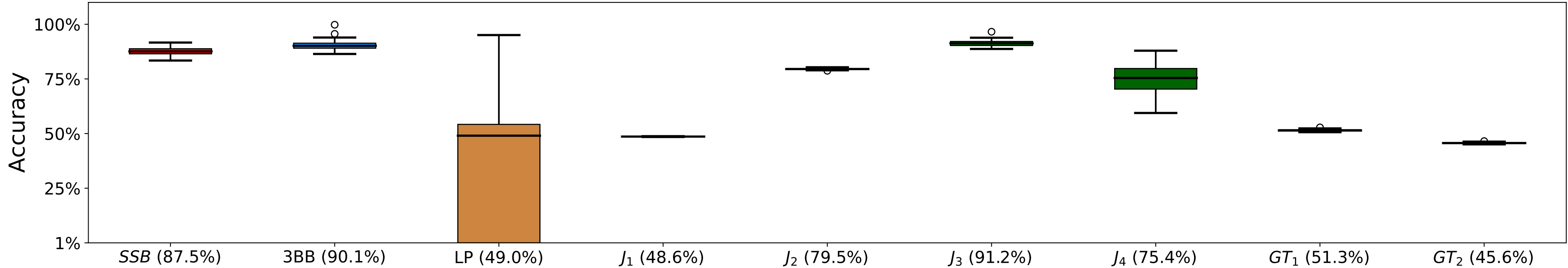

Supplement: Supplemental Material [file UASA_A_2115918_SM5205.zip › supplementary_files/ScaledProcesses/gnomAD/Plots/N_100_eas_oea.pdf]

Fin.,  $N = 50$ ,  $M = 10750$

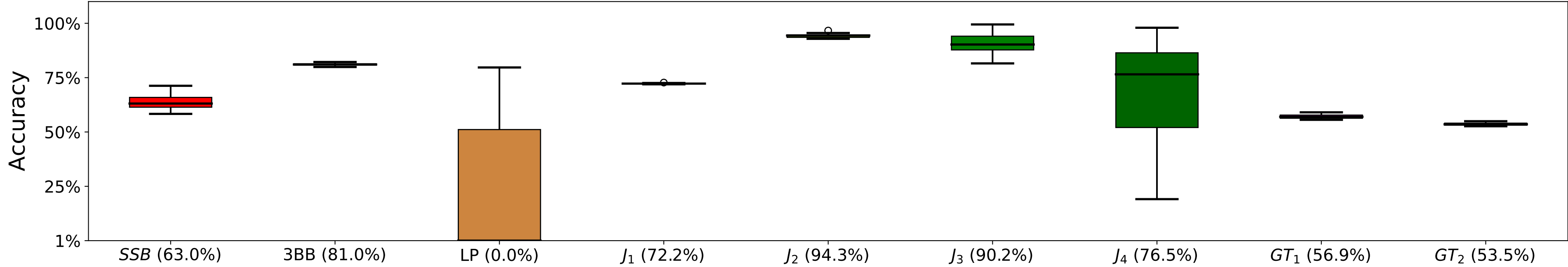

Supplement: Supplemental Material [file UASA_A_2115918_SM5205.zip › supplementary_files/ScaledProcesses/gnomAD/Plots/N_50_fin.pdf]

SE. As.,  $N = 50$ ,  $M = 8950$

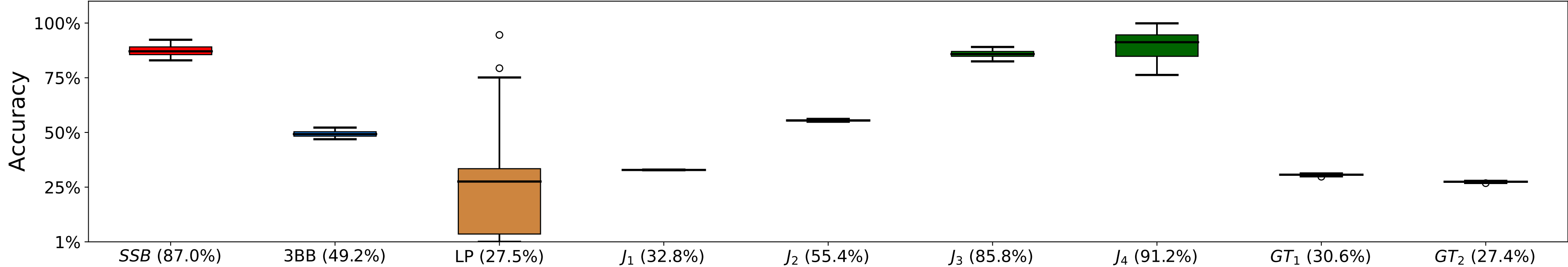

Supplement: Supplemental Material [file UASA_A_2115918_SM5205.zip › supplementary_files/ScaledProcesses/gnomAD/Plots/N_50_eas.pdf]

Swe.,  $N = 100$ ,  $M = 12900$

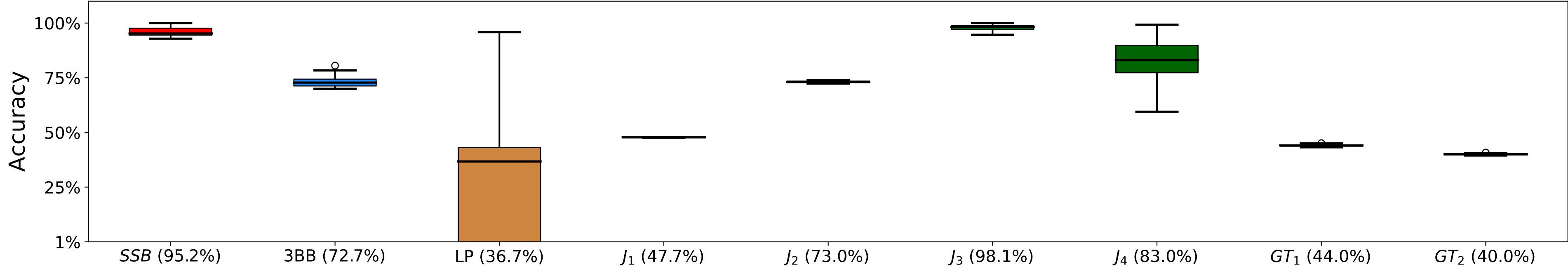

Supplement: Supplemental Material [file UASA_A_2115918_SM5205.zip › supplementary_files/ScaledProcesses/gnomAD/Plots/N_100_nfe_swe.pdf]

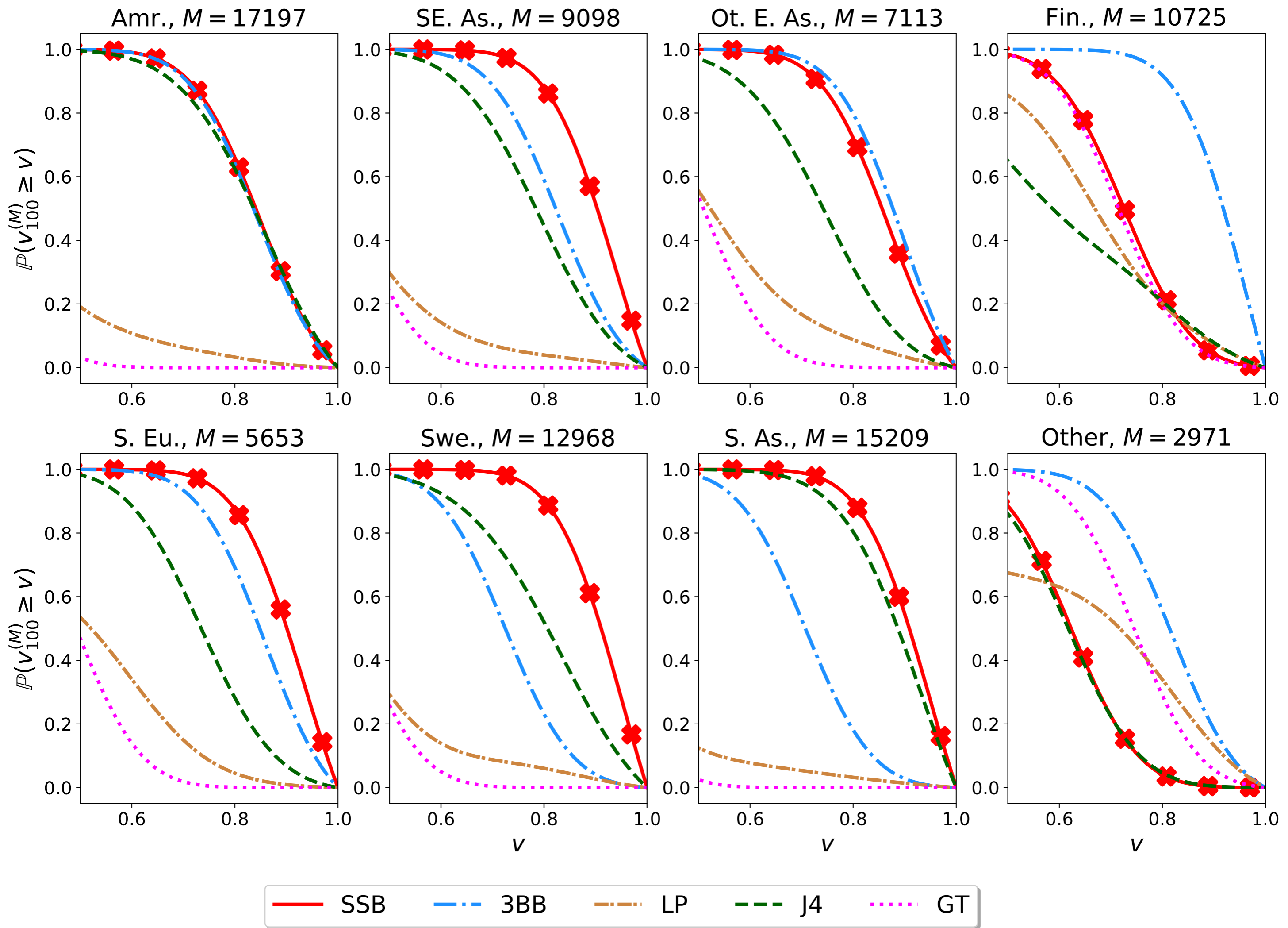

Supplement: Supplemental Material [file UASA_A_2115918_SM5205.zip › supplementary_files/ScaledProcesses/gnomAD/Plots/gnomAD_small_N_100_M_2971_all.pdf]

Amr.,  $N = 50$ ,  $M = 17150$

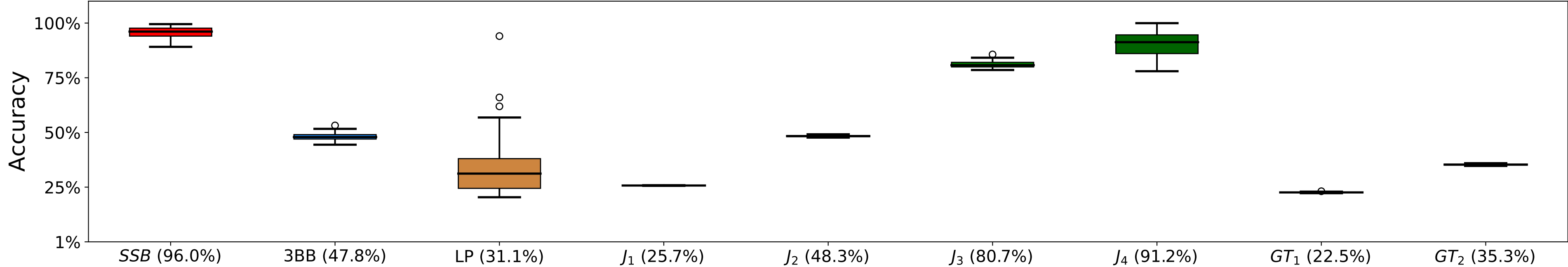

Supplement: Supplemental Material [file UASA_A_2115918_SM5205.zip › supplementary_files/ScaledProcesses/gnomAD/Plots/N_50_amr.pdf]

Other,  $N = 50$ ,  $M = 2950$ 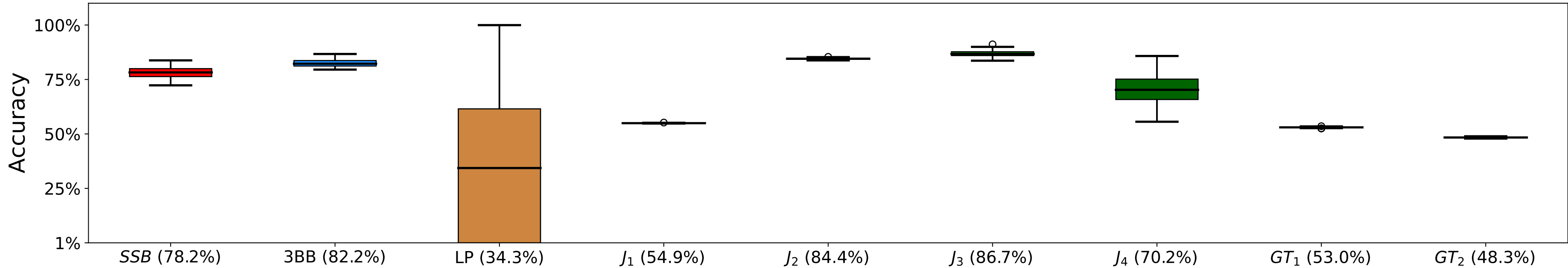

Supplement: Supplemental Material [file UASA_A_2115918_SM5205.zip › supplementary_files/ScaledProcesses/gnomAD/Plots/N_50_oth.pdf]

Amr.,  $N = 100$ ,  $M = 17100$

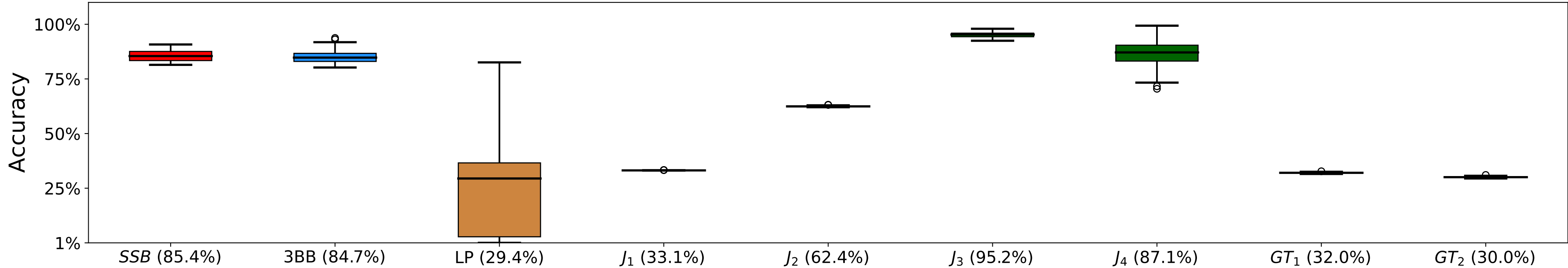

Supplement: Supplemental Material [file UASA_A_2115918_SM5205.zip › supplementary_files/ScaledProcesses/gnomAD/Plots/N_100_amr.pdf]

Other,  $N = 100$ ,  $M = 2900$ 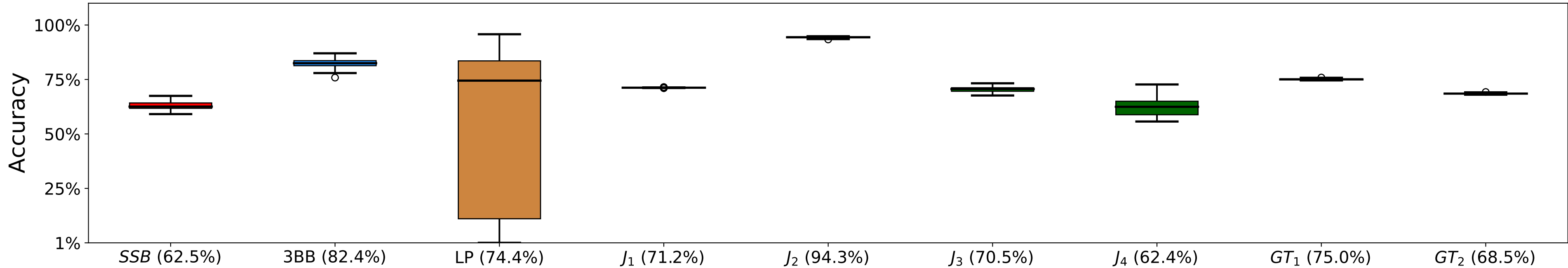

Supplement: Supplemental Material [file UASA_A_2115918_SM5205.zip › supplementary_files/ScaledProcesses/gnomAD/Plots/N_100_oth.pdf]

SE. As.,  $N = 100$ ,  $M = 8900$

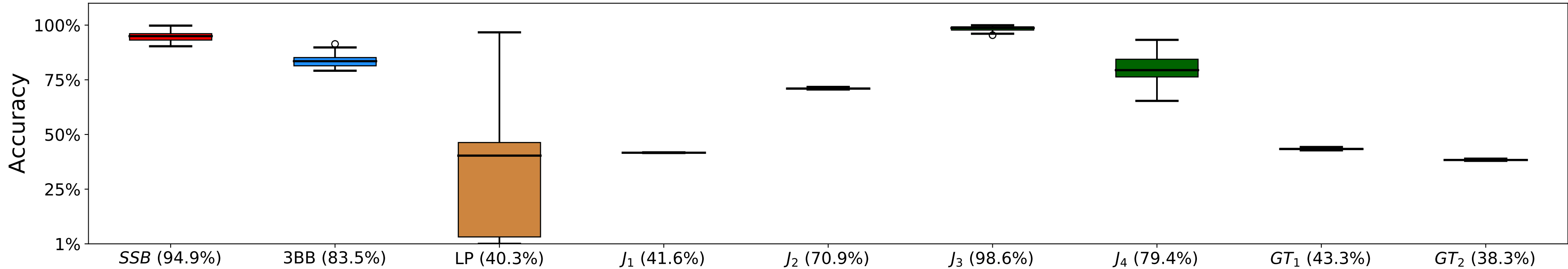

Supplement: Supplemental Material [file UASA_A_2115918_SM5205.zip › supplementary_files/ScaledProcesses/gnomAD/Plots/N_100_eas.pdf]

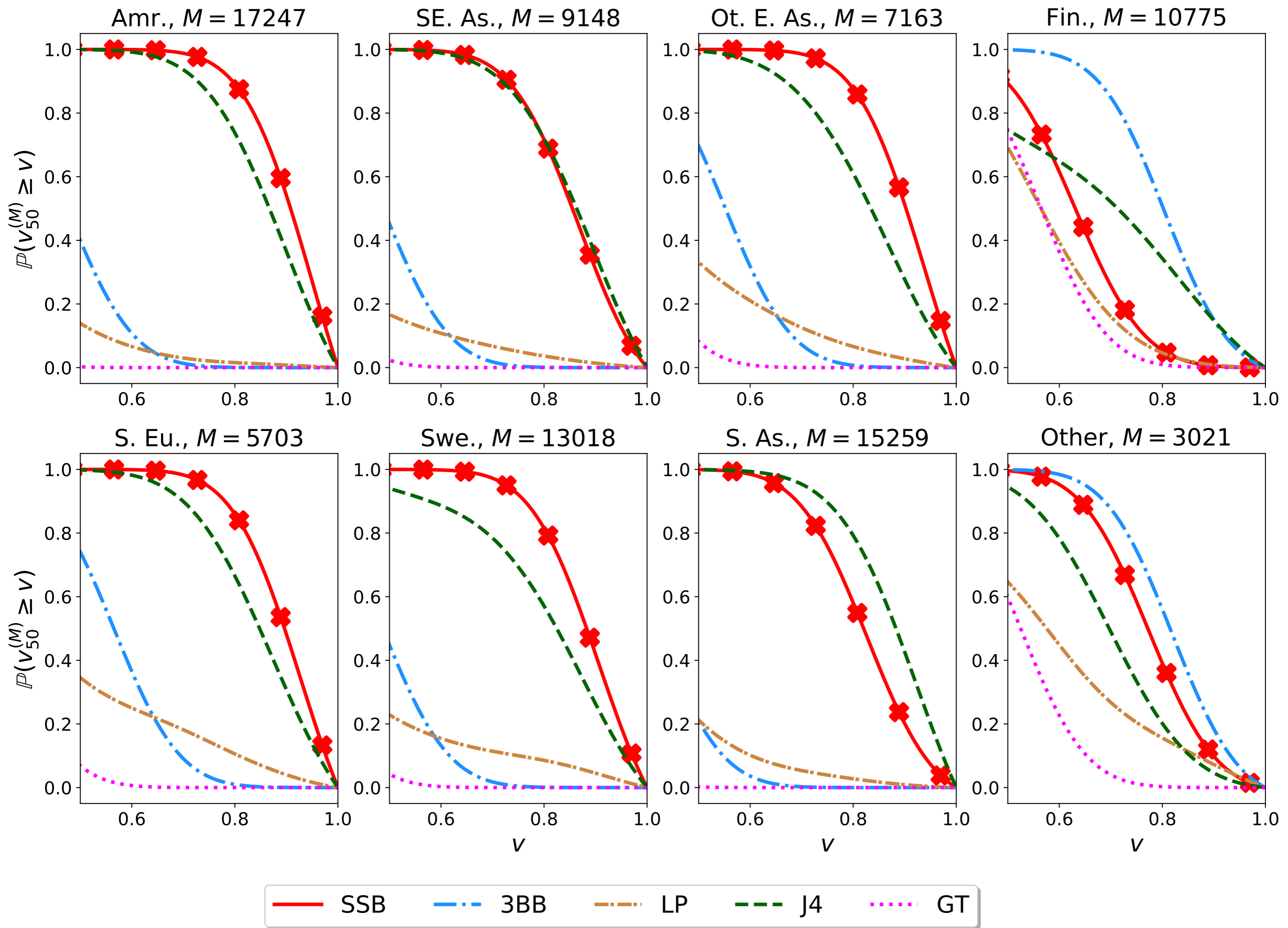

Supplement: Supplemental Material [file UASA_A_2115918_SM5205.zip › supplementary_files/ScaledProcesses/gnomAD/Plots/gnomAD_small_N_50_M_3021_all.pdf]

S. Eu.,  $N = 100$ ,  $M = 5500$

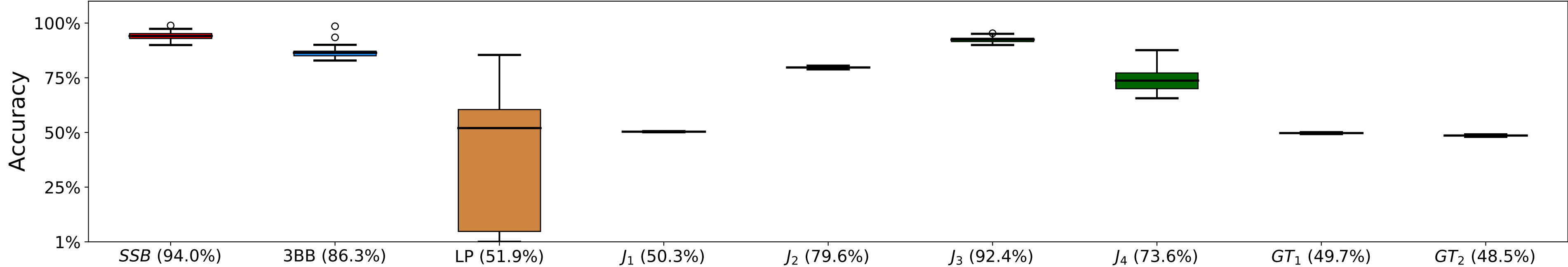

Supplement: Supplemental Material [file UASA_A_2115918_SM5205.zip › supplementary_files/ScaledProcesses/gnomAD/Plots/N_100_nfe_seu.pdf]

Fin.,  $N = 100$ ,  $M = 10700$

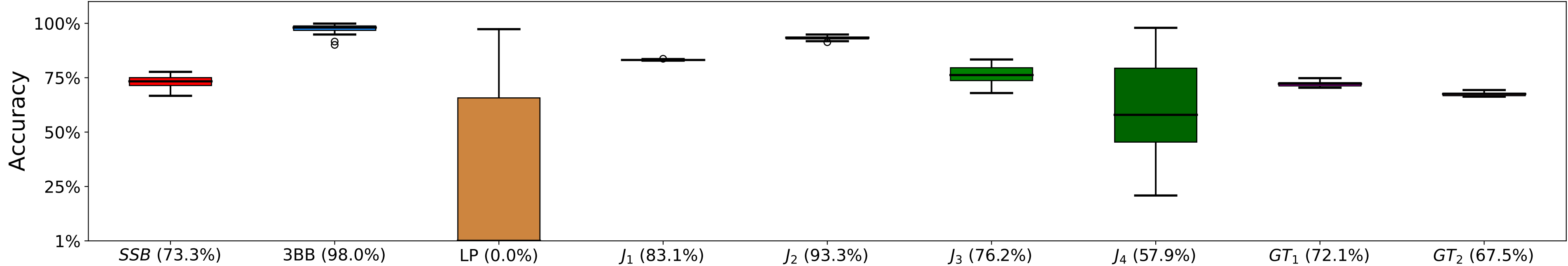

Supplement: Supplemental Material [file UASA_A_2115918_SM5205.zip › supplementary_files/ScaledProcesses/gnomAD/Plots/N_100_fin.pdf]

S. Eu.,  $N = 50$ ,  $M = 5550$

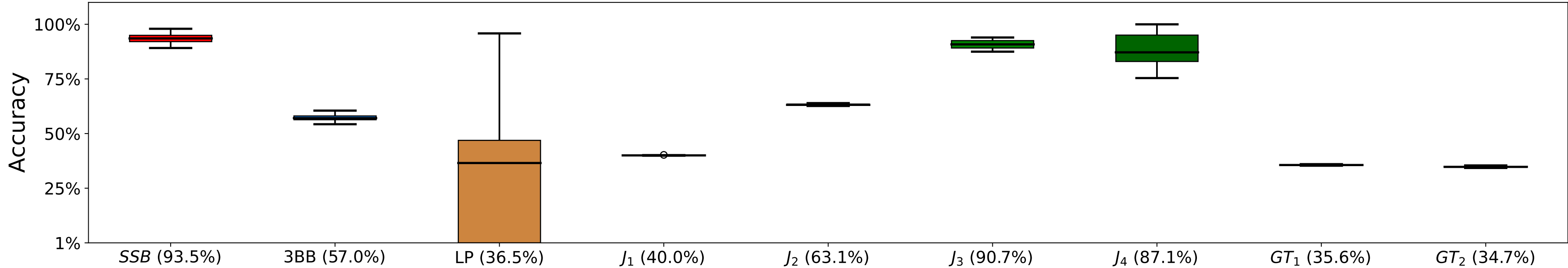

Supplement: Supplemental Material [file UASA_A_2115918_SM5205.zip › supplementary_files/ScaledProcesses/gnomAD/Plots/N_50_nfe_seu.pdf]

S. As.,  $N = 50$ ,  $M = 15150$

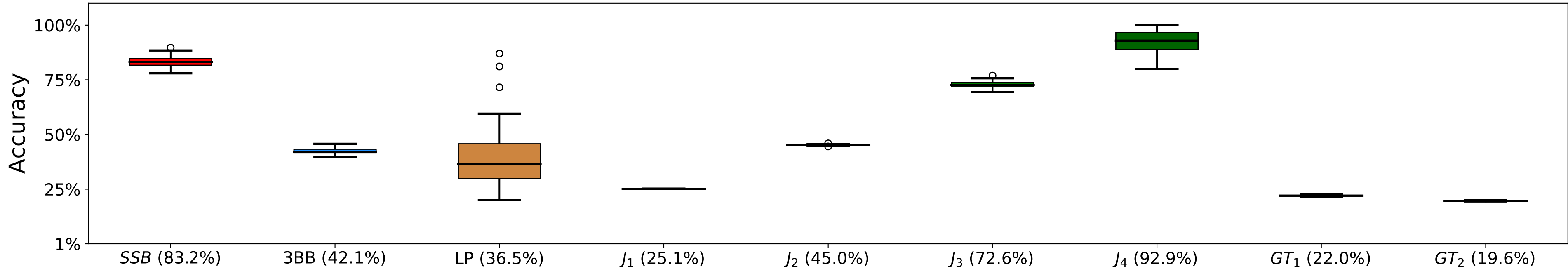

Supplement: Supplemental Material [file UASA_A_2115918_SM5205.zip › supplementary_files/ScaledProcesses/gnomAD/Plots/N_50_sas.pdf]

S. As.,  $N = 100$ ,  $M = 15100$

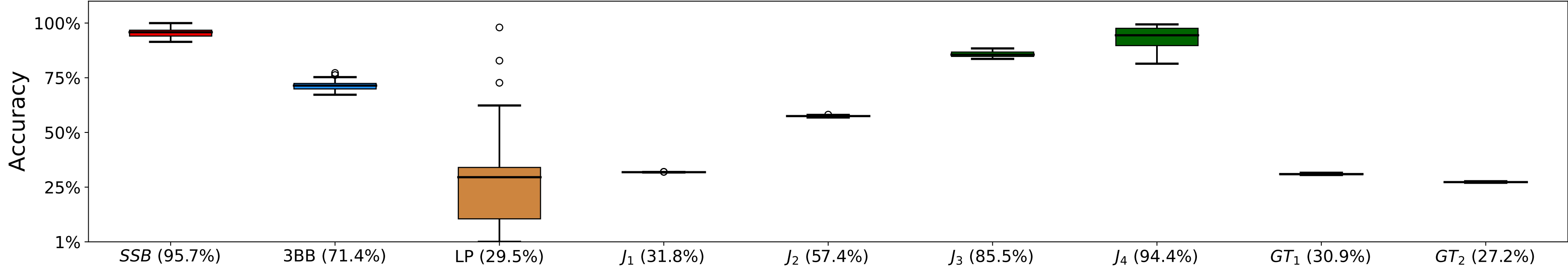

Supplement: Supplemental Material [file UASA_A_2115918_SM5205.zip › supplementary_files/ScaledProcesses/gnomAD/Plots/N_100_sas.pdf]

Swe.,  $N = 50$ ,  $M = 12950$

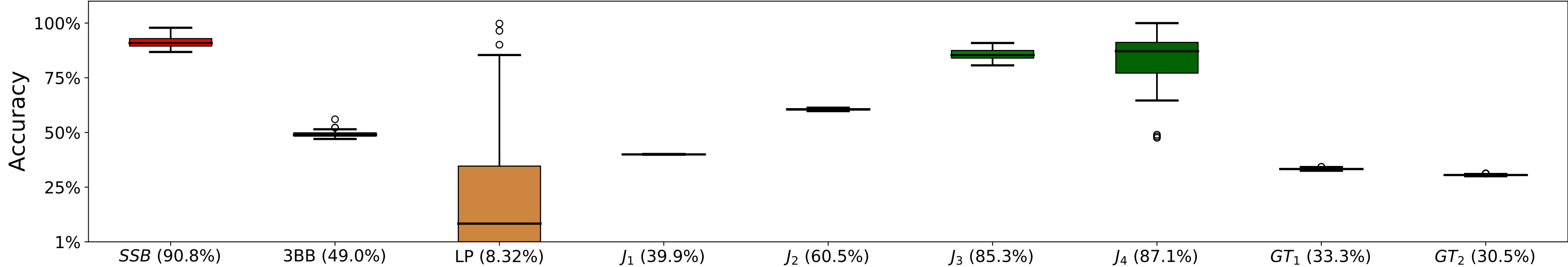

Supplement: Supplemental Material [file UASA_A_2115918_SM5205.zip › supplementary_files/ScaledProcesses/gnomAD/Plots/N_50_nfe_swe.pdf]

Ot. E. As.,  $N = 50$ ,  $M = 7150$

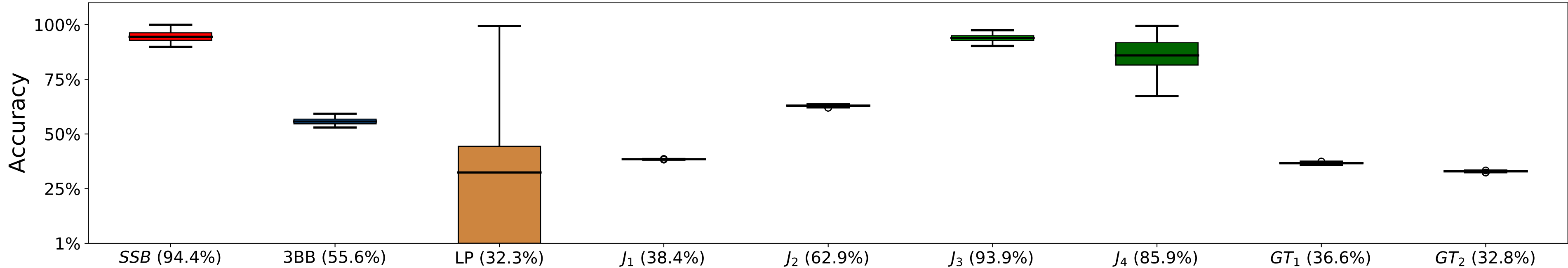

Supplement: Supplemental Material [file UASA_A_2115918_SM5205.zip › supplementary_files/ScaledProcesses/gnomAD/Plots/N_50_eas_oea.pdf]

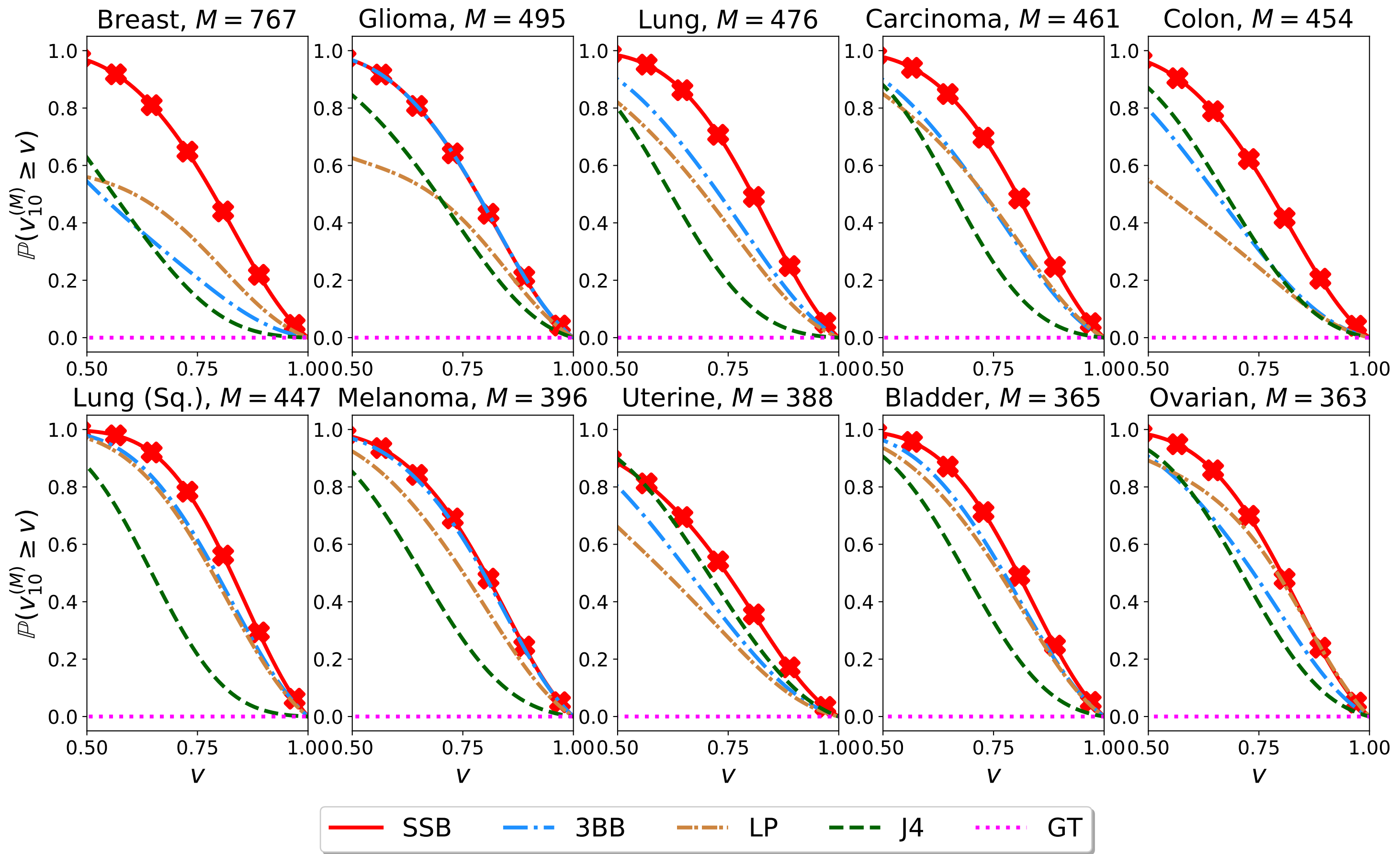

Supplement: Supplemental Material [file UASA_A_2115918_SM5205.zip › supplementary_files/ScaledProcesses/Cancer/Plots/small_N_10_all.pdf]

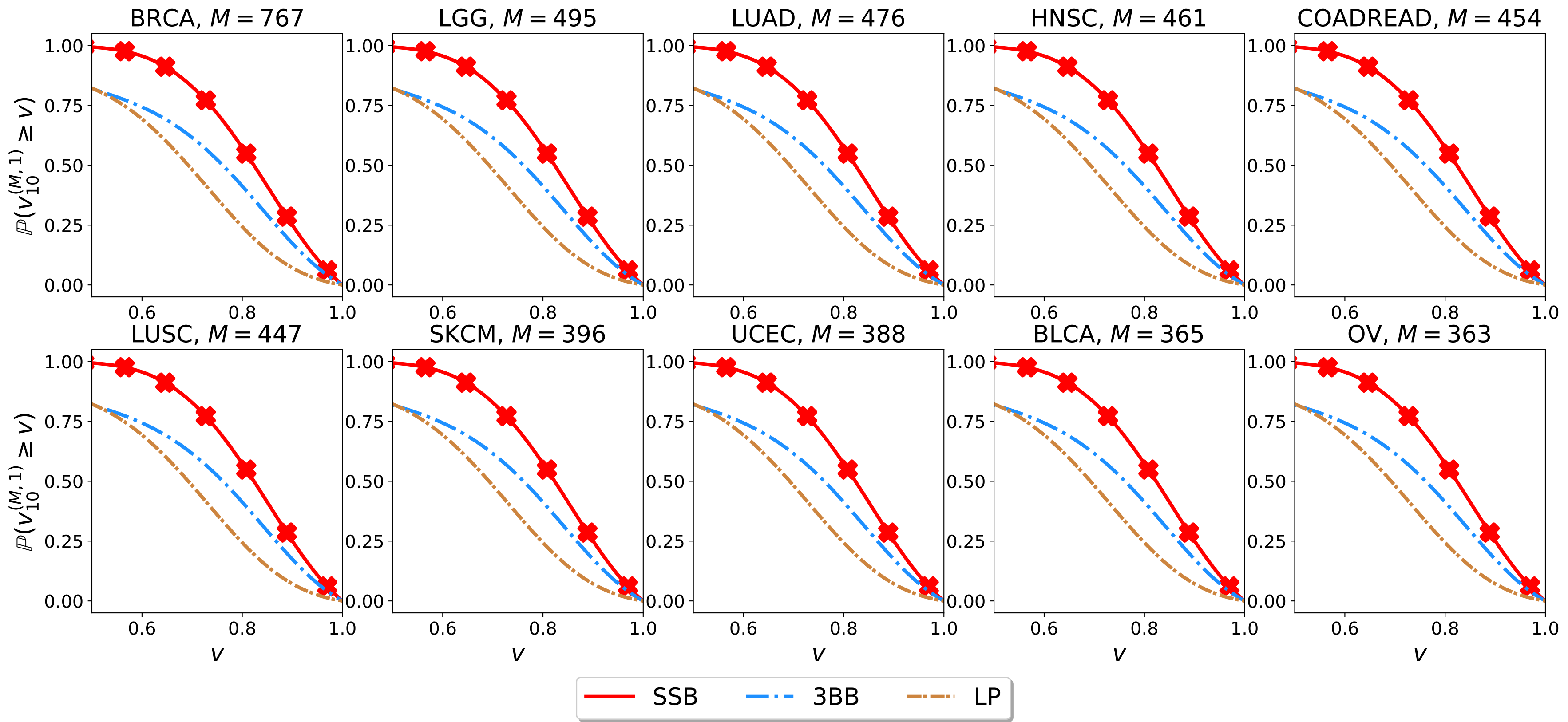

Supplement: Supplemental Material [file UASA_A_2115918_SM5205.zip › supplementary_files/ScaledProcesses/Cancer/Plots/small_N_10_rare.pdf]

$N/(N+M) = 5\%$ 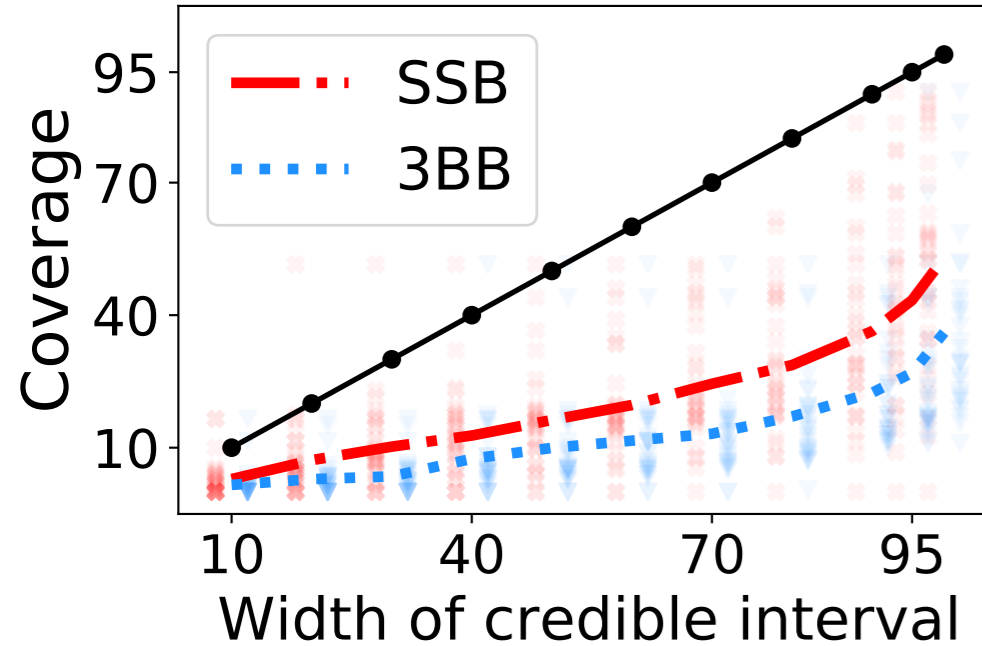 $N/(N+M) = 10\%$ 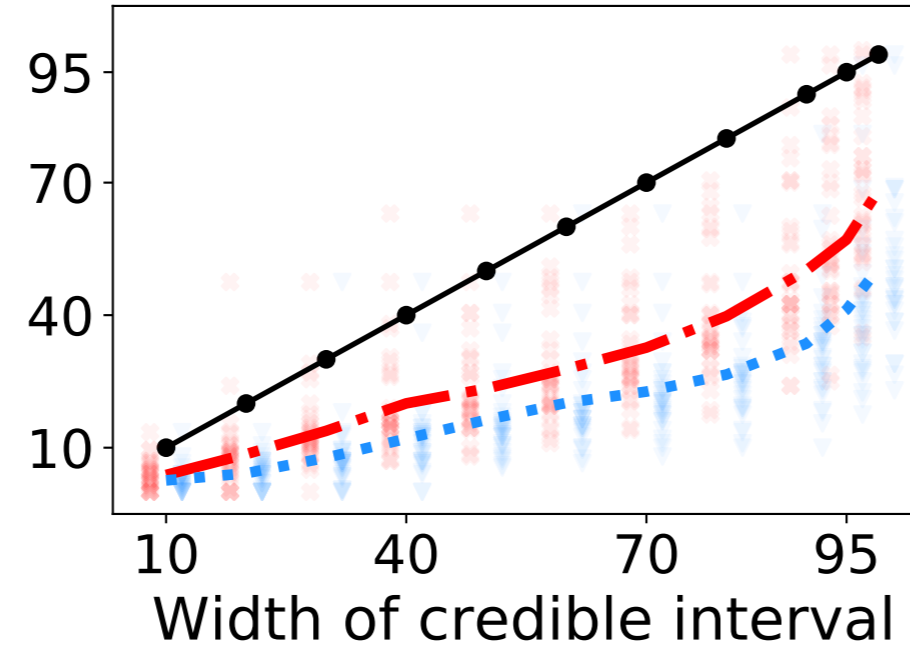 $N/(N+M) = 20\%$ 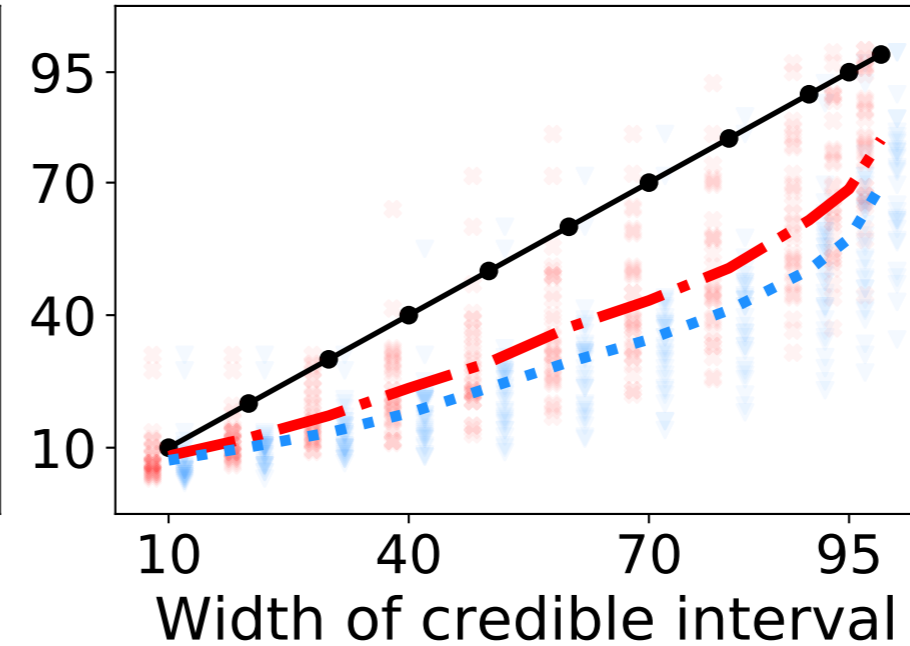 $N/(N+M) = 30\%$ 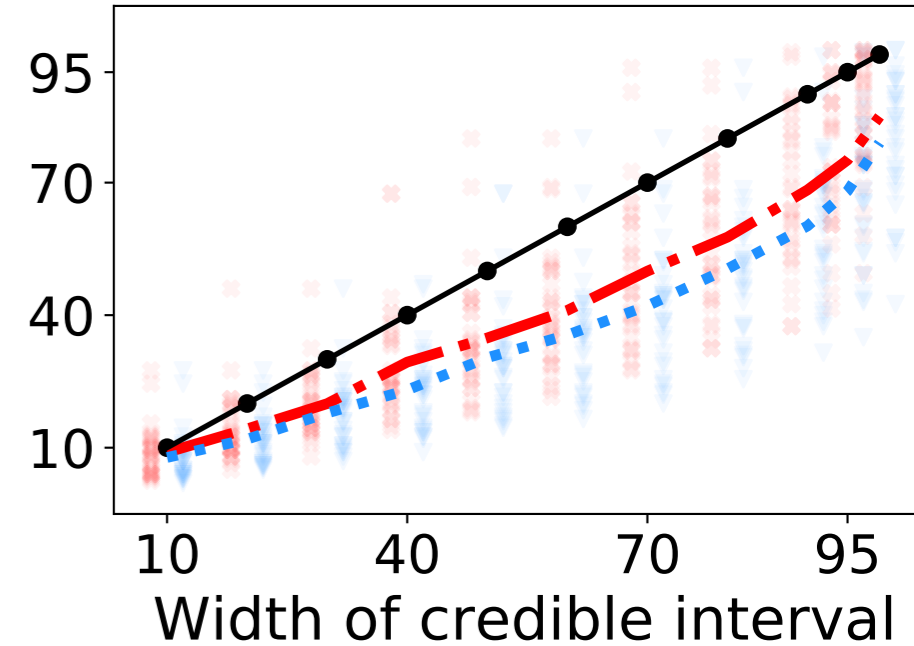

Supplement: Supplemental Material [file UASA_A_2115918_SM5205.zip › supplementary_files/ScaledProcesses/Cancer/Plots/coverage_all.pdf]

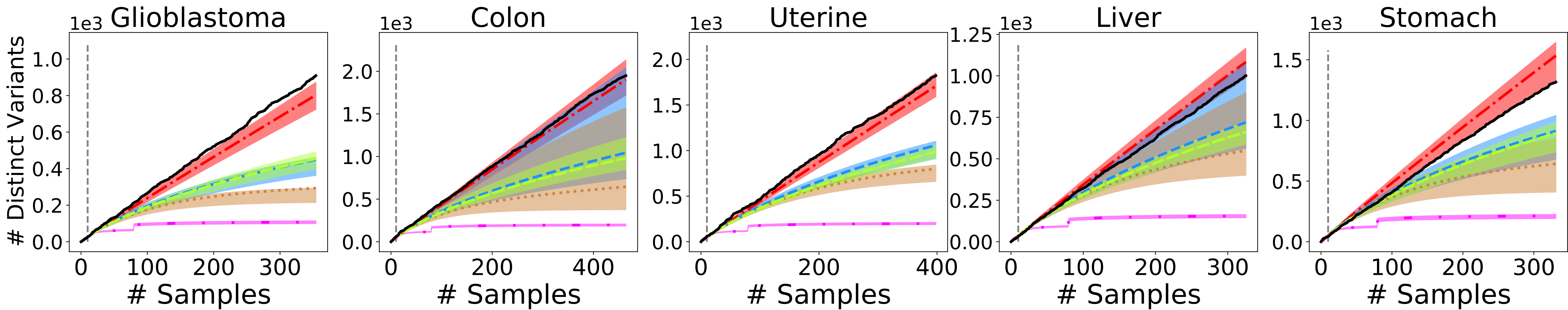

Supplement: Supplemental Material [file UASA_A_2115918_SM5205.zip › supplementary_files/ScaledProcesses/Cancer/Plots/LOO_prediction.pdf]

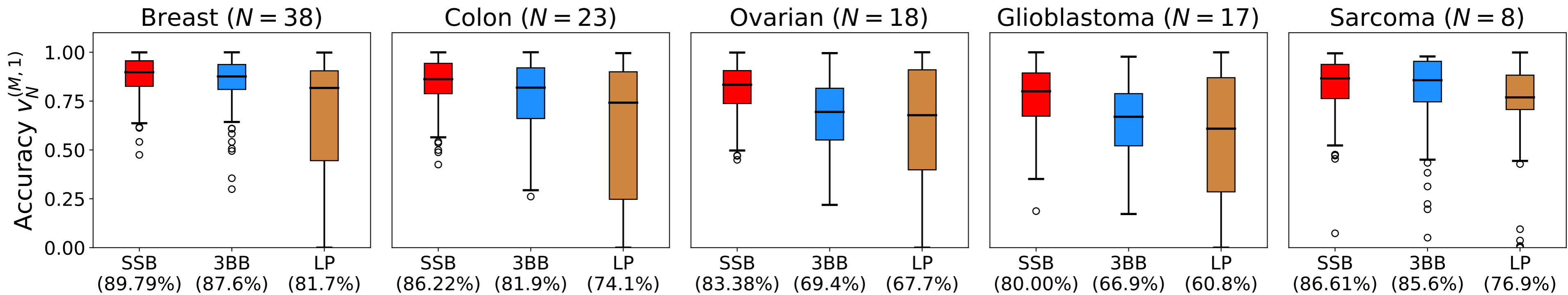

Supplement: Supplemental Material [file UASA_A_2115918_SM5205.zip › supplementary_files/ScaledProcesses/Cancer/Plots/cancer_singletons.pdf]

$$\sigma = 0.7, c = 20$$

# distinct features -  $U_N$

$N = 20$

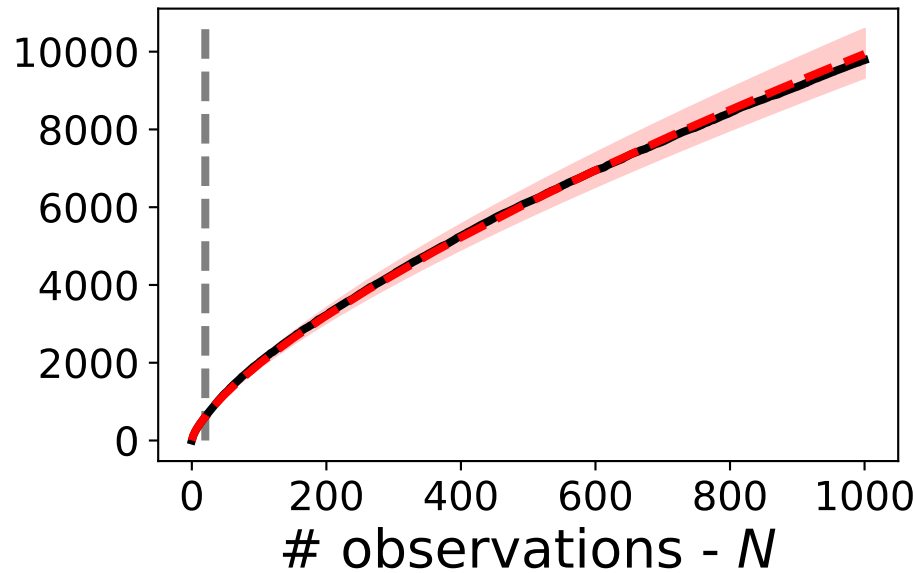

$N = 80$

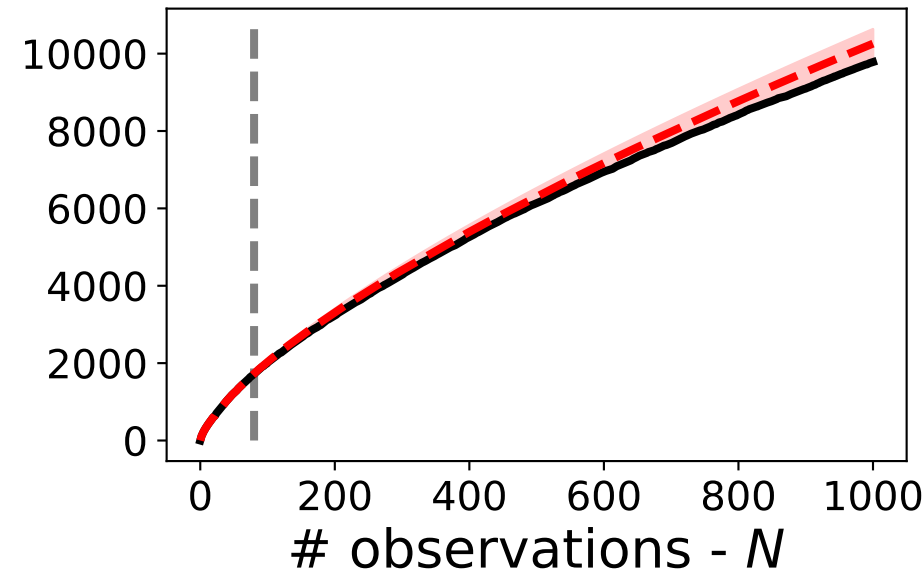

$N = 140$

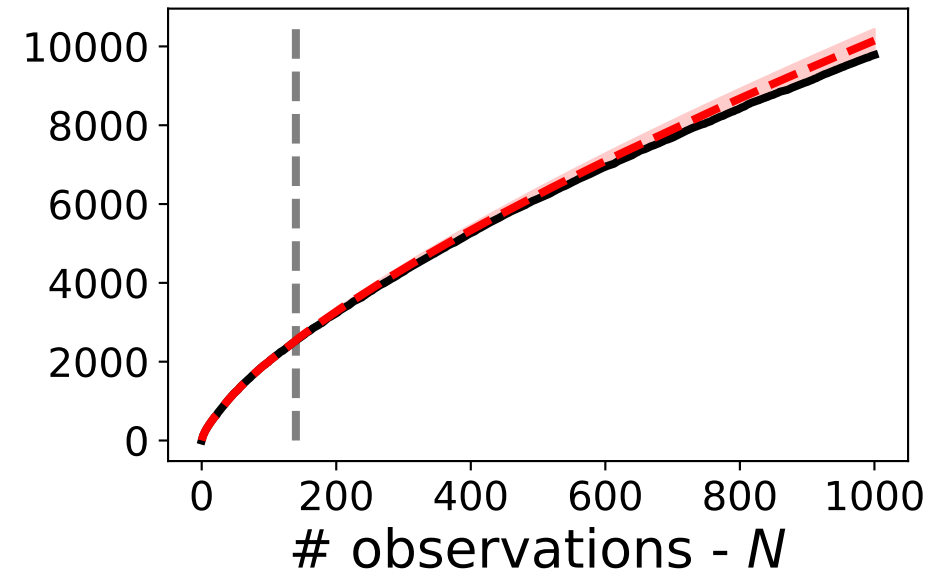

$N = 200$

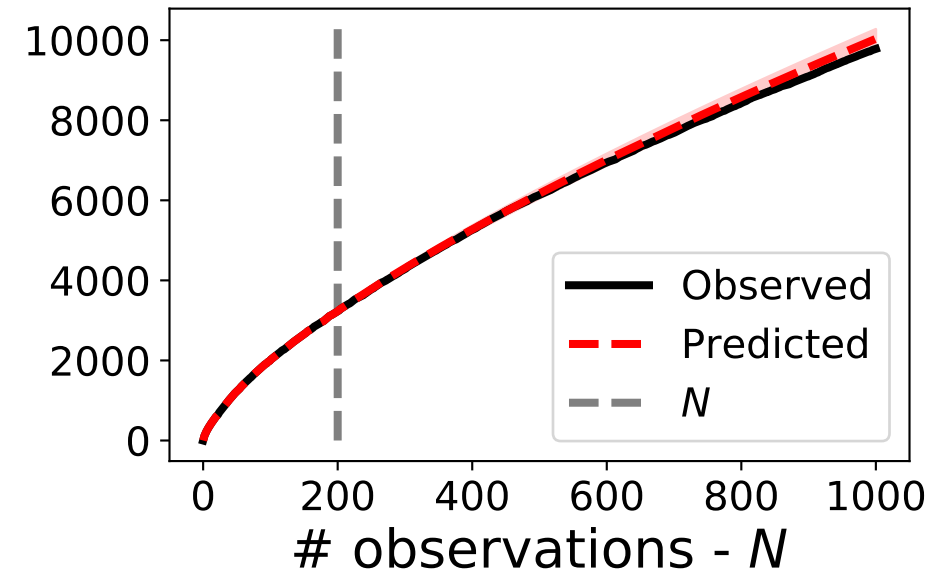

Supplement: Supplemental Material [file UASA_A_2115918_SM5205.zip › supplementary_files/ScaledProcesses/Synthetic/Plots/predictive_4.pdf]

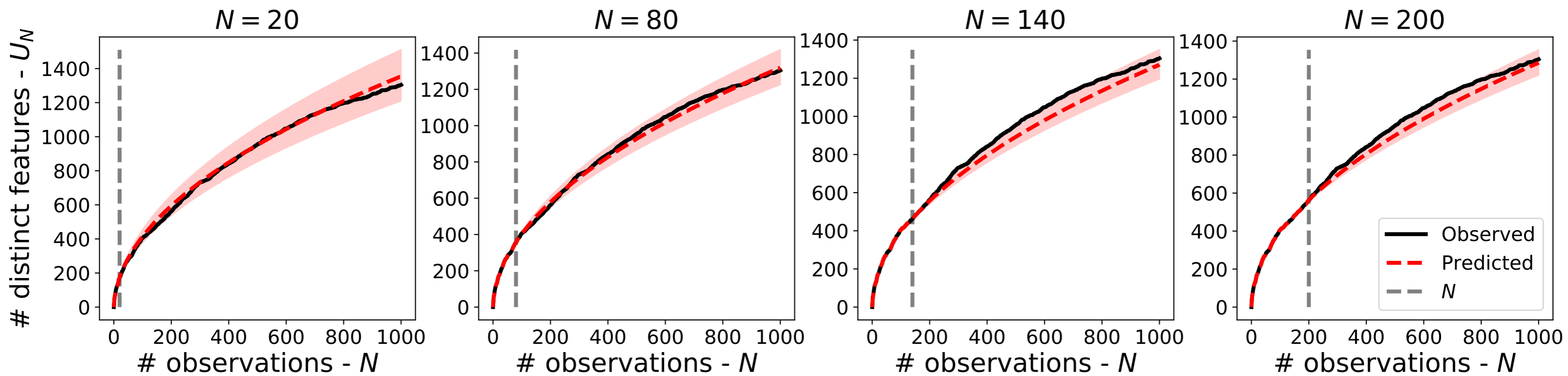

Supplement: Supplemental Material [file UASA_A_2115918_SM5205.zip › supplementary_files/ScaledProcesses/Synthetic/Plots/predictive_2.pdf]

$$\sigma = 0.1, c = 20$$

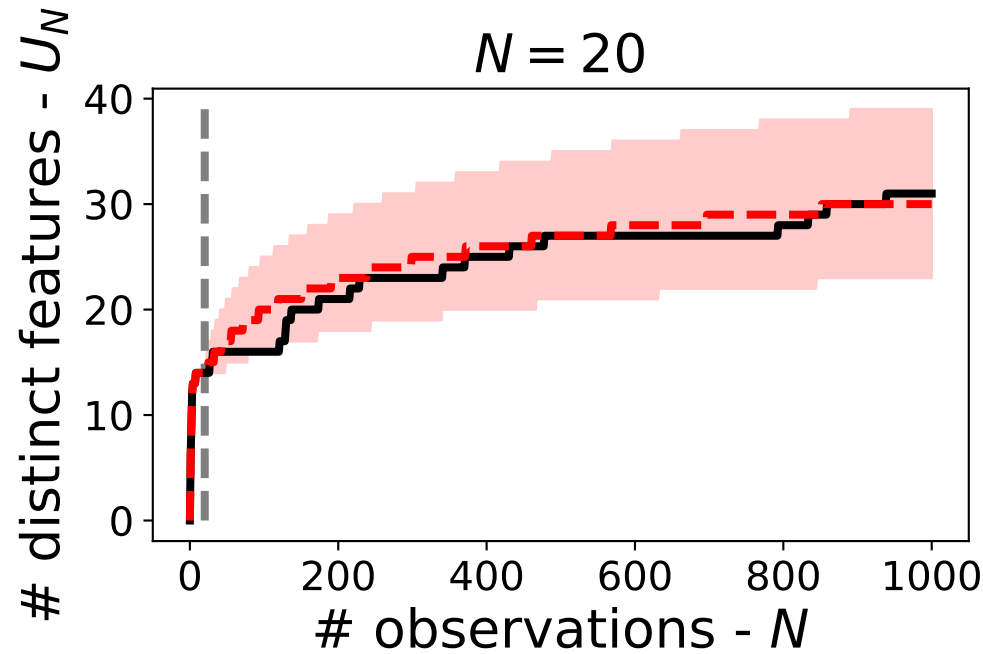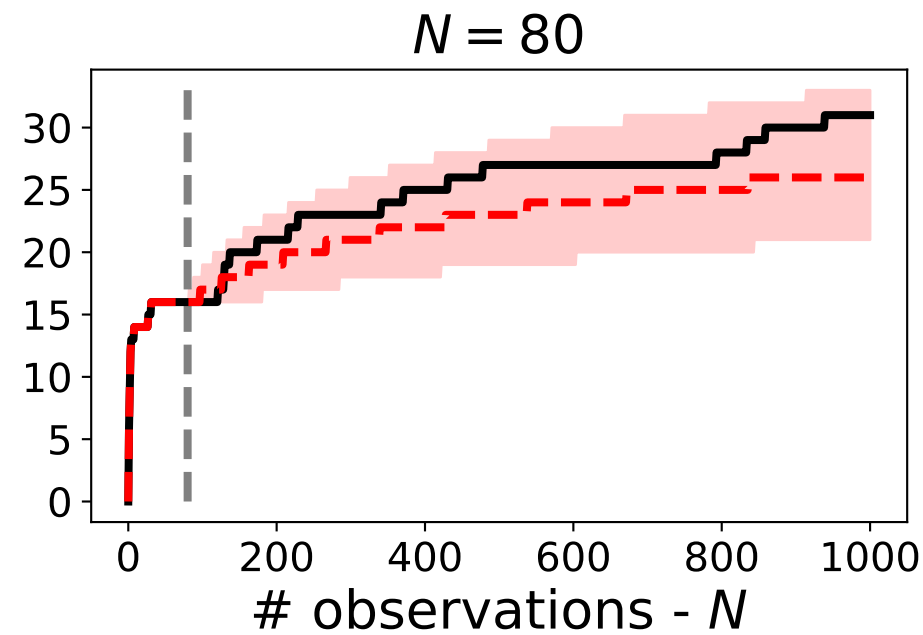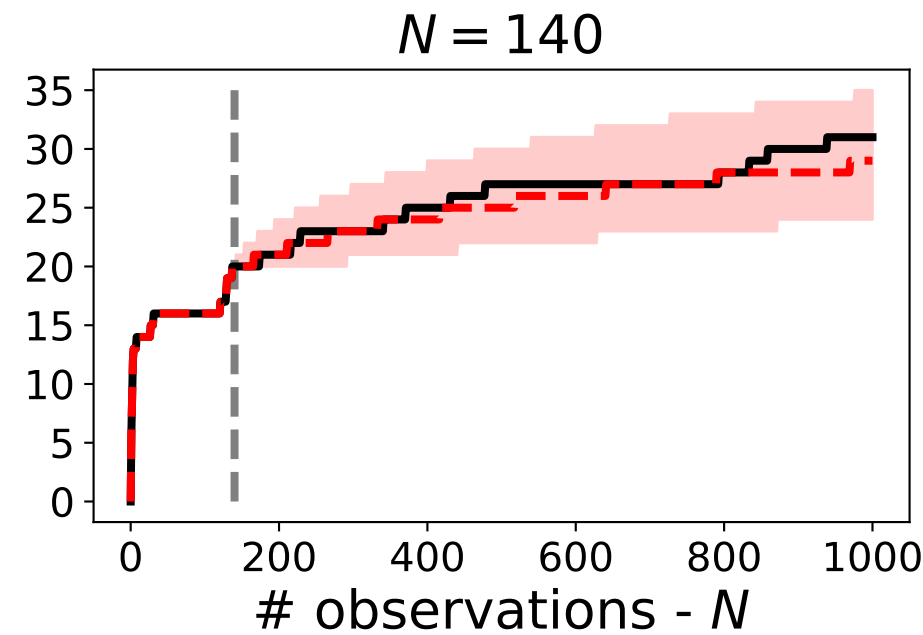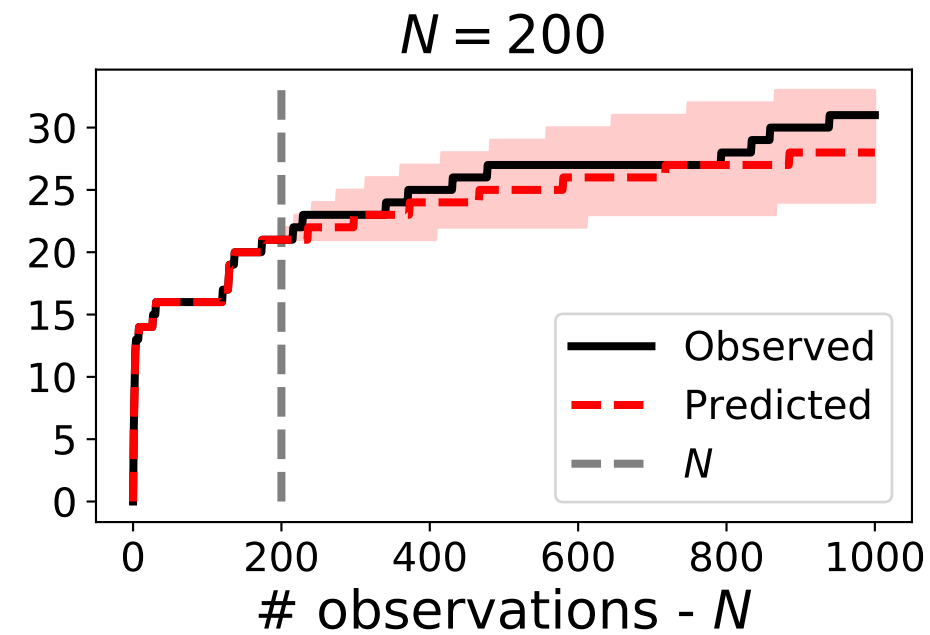

Supplement: Supplemental Material [file UASA_A_2115918_SM5205.zip › supplementary_files/ScaledProcesses/Synthetic/Plots/predictive_3.pdf]

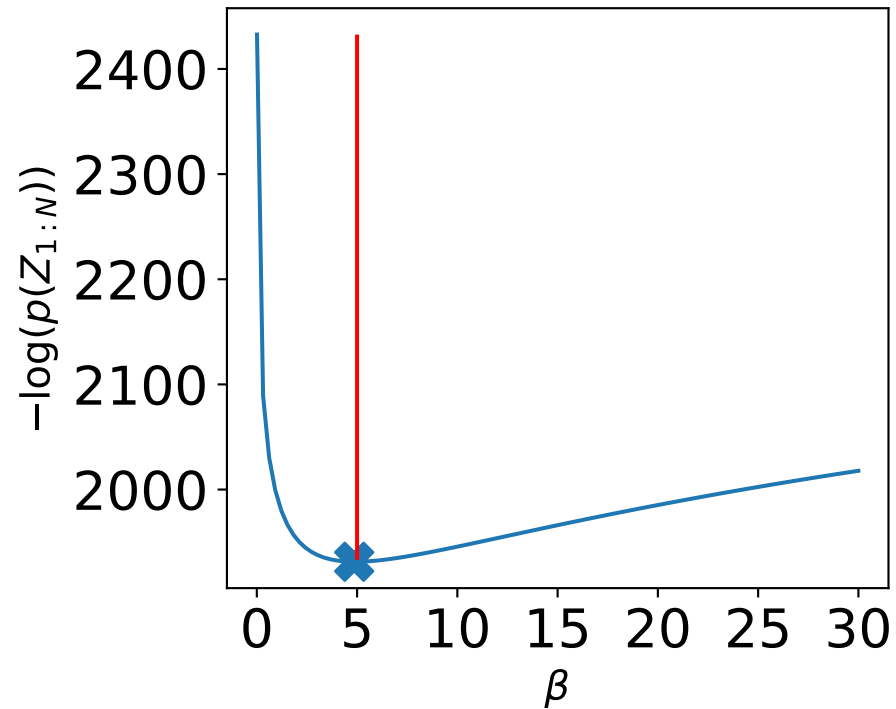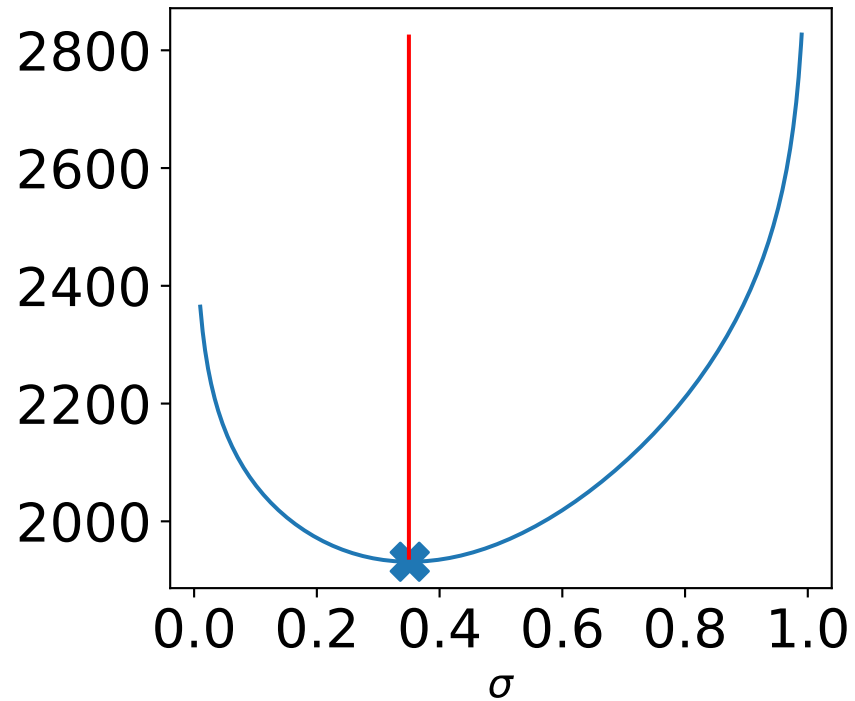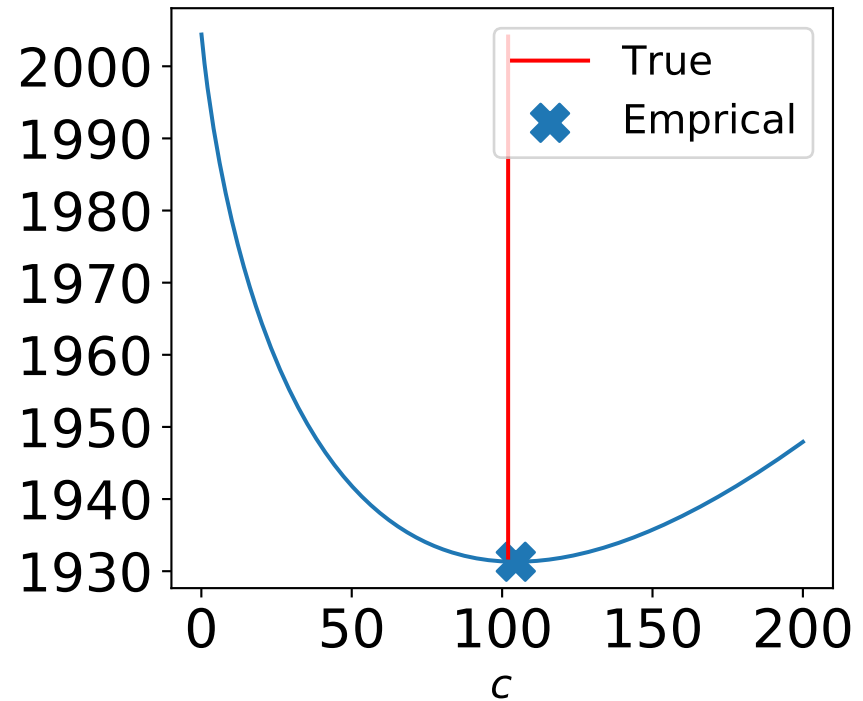

Supplement: Supplemental Material [file UASA_A_2115918_SM5205.zip › supplementary_files/ScaledProcesses/Synthetic/Plots/marginal_like_3.pdf]

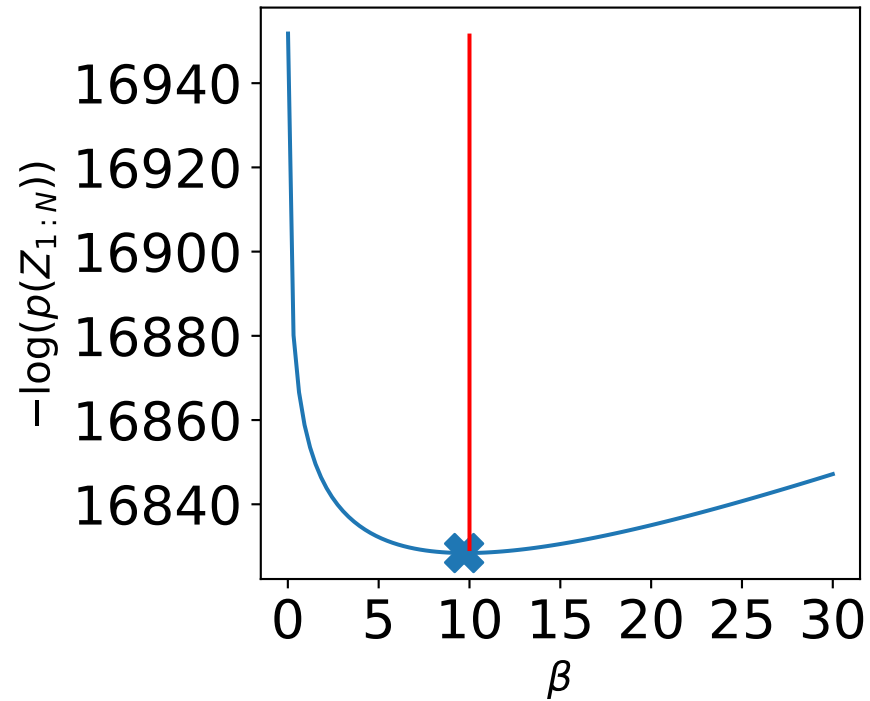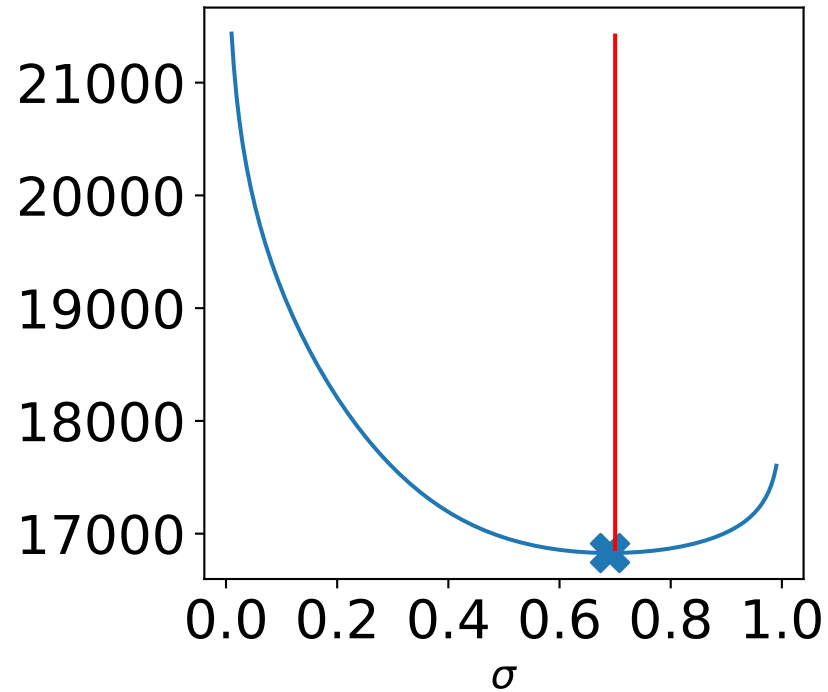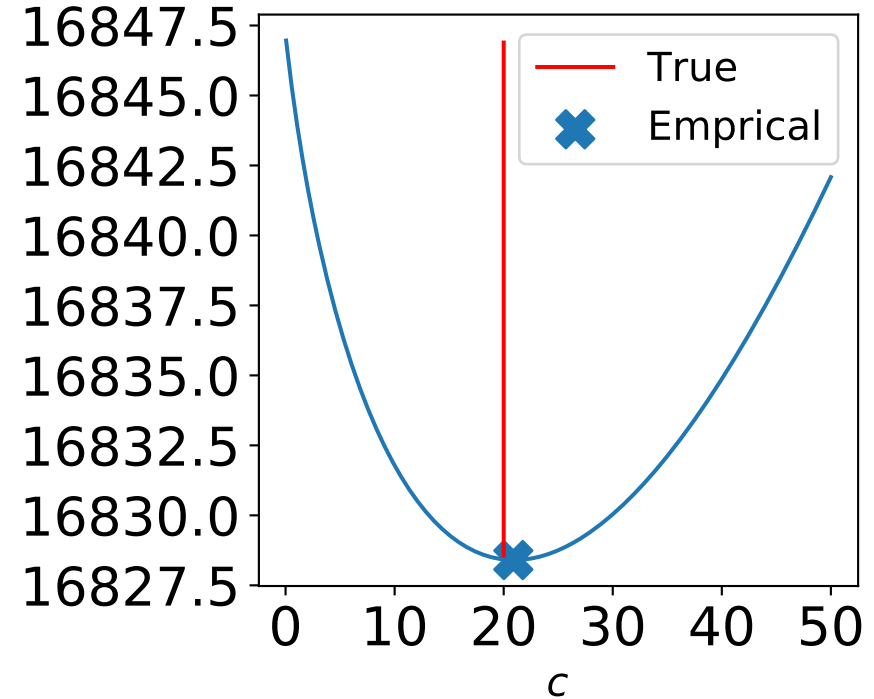

Supplement: Supplemental Material [file UASA_A_2115918_SM5205.zip › supplementary_files/ScaledProcesses/Synthetic/Plots/marginal_like_2.pdf]

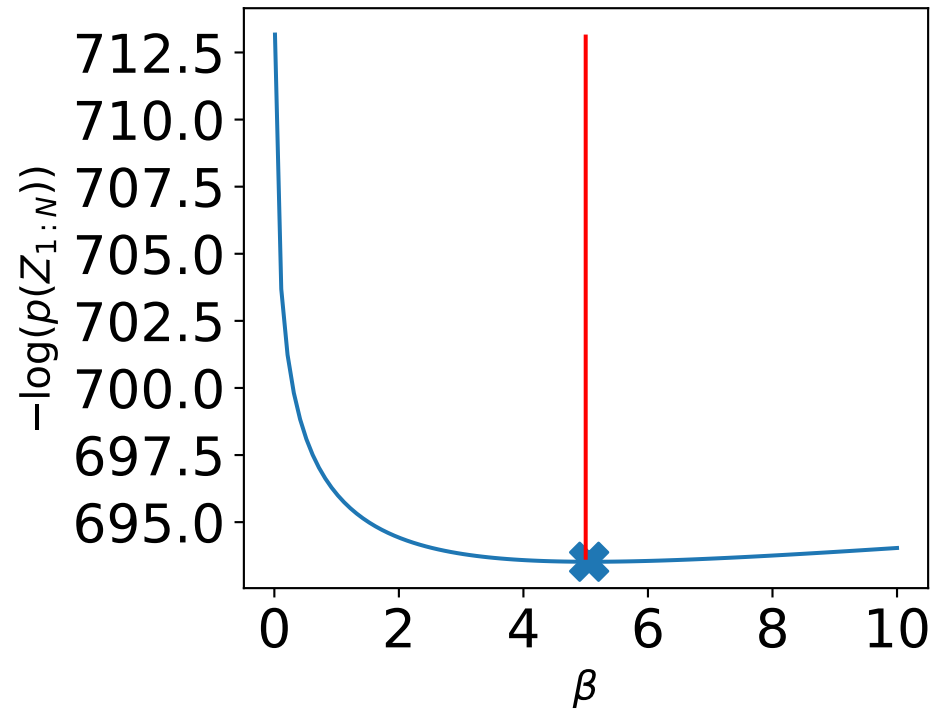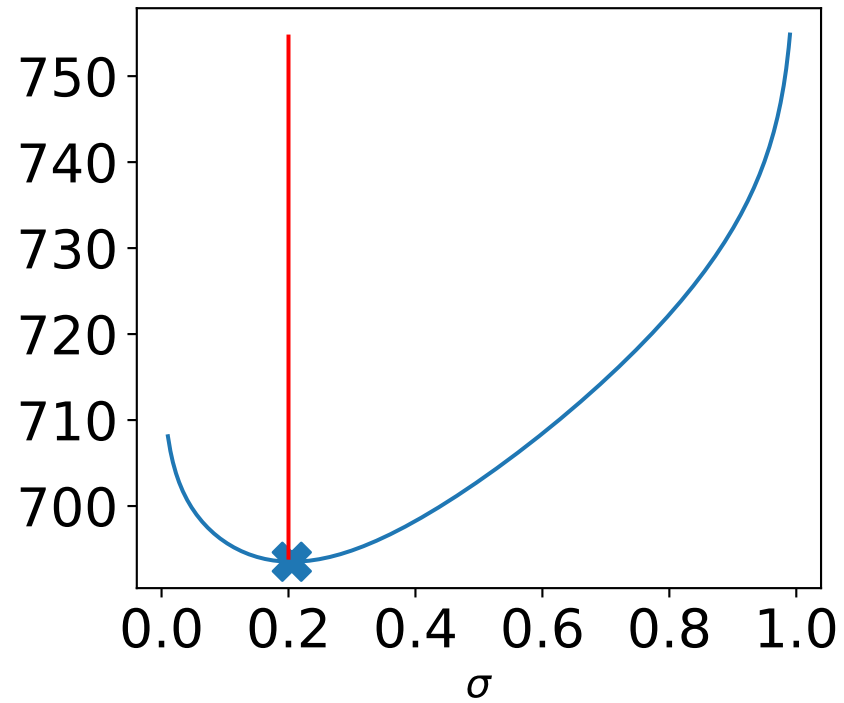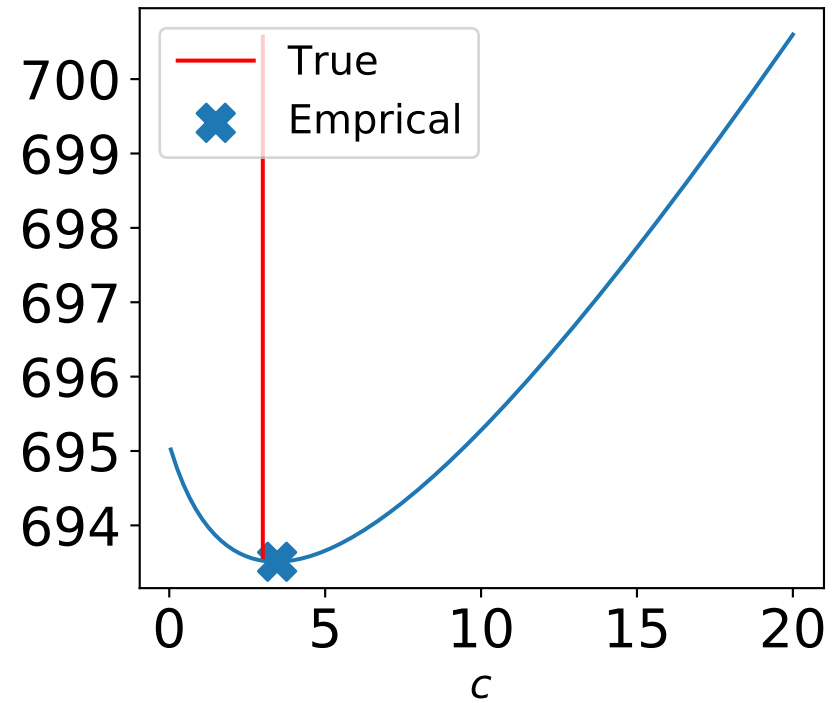

Supplement: Supplemental Material [file UASA_A_2115918_SM5205.zip › supplementary_files/ScaledProcesses/Synthetic/Plots/marginal_like_1.pdf]

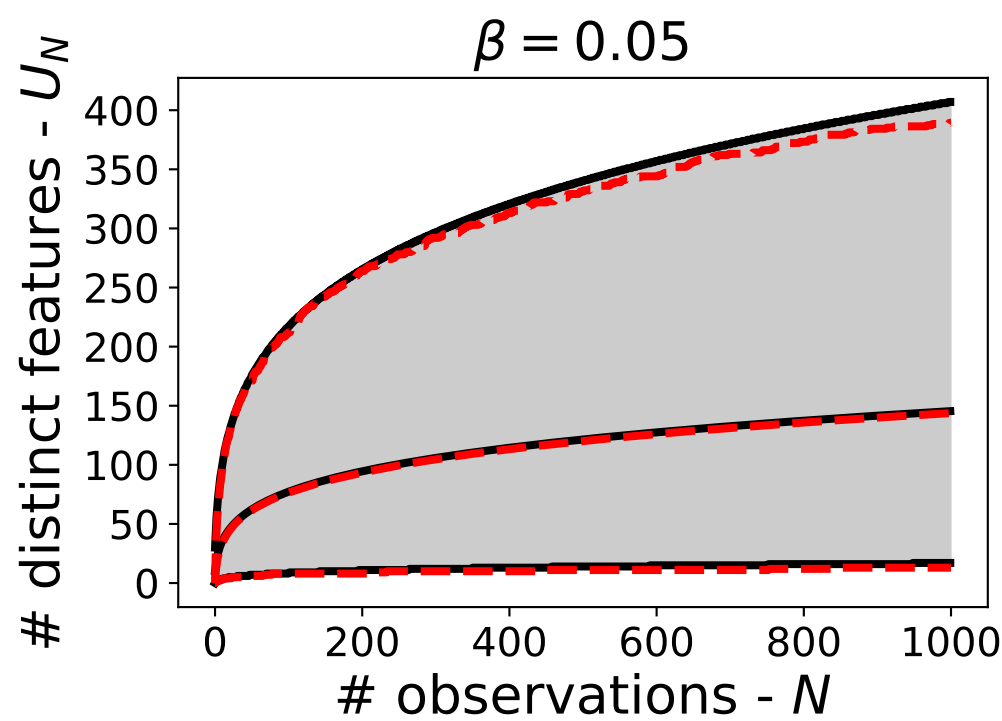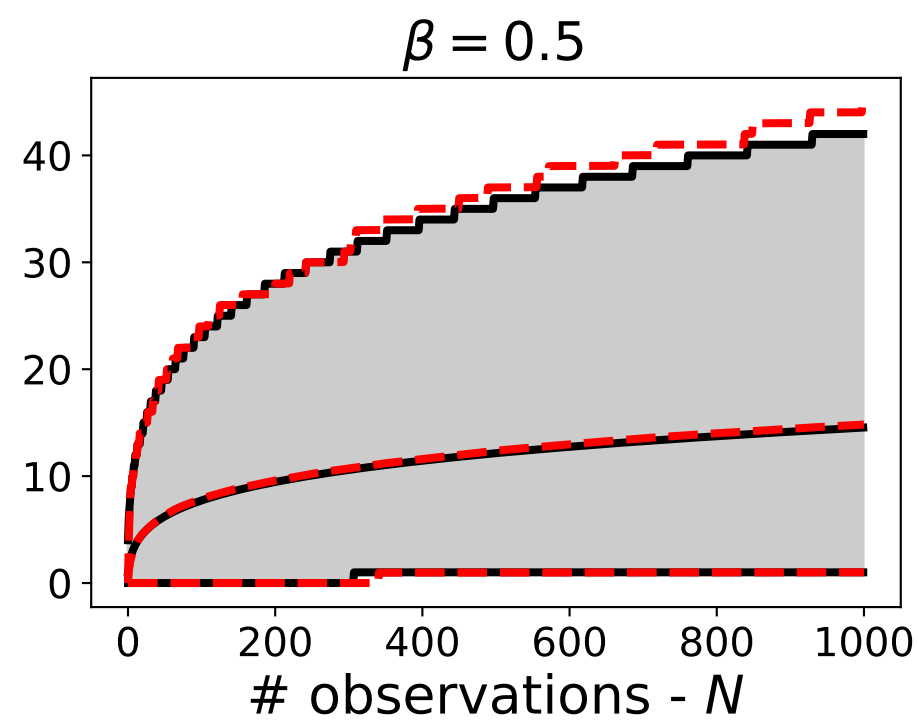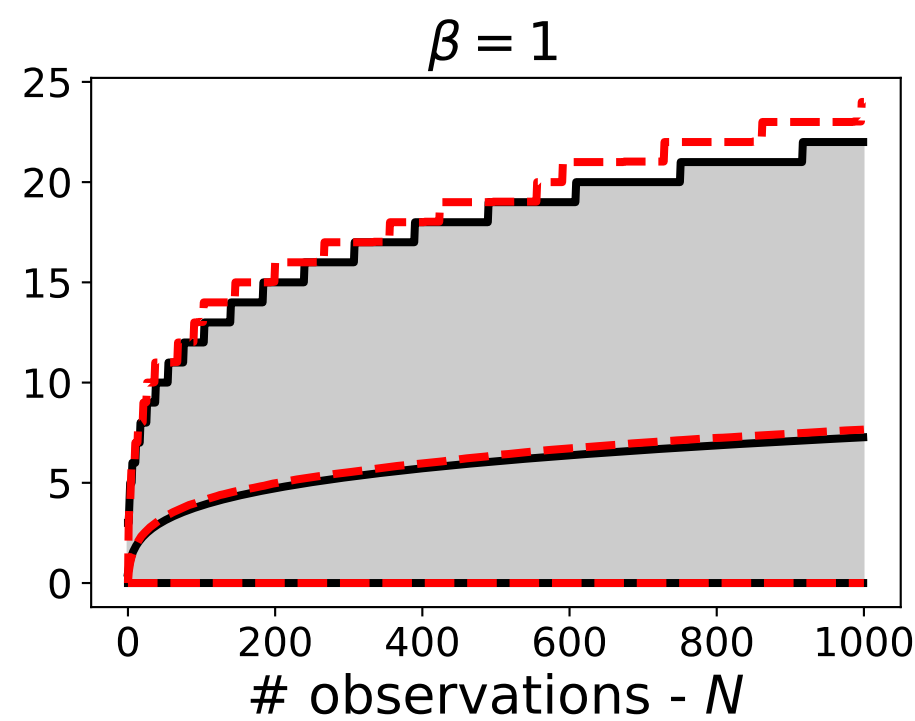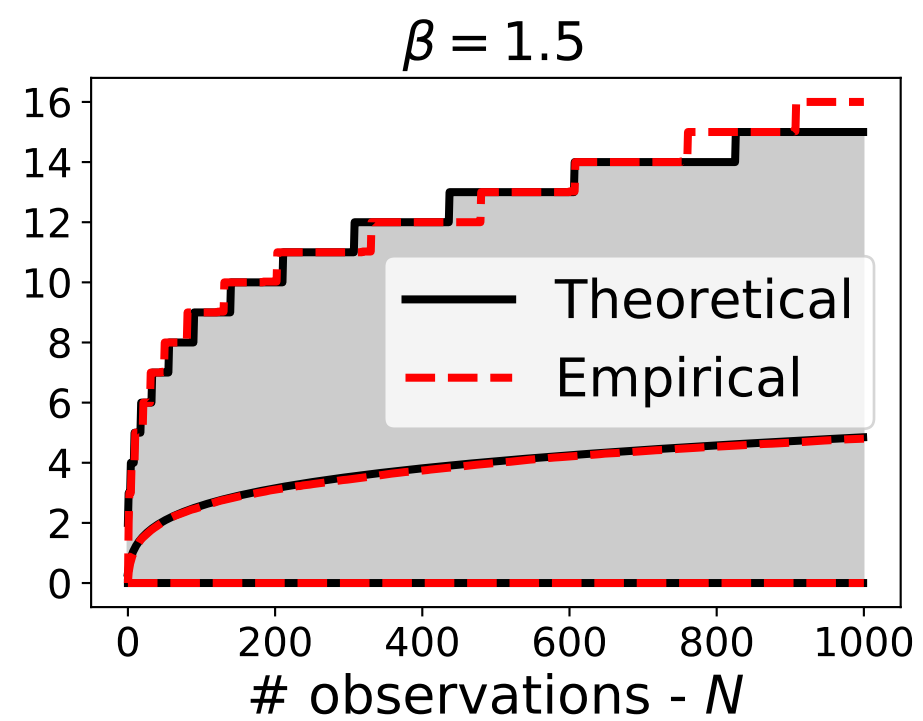

Supplement: Supplemental Material [file UASA_A_2115918_SM5205.zip › supplementary_files/ScaledProcesses/Synthetic/Plots/predictive_beta.pdf]

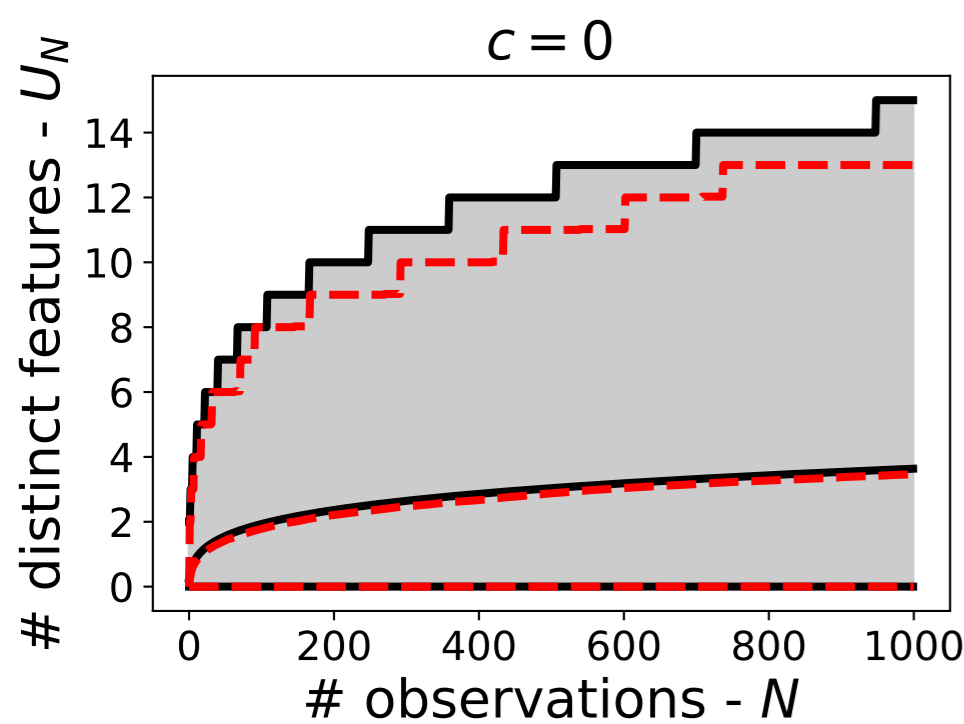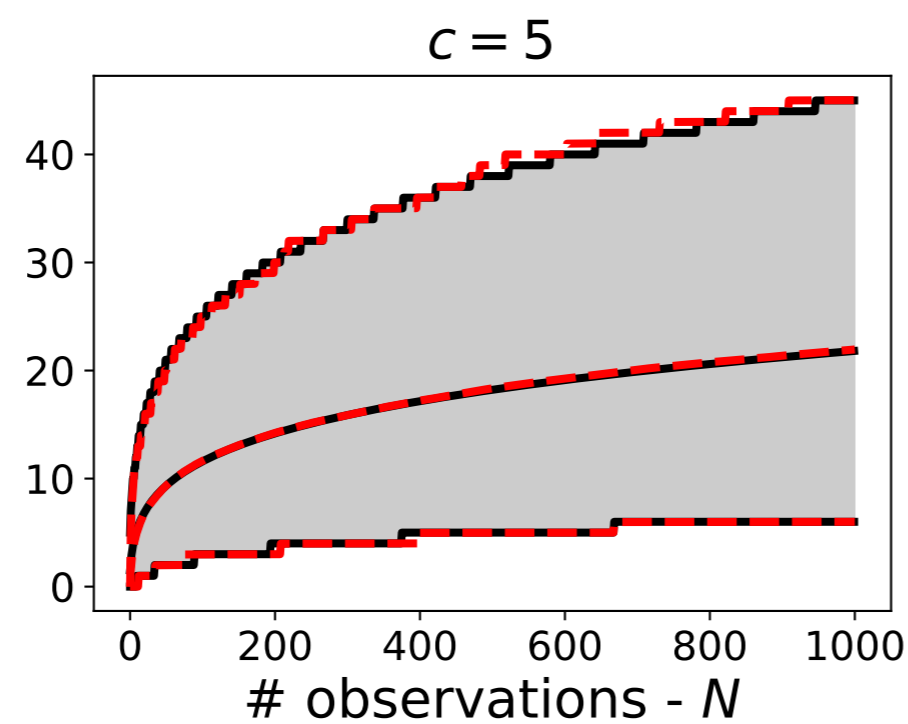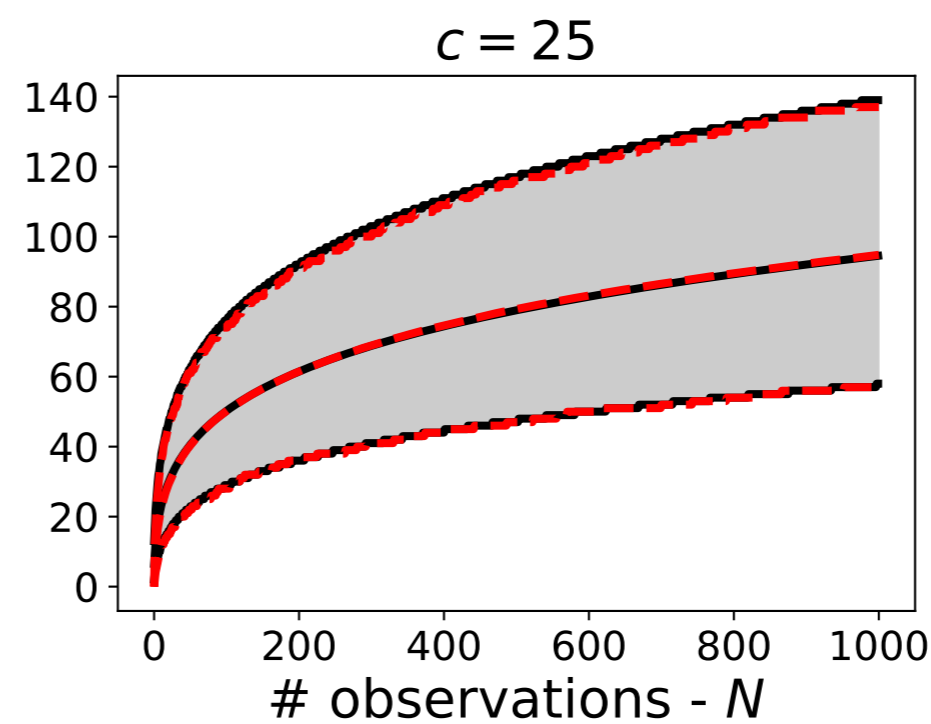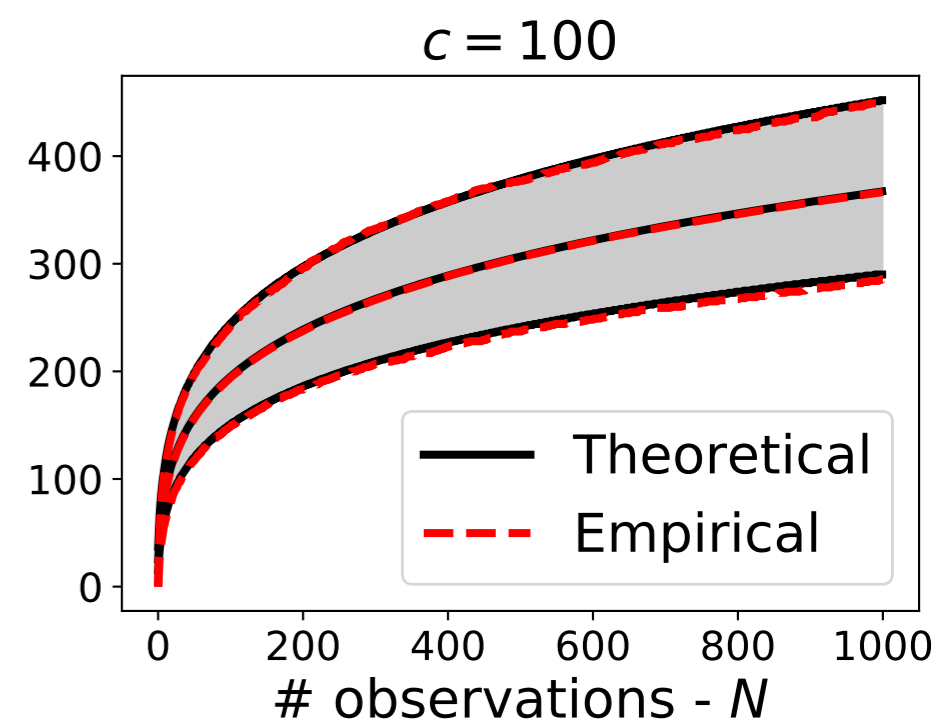

Supplement: Supplemental Material [file UASA_A_2115918_SM5205.zip › supplementary_files/ScaledProcesses/Synthetic/Plots/predictive_tilting.pdf]

$N = 100$ 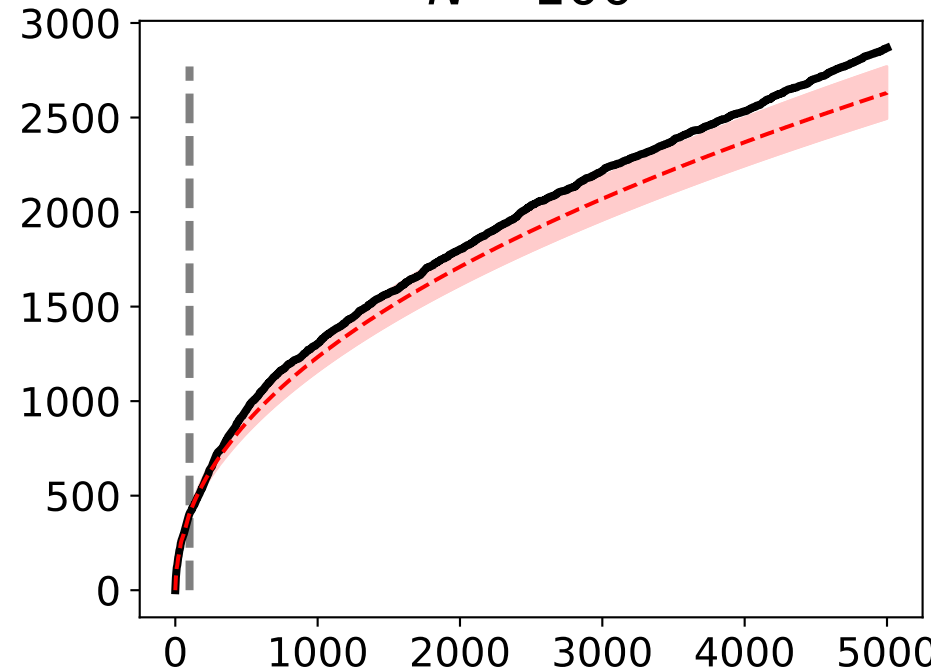 $N = 400$ 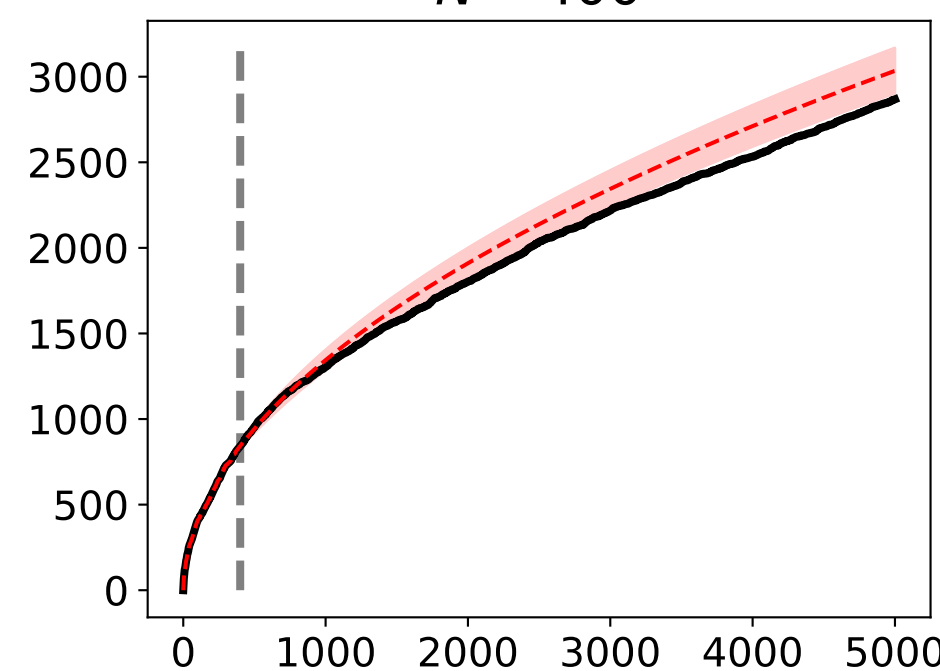 $N = 700$ 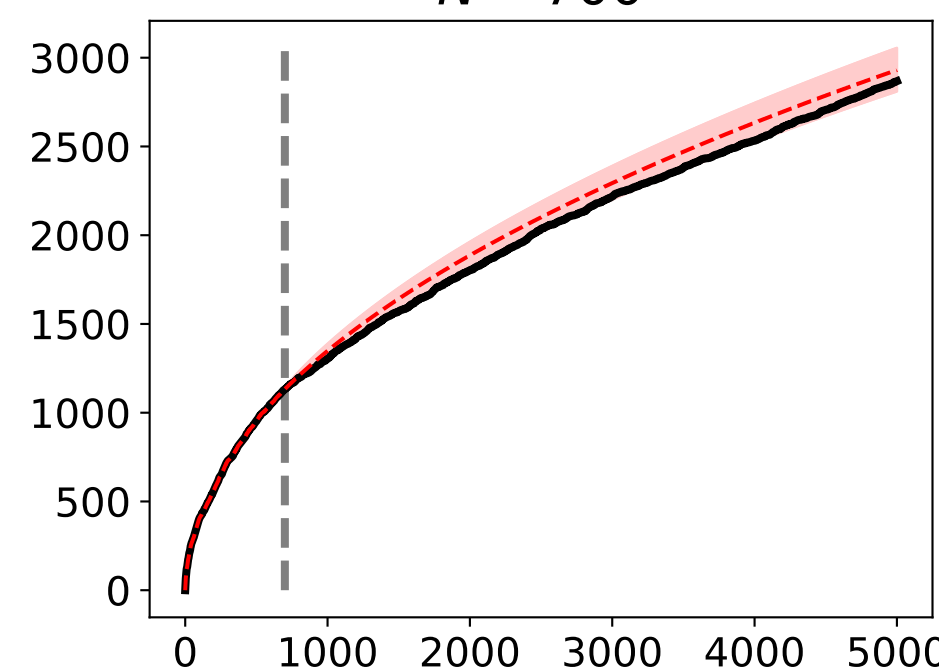 $N = 1000$ 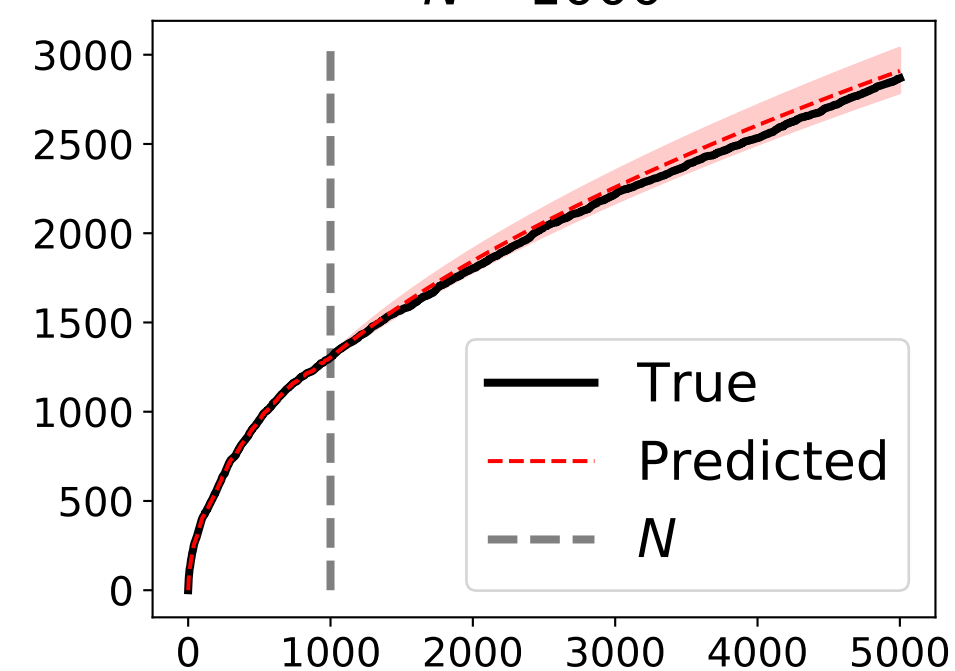

Supplement: Supplemental Material [file UASA_A_2115918_SM5205.zip › supplementary_files/ScaledProcesses/Synthetic/Plots/predicted_learned.pdf]

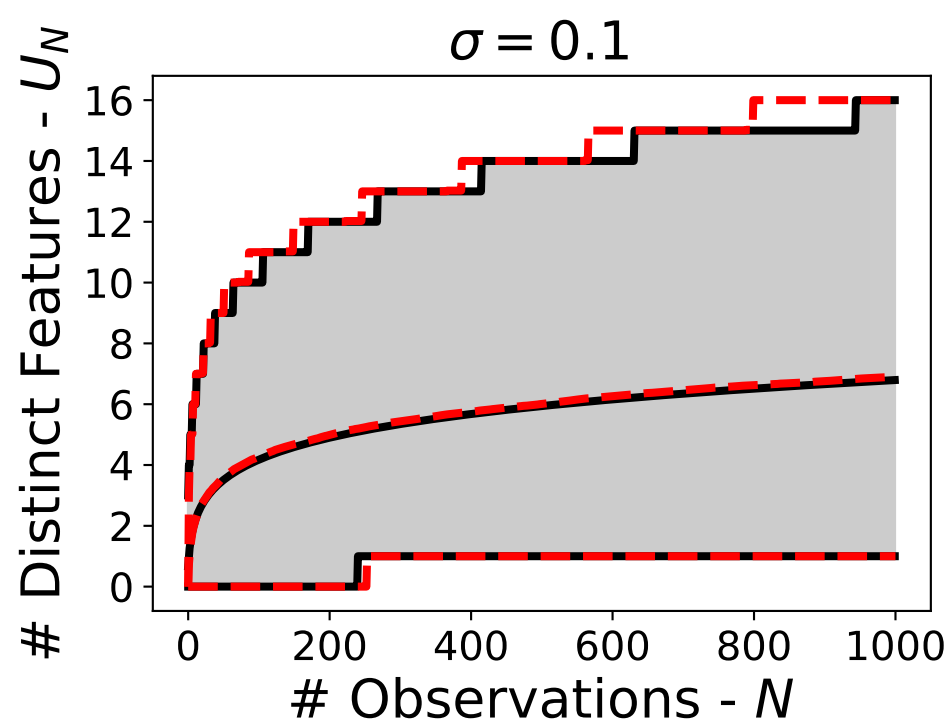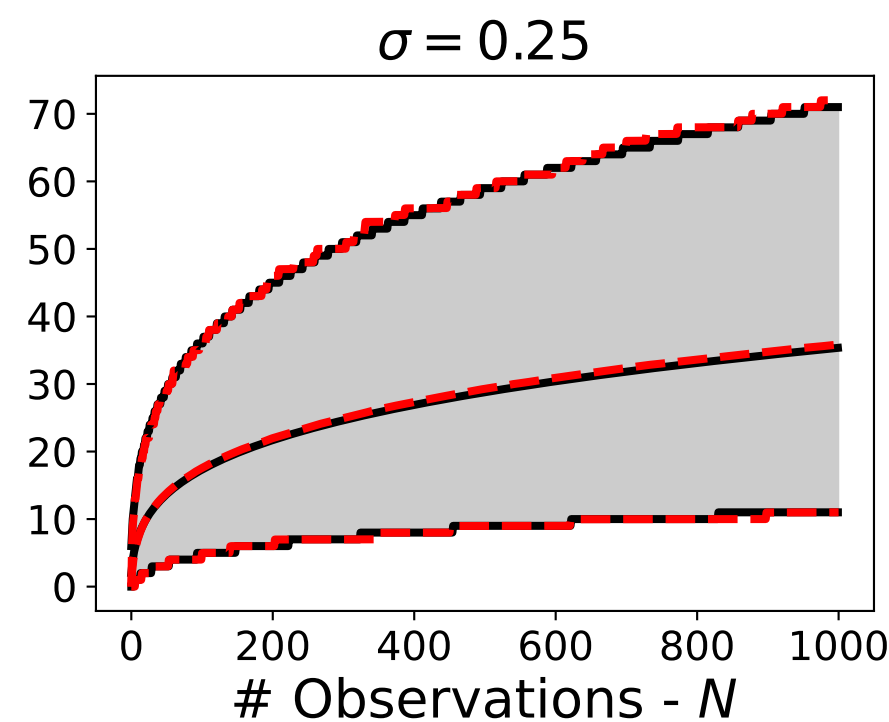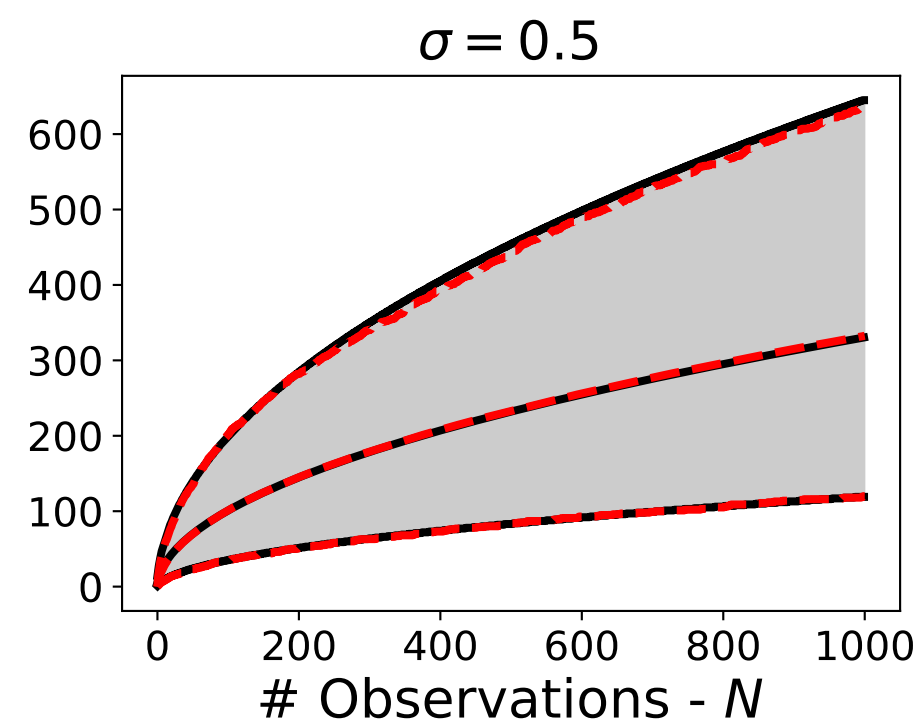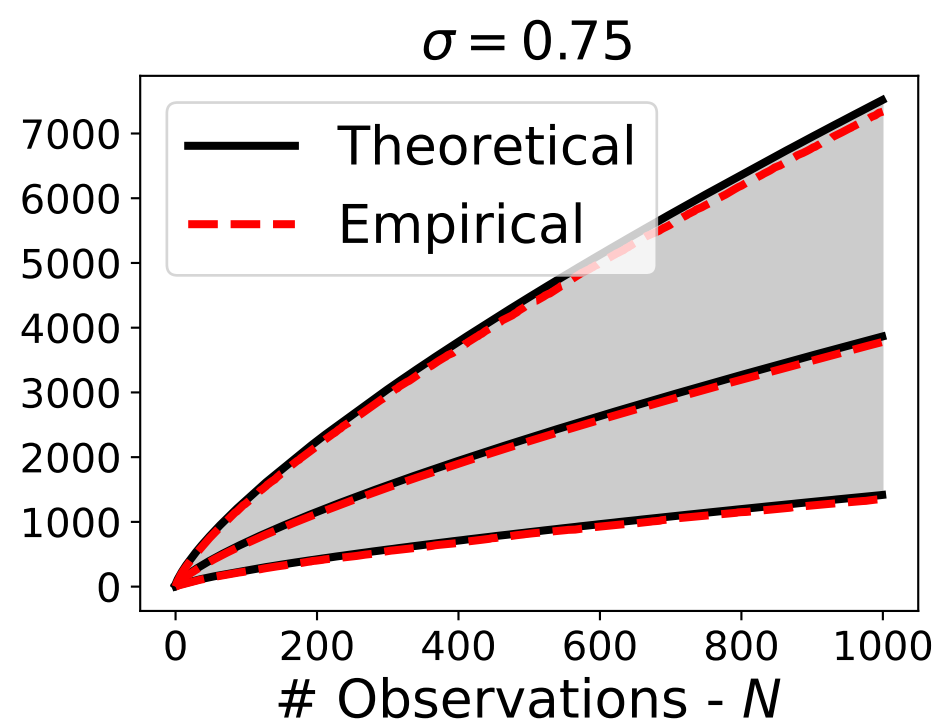

Supplement: Supplemental Material [file UASA_A_2115918_SM5205.zip › supplementary_files/ScaledProcesses/Synthetic/Plots/predictive_sigma.pdf]

$N = 100$ 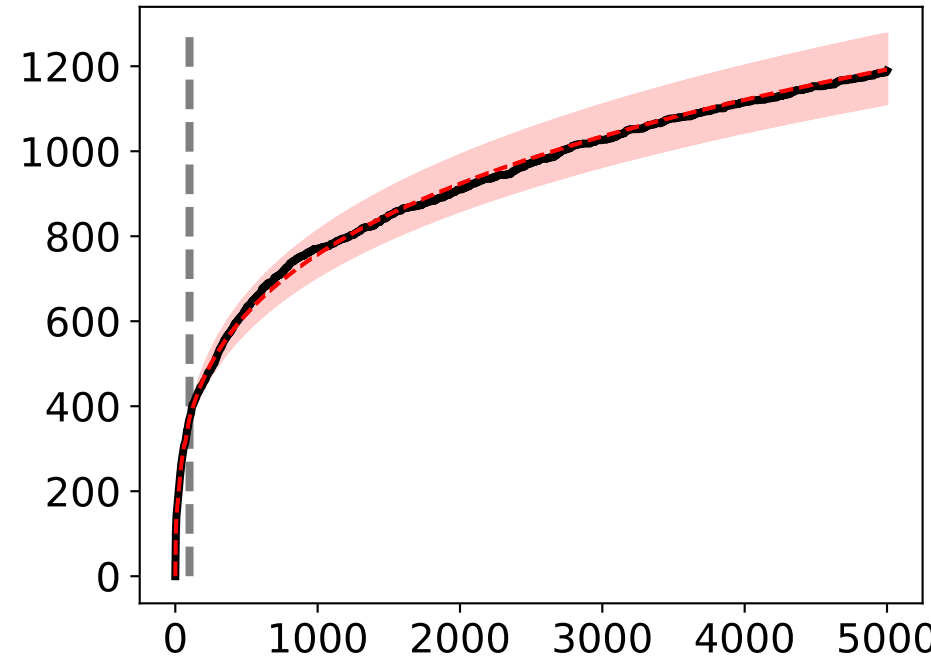 $N = 400$ 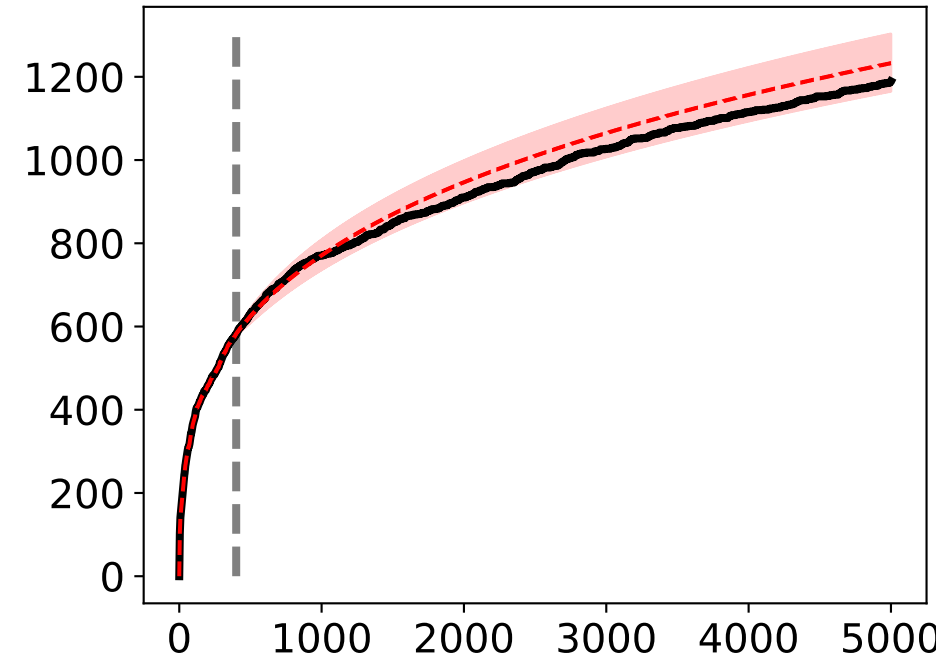 $N = 700$ 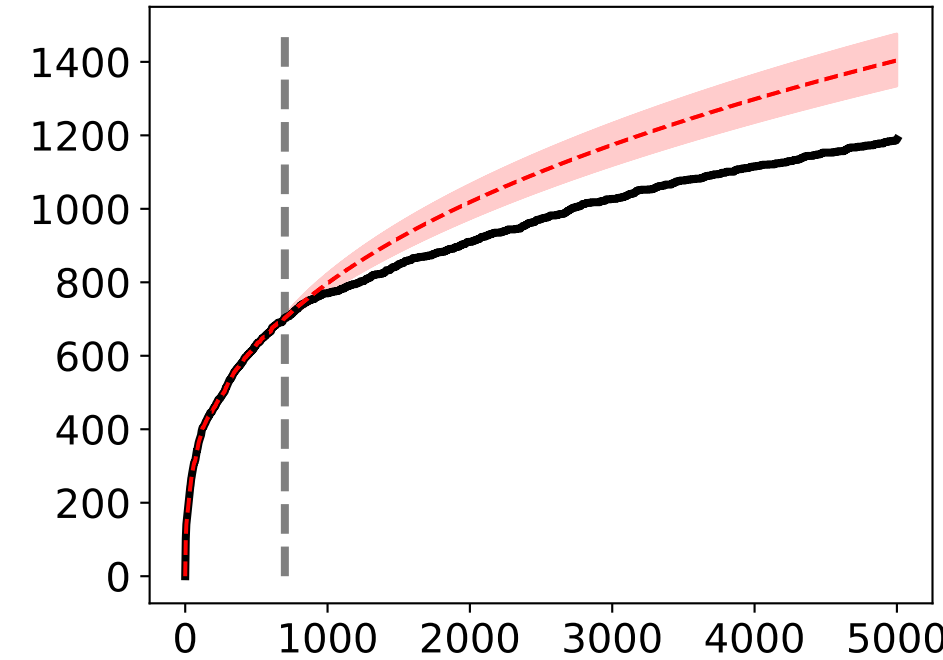 $N = 1000$ 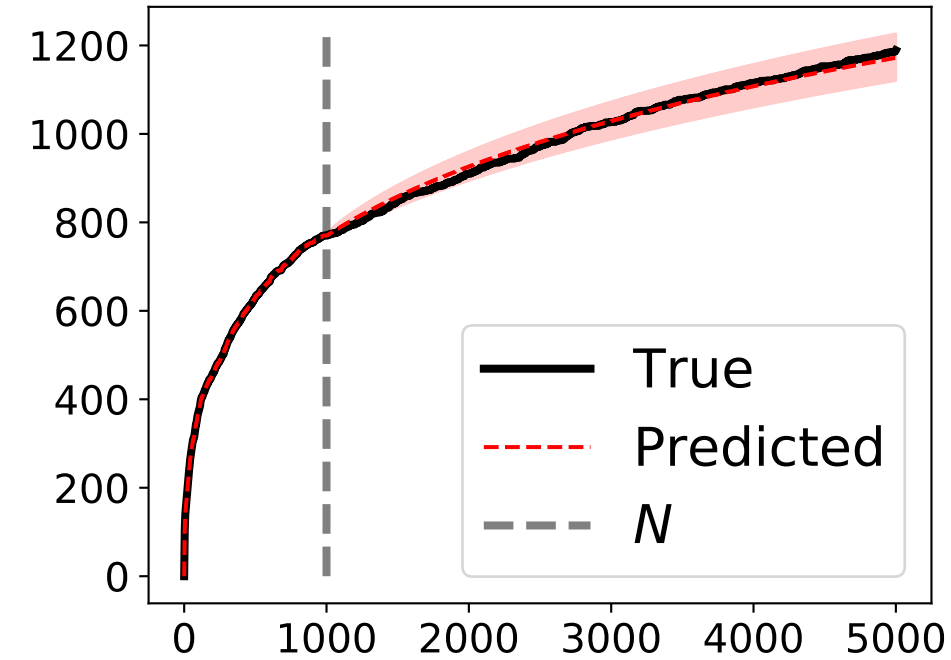

Supplement: Supplemental Material [file UASA_A_2115918_SM5205.zip › supplementary_files/ScaledProcesses/Synthetic/Plots/predicted_learned_2.pdf]
