# Supplementary material for: Scaled Process Priors for Bayesian Nonparametric Estimation of the Unseen Genetic Variation
Source: J Am Stat Assoc. 2022 Sep 29;119(545):320–31. doi: 10.1080/01621459.2022.2115918 (PMC11073059; doi:10.1080/01621459.2022.2115918)
Supplement: Supplemental Material [file UASA_A_2115918_SM5013.docx]

Author Contributions Checklist Form

This form documents the artifacts associated with the article (i.e., the data and code supporting the computational findings) and describes how to reproduce the findings.

# Part 1: Data

This paper **does not** involve analysis of external data (i.e., no data are used or the only data are generated by the authors via simulation in their code).

I certify that the author(s) of the manuscript have legitimate access to and permission to use the data used in this manuscript.

## Abstract

In the manuscript, we use both synthetic and real genomic cancer data, readily and freely available online (TCGA and msk-impact cancer datasets).

We provide the processed data we use in our experiments at https://github.com/lorenzomasoero/ScaledProcesses

## Availability

Data **are** publicly available

Data **cannot be made** publicly available

If the data are publicly available, see the *Publicly available data* section. Otherwise, see the *Non-publicly available dat*a section, below.

### Publicly available data

Data are available online at: https://portal.gdc.cancer.gov/ and http://cbioportal.org/msk-impact

Data are available as part of the paper’s supplementary material.

Data are publicly available by request, following the process described here:

We also attach the data used

Data are or will be made available through some other mechanism, described here:

### Non-publicly available data

Discussion of lack of publicly available data:

## Description

### File format(s)

CSV or other plain text:

Software-specific binary format (.Rda, Python pickle, etc.):

Standardized binary format (e.g., netCDF, HDF5, etc.):

Other (described here):

### Data dictionary

Provided by the authors in the following file(s):

Data file(s) is (are) self-describiing (e.g., netCDF files)

Available at the following URL:

https://github.com/lorenzomasoero/ScaledProcesses

### Additional information (optional)

Click or tap here to enter text.

# Part 2: Code

## Abstract

We provide jupyter notebook files to reproduce the analysis and plots at https://github.com/lorenzomasoero/ScaledProcesses

## Description

### Code format(s)

Script files

R  Python  Matlab

Other:

Package

R  Python  MATLAB toolbox

Other:

Reproducible report

R Markdown  Jupyter notebook

Other:

Shell script

Other (described here):

### **Supporting software requirements**

Version of primary software used

Python 3

Libraries and dependencies used by the code

Numpy, scipy, matploltib,

### Supporting system/hardware requirements (optional)

### Parallelization used

No parallel code used

Multi-core parallelization on a single machine/node

Number of cores used:

Multi-machine/multi-node parallelization

Number of nodes and cores used:

### License

MIT License (default)

BSD

GPL v3.0

Creative Commons

Other (described here):

### Additional information (optional)

# Part 3: Reproducibility workflow

## Scope

The provided workflow reproduces:

Any numbers provided in text in the paper

The computational method(s) presented in the paper (i.e., code is provided that implements the method(s))

All tables and figures in the paper

Selected tables and figures in the paper, as explained and justified here:

## Workflow details

### Format(s)

Single master code file

Wrapper (shell) script(s)

Self-contained R Markdown file, Jupyter notebook, or other literate programming approach

Text file (e.g., a readme-style file) that documents workflow

Makefile

Other (more detail in 'Instructions' below)

### Instructions

The repository is divided into 4 main folders:

- `utils_folder/` which contains all the code and functions to replicate the analysis. In particular, each method considered has its own `.py` file.
- `Synthetic/` which contains example usage on synthetic datasets
- `Cancer/` which contains data and code to run and fit models on TCGA cancer data, and reproduce plots.
- `gnomAD/` which contains data and code to run and fit models on the gnomAD dataset, and reproduce plots.

In `Cancer/`, `gnomAD/` and `Synthetic/` you will find `Fit.ipynb`, an iPythonNotebook which contains all the code needed in order to fit the experiments and save the data necessary to then reproduce the plots. Notice: `Synthetic/Fit.ipynb` also contains code to produce figures for the syntetic data. The relevant functions called to fit the methods can be found in the `utils/` folder.

In `Cancer/` and `gnomAD/` you will find `Plots.ipynb`, an iPythonNotebook which contains all the code needed in order to produce the plots displayed in the paper.

* `Cancer/Plots.ipynb` reproduces in the main text (Figures 1 -- 5).

* `Synthetic/Fit.ipynb` reproduces in Appendices F, G (Figures 6 -- 20).

* `gnomAD/Plots.ipynb` reproduces in Appendix H (Figures 21 -- 38).

Expected run-time

Approximate time needed to reproduce the analyses on a standard desktop machine:

<1 minute

1-10 minutes

10-60 minutes

1-8 hours

>8 hours

Not feasible to run on a desktop machine, as described here:

### Additional documentation (optional)

# Notes (optional)

All analysis can be reproduced by running the python code at https://github.com/lorenzomasoero/ScaledProcesses
